# Neutrophil NADPH oxidase breaks the inflammatory IL-1β/IL-17A circuit to enhance pathogen clearance during respiratory virus infections

**DOI:** 10.1038/s41467-026-76327-4

**Published:** 2026-08-05

**Authors:** Aderonke Sofoluwe, Angelos Petropoulos, Zehra Fatima Ali-Khan, Abhilesh Salil Goomanee, Simon J. Cleary, Annika Warnatsch

**Affiliations:** 1 Peter Gorer Department, King’s College London, London, UK; 2 Institute of Pharmaceutical Science, King’s College London, London, UK

**Keywords:** Neutrophils, Infection, Acute inflammation, Interleukins

## Abstract

Respiratory virus infections are invariably accompanied by an increase in oxidative stress through elevated production of Reactive Oxygen Species (ROS), which contribute to both host defence and pathogenesis. Using mouse models, we identify neutrophil NADPH Oxidase 2 (Nox2) as the major early source of ROS during Influenza A Virus (IAV) infection. Surprisingly, neutrophil Nox2-derived ROS display multifaceted effects, not only unleashing oxidative stress but also limiting pro-inflammatory IL-1β signalling. Absence of neutrophil Nox2 enhances IL-1β production, promoting the proliferation of IL-17-producing gamma delta (γδ) T cells. This early self-amplified augmentation of the IL-1β/IL-17 axis is associated with increased viral burden and reduced IFNα expression in the lung. We extend our findings to humans. Similar patterns of ROS production and cytokine regulation are observed in human neutrophils when exposed to IAV and the viral RNA analogue poly(I:C). Our discovery highlights that ROS, often associated with harm, play a dual role by regulating cytokine signalling and thus influencing the immune response against respiratory viruses.

## Abstract (web summary)

Reactive oxygen species are associated with lung damage during respiratory virus infections, but detailed mechanism remains to be explored. The authors here identify a role for neutrophil-derived ROS in controlling inflammation and supporting antiviral immunity.

## Introduction

Respiratory viral infections such as influenza A virus (IAV) pose major global health challenges due to their high prevalence and substantial impact on public health. The innate immune system serves as a critical first line of defence, with epithelial and resident immune cells detecting viral components via pattern recognition receptors (PRRs), triggering production of type I interferons (IFNs), cytokines and chemokines^1^. These mediators initiate antiviral programs, activate immune cells and recruit additional effectors, including neutrophils and NK cells, to the site of infection.

Neutrophils rapidly infiltrate the lungs following IAV infection and contribute to host defence through phagocytosis, degranulation, neutrophil extracellular traps (NETs) and production of reactive oxygen species (ROS)^2^. Neutrophil depletion studies in mice have shown important roles for neutrophils in controlling viral replication and directing subsequent immune responses^3–5^. However, their overactivation can lead to pulmonary inflammation and damage, reflecting the need to balance effective viral defence with potential tissue injury for efficient immunity^6,7^.

The induction of oxidative stress via increased ROS production is a critical component of the pathophysiology of respiratory virus infections. Understanding the dual nature of ROS in these infections is crucial, as they are not only damaging agents but also play pivotal roles in immune signalling, cytokine production, inflammation and cell death^8^. Production of radicals and redox imbalance increase with age^9^, and so does susceptibility to fatal outcomes to virus infection, such as IAV and SARS-CoV-2. This complex interplay highlights the importance of understanding both the detrimental and beneficial effects of ROS, particularly in the context of viral replication and associated inflammation.

The enzyme NADPH oxidase 2 (Nox2) is a multi-subunit complex consisting of membrane-bound subunits gp91^phox^ (encoded by gene Nox2) and p22^phox^, along with cytosolic components p47^phox^, p67^phox^, p40^phox^ and the small GTPase Rac2. Upon activation, these subunits assemble and catalyse the generation of superoxide from oxygen^10^. This rapid ROS production is central to neutrophil antimicrobial functions. However, besides their microbicidal functions, Nox2-derived ROS act as signalling molecules with the capacity to regulate cellular pathways through reduction-oxidation (redox) regulation of target proteins such as transcription factor NFκB and inflammasome components^11^. Thus, the balance of Nox2 activity is crucial, with its dysregulation being implicated in severe disease outcomes in respiratory viral infections such as respiratory syncytial virus (RSV)^12^, IAV^13^ and SARS-CoV-2^14^.

Studies in mice with complete knockout of Nox2 have demonstrated slightly improved clearance of lung influenza infection with increased cytokine levels, concluding that absence of Nox2 improved the resolution of IAV infection but not inflammation^15,16^. Notably, macrophage-derived endosomal ROS have been reported to suppress toll-like receptor 7 (TLR7)-mediated interferon production, through oxidation of a highly conserved cysteine residue in TLR7^17^. However, macrophages generate significantly lower ROS than neutrophils. This highlights the need to understand how neutrophil-specific Nox2-derived ROS influence immunity during viral infections.

In various inflammatory models, Nox2 activity has been implicated in dampening proinflammatory cytokine production^18–22^. In neutrophils, redox regulation of NFκB^18^ and caspase-1^19^ modulates IL-1β and IL-8 production^20,21^. Furthermore, neutrophil-derived ROS have been shown to exert suppressive effects on neighbouring immune cells, such as CD8 T cells^23^, megakaryocytes^24^ and γδ T cells^25–27^ in a contact-dependent manner.

Despite these insights, the specific regulatory role of neutrophil-derived ROS during respiratory viral infections remains undefined. Here, we use a neutrophil-specific Nox2-deficient mouse model to dissect the impact of neutrophilic ROS on early immune responses to IAV. We show that neutrophil Nox2 regulates antiviral immune responses by modulating IL-1β and IL-17A signalling. Our results highlight Nox2 as the predominant source of ROS during early phases of respiratory virus infections and its unique role in modulating inflammatory signalling pathways. We demonstrate how neutrophil-derived ROS affect γδ T cell proliferation and function. In the absence of neutrophil Nox2, increased neutrophil-derived IL-1β drives proliferation of γδ17 T cells. This early amplification of the IL-β/IL-17 axis counteracts the antiviral interferon response in IAV-infected mice. Additionally, viral infection induced a self-perpetuating loop between human blood-derived IL-1β-producing neutrophils and IL-17-secreting γδ T cells ex vivo. By elucidating the dual role of ROS, our results suggest potential therapeutic strategies that involve modulating ROS levels to balance antiviral immunity and inflammation.

## Results

### Neutrophil Nox2 is a predominant source of ROS early after IAV lung infection

To study the effects of the neutrophil oxidative burst without disrupting ROS production in other cells, we generated *Ly6G-Cre*^*+/−*^ tdTomato *Nox2*^*fl/fl*^ mice. *Ly6G-Cre* mice provide a selective system to restrict gene deletion to neutrophils^28^, and unlike other Cre systems that induce recombination in neutrophils (*Mrp8-Cre* and *LysM-Cre*), does not also exhibit broader activity in myeloid progenitors^29–31^. To examine the critical window during which neutrophils are recruited to IAV-infected lungs, we focussed on the early immune response to IAV infection (days 1–5 post-infection)^3^.

To assess Nox2 deletion efficiency, naïve blood leukocytes were analysed by flow cytometry by gating on Live CD45^+^, CD11b^+^ cells and assessing tdTomato and Nox2 expression. Flow cytometric analysis revealed altered Nox2-FITC signal distributions in *Ly6G-Cre*^*+/−*^ and *Ly6G-Cre*^*+/−*^ *Nox2*^*fl/fl*^ mice compared with *Ly6G-Cre*^*−/−*^ *Nox2*^*fl/fl*^ controls (Supplementary Fig. 1A, B). Reduced Nox2 signal was most pronounced within the tdTomato^+^ compartment of *Ly6G-Cre*^*+/−*^ *Nox2*^*fl/fl*^ mice. However, partial reductions in Nox2 signal were also observed within tdTomato^−^ cells and in *Ly6G-Cre*^*+/−*^ control mice lacking the floxed *Nox2* allele. These findings indicated that tdTomato/Cre status and reporter-based gating complicated interpretation of Nox2 expression by flow cytometry alone.

We therefore further examined the relationship between tdTomato reporter expression and Ly6G-lineage neutrophils by immunofluorescence analysis of lung sections. This demonstrated incomplete overlap between tdTomato expression and Ly6G staining, with a proportion of Ly6G^+^ cells lacking detectable tdTomato expression and tdTomato^+^ Ly6G^*−*^ cells observed within the same tissue sections (Supplementary Fig. 1C, D).

To more conclusively determine the cell type specificity of Nox2 deletion, we next flow-sorted lung myeloid populations from *Ly6G-Cre*^*+/−*^ tdTomato, *Ly6G-Cre*^*−/−*^ *Nox2*^*fl/fl*^ and *Ly6G-Cre*^*+/−*^ tdTomato *Nox2*^*fl/fl*^ mice at day 3 post-IAV infection. Neutrophils were identified as live CD45^+^, CD11b^+^, Ly6G^+^, CD64^*−*^, monocytes as live CD45^+^, CD11b^+^, Ly6G^*−*^, Ly6C^hi^ and macrophages as live CD45^+^, CD11b^+^, Ly6G^*−*^, CD64^+^ cells. Quantitative PCR analysis confirmed that Nox2 deletion was restricted to the neutrophil population (Supplementary Fig. 2A, B), with near-complete loss of *Cybb* expression in neutrophils from *Ly6G-Cre*^*+/−*^ tdTomato *Nox2*^*fl/fl*^ mice, whereas monocytes or macrophages retained Nox2 expression comparable to control mice.

A recently identified subset of Ly6G^+^ CD64^+^ macrophages has been reported to emerge from day 5 post-IAV infection and peak around day 10 during the resolution phase^32^. At day 3 post-infection, this population was rare in our model (2 ± 0.8% of live CD45^+^, CD11b^+^ cells), precluding reliable flow sorting and downstream PCR analysis. Therefore, we cannot exclude recombination within this Ly6G^+^ CD64^+^ macrophage population. However, its low abundance at day 3 post-infection makes it unlikely to substantially contribute to the observed phenotype.

To further confirm neutrophil-specific Nox2 deletion at the protein level, lung sections were stained for Nox2 subunit gp91^phox^ by immunofluorescence (Supplementary Fig. 2C, D). Nox2 was not exclusively expressed by neutrophils, but was also detected in multiple Ly6G^*−*^ populations within lung tissue. Neutrophil-associated gp91^phox^ staining (white arrows) was readily observed in *Ly6G-Cre*^*+/−*^ tdTomato control mice, but was absent in *Ly6G-Cre*^*+/−*^ tdTomato *Nox2*^*fl/fl*^ *mice*. Quantification of gp91^phox^ fluorescence intensity in tdTomato^+^ neutrophils confirmed a significant reduction in Nox2 protein expression in *Ly6G-Cre*^*+/−*^ tdTomato *Nox2*^*fl/fl*^ mice compared with controls (Supplementary Fig. 2D).

**Fig. 1:**
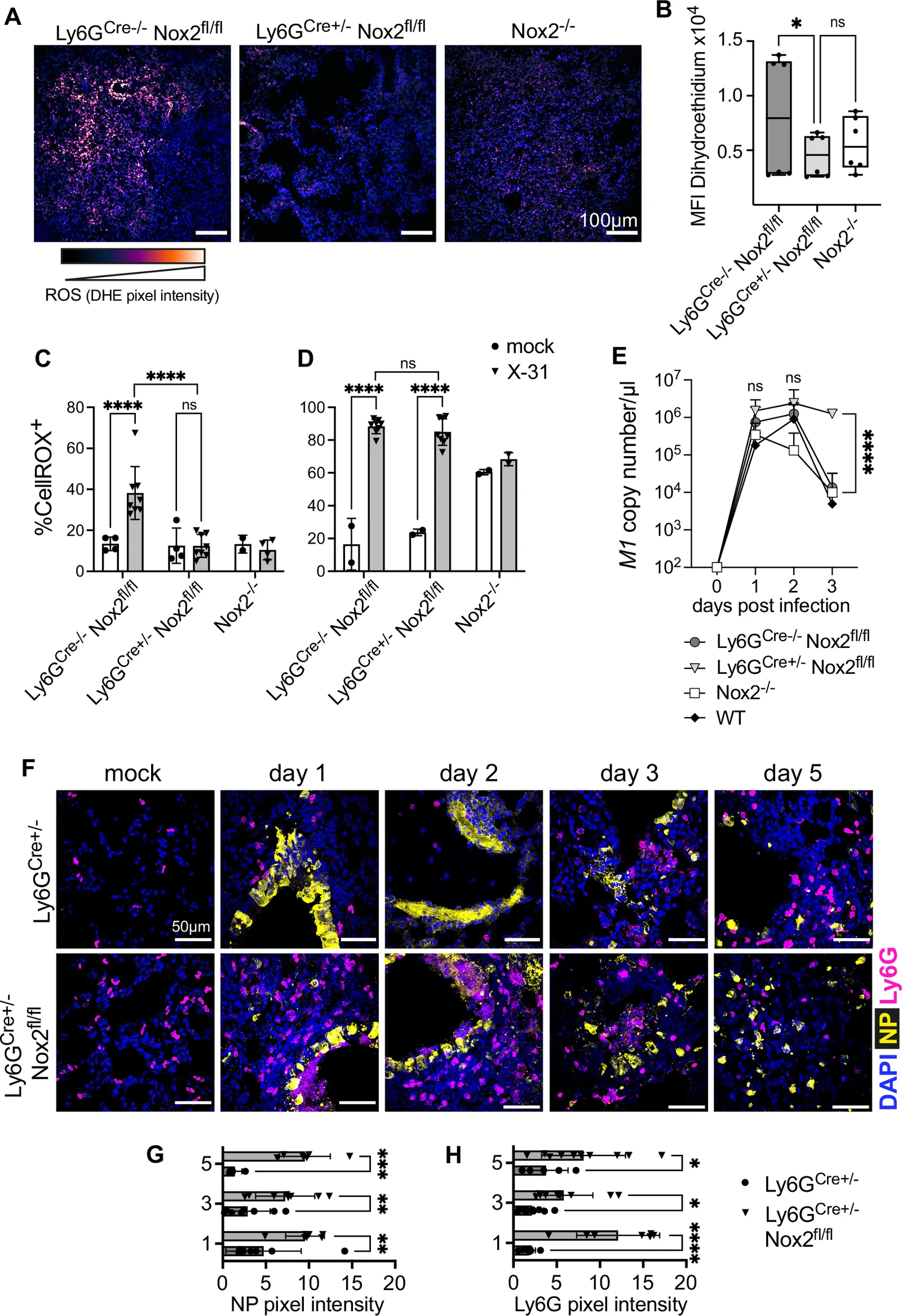
Neutrophil Nox2-derived ROS play a crucial role in viral clearance during early IAV infection. Male and female *Ly6G-Cre*^*−*/*−*^ *Nox2*^*fl/fl*^, *Ly6G-Cre*^+/*−*^ *Nox2*^*fl/fl*^ and *Nox2*^*−/−*^ mice were infected with IAV strain X-31 at 3 × 10^4^ TCID_50_ or PBS (mock) in 30 µl intranasally. **A**, **B** Representative immunofluorescence micrographs of lung sections stained with dihydroethidium (DHE) for superoxide 18 h after infection (**A**) and DHE geometric mean fluorescence intensity (MFI) in live CD45^+^ lung cells (**B**). Box-and-whisker plot displays the median (centre line), the interquartile range (box, 25th–75th percentiles) and the minimum and maximum values (whiskers) (*n* = 6). **C** Percentage of CellROX^+^ cells gated as live CD45^+^ in lungs 3 days post-infection (*n* = 4 mock and *n* = 8 X-31 for both *Ly6G-Cre*^*−*/*−*^ *Nox2*^*fl/fl*^ and *Ly6G-Cre*^+/*−*^ *Nox2*^*fl/fl*^, *n* = 2 mock and *n* = 4 X-31 for *Nox2*^*−/−*^). Also see Supplementary Fig. 3A. **D** Percentage of CellROX^+^ cells gated as live CD45^*−*^ in lungs 3 days post-infection (*n* = 2 mock and *n* = 8 X-31 for both *Ly6G-Cre*^*−*/*−*^ *Nox2*^*fl/fl*^ and *Ly6G-Cre*^+/*−*^ *Nox2*^*fl/fl*^, *n* = 2 mock and *n* = 2 X-31 for *Nox2*^*−/−*^). Also see Supplementary Fig. 3A. **E** RT-qPCR of influenza matrix *M1* RNA in whole lungs at indicated time points post-infection (*n* = 6 day 1, *n* = 7 day 2 and *n* = 8 day 3). Also see Supplementary Fig. 3B. **F** Representative immunofluorescence micrographs of lung sections stained for IAV nucleoprotein (NP, yellow), neutrophils (Ly6G, magenta) and nuclei (DAPI, blue) at indicated time points post-infection. Scale bar 50 µm. **G** NP pixel intensity per field of view per animal (day 1: *n* = 7 *Ly6G-Cre*^+/*−*^ and *n* = 6 *Ly6G-Cre*^+/*−*^ *Nox2*^*fl/fl*^, day 3: *n* = 8 both genotypes, day 5: *n* = 4 *Ly6G-Cre*^+/*−*^ and *n* = 6 *Ly6G-Cre*^+/*−*^ *Nox2*^*fl/fl*^). **H** Ly6G pixel intensity per field of view per animal (day 1: *n* = 7 *Ly6G-Cre*^+/*−*^ and *n* = 6 *Ly6G-Cre*^+/*−*^ *Nox2*^*fl/fl*^, day 3: *n* = 8 *Ly6G-Cre*^+/*−*^ and *n* = 10 *Ly6G-Cre*^+/*−*^ *Nox2*^*fl/fl*^, day 5: *n* = 5 *Ly6G-Cre*^+/*−*^ and 9 *Ly6G-Cre*^+/*−*^ *Nox2*^*fl/fl*^). Bars represent mean ± SD of 2–3 independent experiments. Each data point represents one animal (**B**–**D**, **G**, **H**). *p* values are indicated, *<0.05, **<0.01, ***<0.001, ****<0.0001, ns not significant. *p* values were determined using two-way ANOVA followed by Tukey’s post-test (**B**–**E**) and two-way ANOVA followed by Sidak’s post-test (**G**, **H**). Source data are provided as a Source Data file.

To analyse the contribution of neutrophil Nox2-mediated superoxide production to overall ROS levels in IAV-infected lungs, we infected *Ly6G-Cre*^*+/−*^ *Nox2*^*fl/fl*^ mice with IAV strain X-31 at 3 × 10^4^ TCID_50_, a mild and self-resolving infectious dose, and measured superoxide production in the lung by Dihydroethidium (DHE) staining 18 h post-infection (Fig. 1A, B). Compared to IAV-infected *Ly6G-Cre*^*−/−*^ *Nox2*^*fl/fl*^ control mice, DHE staining was 1.8-fold reduced in *Ly6G-Cre*^*+/−*^ *Nox2*^*fl/fl*^ mice, comparable to *Nox2*^*−/−*^ mice. Moreover, we assessed hydrogen peroxide levels by CellROX Green staining. Percentage of Live CD45^+^ CellROX^+^ immune cells in the lungs of IAV-infected *Ly6G-Cre*^*−/−*^ *Nox2*^*fl/fl*^ mice increased to 38.2 ± 18.7% at day 3 post-infection while in *Ly6G-Cre*^*+/−*^ *Nox2*^*fl/fl*^ mice only 12.4 ± 4.3% of lung immune cells were CellROX^+^ comparable to mock-infected control and IAV-infected *Nox2*^*−/−*^ mice (13.4 ± 2.9% and 10.4 ± 4.2%, respectively; Fig. 1C). The CD45^*−*^ non-immune cell compartment showed unimpaired induction of oxidative stress in *Ly6G-Cre*^*+/−*^ *Nox2*^*fl/fl*^ mice (Fig. 1D and Supplementary Fig. 3A). Baseline CellROX^+^ intensity in the CD45^*−*^ compartment of *Nox2*^*−/−*^ mice was elevated, possibly due to compensatory ROS production in global knockouts via Nox2-independent pathways, however no upregulation was found post-IAV infection. The similarity between neutrophil-specific Nox2-deficient and *Nox2*^*−/−*^ animals revealed that neutrophil Nox2 is the predominant source of ROS in the immune cell compartment between 1 and 3 days post-IAV infection, while ROS production in the non-immune compartment remained intact.

### Absence of neutrophil Nox2 impairs viral clearance and increases lung inflammation

Previous work has demonstrated improved resolution of IAV infection in *Nox2*^*−/−*^ mice^15,16^. To examine how neutrophil Nox2-derived ROS production affects virus replication we measured viral matrix mRNA in the lung after infection with IAV strain X-31 at 3 × 10^4^ TCID_50_ (Fig. 1E and Supplementary Fig. 3B). After initial increase of viral burden 48 h post-infection in all mouse strains, viral burden rapidly declined in *Nox2*^*−/−*^ as well as *Ly6G-Cre*^*−/−*^ *Nox2*^*fl/fl*^ control mice at day 3 post-infection, while viral burden did not significantly reduce in *Ly6G-Cre*^*+/−*^ *Nox2*^*fl/fl*^ mice. We confirmed diminished virus clearance in *Ly6G-Cre*^*+/−*^ *Nox2*^*fl/fl*^ mice by immunofluorescence staining for viral nucleoprotein (NP, yellow) in lung sections of infected mice (Fig. 1F, quantified in 1G). Interestingly, increased accumulation of neutrophils (Ly6G, magenta; quantified in Fig. 1H) was observed around infected bronchioli, and larger amounts of viral nucleoprotein persisted in infected lungs of *Ly6G-Cre*^*+/−*^ *Nox2*^*fl/fl*^ mice until day 5 post-infection.

Reduced virus clearance was further corroborated by significantly increased weight loss in IAV-infected *Ly6G-Cre*^*+/−*^ *Nox2*^*fl/fl*^ mice (Supplementary Fig. 3C). Histopathological analysis revealed a transient increase in inflammation in *Ly6G-Cre*^*+/−*^ *Nox2*^*fl/fl*^ mice from day 1 to day 3 compared to *Ly6G-Cre*^*−/−*^ *Nox2*^*fl/fl*^ control mice as measured by nuclei count and scoring (Supplementary Fig. 3D–F). Inflammation in *Ly6G-Cre*^*−/−*^ *Nox2*^*fl/fl*^ mice concentrated around bronchioli. In contrast, lung damage spread further to alveoli in *Ly6G-Cre*^*+/−*^ *Nox2*^*fl/fl*^ mice. Overall, neutrophil-specific Nox2-deficient mice exhibit heightened lung inflammation and damage, coupled with compromised virus clearance.

### The immune response to influenza A virus is shifted towards the IL-1β/IL-17A axis in neutrophil-specific Nox2-deficient mice

**Fig. 2:**
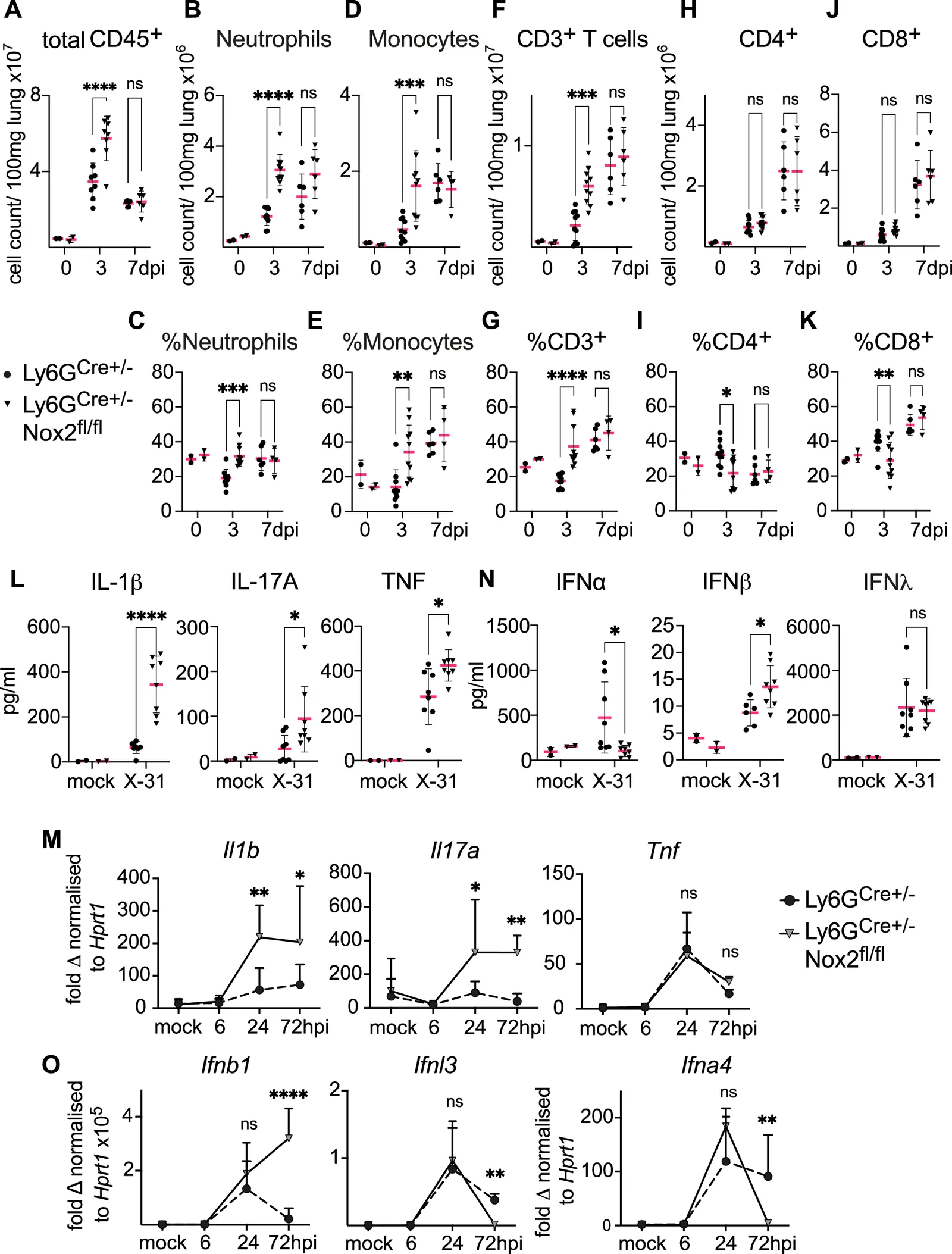
Increased activation of IL-1β /IL-17-axis in IAV-infected neutrophil-specific Nox2-deficient mice. Male and female *Ly6G-Cre*^+/*−*^, *Ly6G-Cre*^*−*/*−*^ *Nox2*^*fl/fl*^ and *Ly6G-Cre*^+/*−*^ *Nox2*^*fl/fl*^ mice were infected with 3 × 10^4^ TCID_50_ IAV strain X-31 or PBS (mock) in 30 µl intranasally. Absolute count per 100 mg lung tissue and frequency of Live CD45^+^ leukocytes (**A**), neutrophils gated as CD11b^hi^, Ly6G^tdTom^ (**B**, **C**), inflammatory monocytes gated as CD11b^hi^, Ly6G^*−*^, Ly6C^hi^, MHCII^+^ (**D**, **E**), CD3e^+^ T cells (**F**, **G**), CD4^+^ (**H**, **I**) and CD8^+^ αβ T cells (**J**, **K**) at indicated time points (days) post infection (day 0: *n* = 2, day 3: *n* = 10, day 7: *n* = 6). Also see Supplementary Fig. 4. Cytokines (**L**) and interferons (**N**) were quantified in bronchoalveolar lavage 3 days post-infection by ELISA (mock: *n* = 2, X-31: *n* = 8). Cytokine (**M**) and interferon (**O**) mRNA expression in fold change from naïve, normalised to *Hprt1* in lung tissue, was quantified at indicated time points by RT-qPCR (mock: *n* = 3, X-31: *n* = 6). Bars represent mean ± SD of 2–5 independent experiments. Each data point represents one animal (**A**–**L**, **N**). *p* values are indicated, *<0.05, **<0.01, ***<0.001 and ****<0.0001, ns not significant. *p* values were determined using two-way ANOVA and Sidak’s post-test (**A**–**L**, **N**) and two-tailed Mann–Whitney test (**M**, **O**). Source data are provided as a Source Data file.

The composition of cellular infiltrates in lung tissue was investigated by flow cytometry. Total lung cell counts were 1.5-fold increased in *Ly6G-Cre*^*+/−*^ *Nox2*^*fl/fl*^ compared to *Ly6G-Cre*^*+/−*^ control mice 3 days post-infection (Fig. 2A and gating strategy, Supplementary Fig. 4A, B). Predominantly, numbers of neutrophils and inflammatory Ly6C^hi^ monocytes were increased in IAV-infected *Ly6G-Cre*^*+/−*^ *Nox2*^*fl/fl*^ mice (absolute counts and frequency Fig. 2B–E). We further observed elevated counts of T cells (absolute counts and frequency Fig. 2F, G). Interestingly, CD8^+^ and CD4^+^ αβ T cells cannot alone account for the increased number of CD3^+^ T cells (absolute counts and frequency Fig. 2H–K). No changes were observed in cell counts and frequency of alveolar macrophages, conventional dendritic cells or NK cells (Supplementary Fig. 5A–F and gating strategy 5K). Total macrophage numbers and frequency were slightly elevated in *Ly6G-Cre*^*+/−*^ *Nox2*^*fl/fl*^ mice at day 3 (Supplementary Fig. 5G, H), although this did not include Ly6G⁺ macrophages, which remained low in number and unchanged between genotypes (Supplementary Fig. 5I, J). No significant differences in lung immune cell counts between *Ly6G-Cre*^*+/−*^ *Nox2*^*fl/fl*^ and *Ly6G-Cre*^*+/−*^ control mice were observed 7 days post-IAV infection, and total lung cell counts declined in both strains (Fig. 2A–K).

Strikingly, overall chemokine and cytokine release was enhanced in the bronchoalveolar lavage (BAL) of IAV-infected *Ly6G-Cre*^*+/−*^ *Nox2*^*fl/fl*^ mice (Supplementary Fig. 6A, B). Specifically, chemokines and cytokines controlling the recruitment of neutrophils and monocytes, as well as B and T cell proliferation and activation, were elevated 3 days post-infection. While factors supporting resolution of inflammation were downregulated (e.g. Reg3G, EGF and FGF), indicating an overall dysregulated immune response in IAV-infected *Ly6G-Cre*^*+/−*^ *Nox2*^*fl/fl*^ mice. We confirmed increased presence of pro-inflammatory IL-1β, IL-17A and TNF protein in BAL at day 3 (Fig. 2L). Transcription of *Il1b* and *Il17a* mRNA increased as early as day 1 and remained elevated in *Ly6G-Cre*^*+/−*^ *Nox2*^*fl/fl*^ mice at day 3, while *Tnf* transcripts in lung tissue remained comparable between both mouse strains (Fig. 2M), suggesting enhanced post-transcriptional processing and release of TNF protein in the absence of neutrophil Nox2^33^.

Interestingly, interferon β1 (IFNβ1) expression increased in parallel with IL-1β and IL-17A, consistent with previously reported NFκB-mediated co-regulation of *Ifnb* with *Il1b* (Fig. 2N)^34^. In contrast, *Ifnα4* mRNA and protein expression declined rapidly after initial up-regulation at day 1 in *Ly6G-Cre*^*+/−*^ *Nox2*^*fl/fl*^ mice, while remaining elevated in control animals (Fig. 2N, O). This aligned with expression levels of interferon-stimulated genes *Isg15*, *Ifit3* and *Oas1a* (Supplementary Fig. 6C–E). Of note, IFNλ3 protein levels, measured by ELISA, were not significantly reduced despite the modest reduction in mRNA, suggesting post-transcriptional regulation may preserve local IFNλ protein levels.

Furthermore, mRNA expression of *Ifng* was also reduced at day 3 post-infection in *Ly6G-Cre*^*+/−*^ *Nox2*^*fl/fl*^ mice (Supplementary Fig. 6F). Additionally, we observed elevated amounts of prostaglandin E2 (PGE2) protein in BAL (Supplementary Fig. 6G), a known inhibitor of type I interferon production and antiviral immunity induced by IL-1β^35^. In conclusion, neutrophil-specific Nox2 deficiency skews the lung cytokine profile towards IL-1β and IL-17A production, while impairing sustained expression of anti-viral type I interferon. The observed imbalance may reflect a dysregulated immune response that fails to clear virus effectively, while promoting tissue pathology in these mice.

### Enhanced γδ T cell-driven IL-17A response in Nox2-deficient mice

**Fig. 3:**
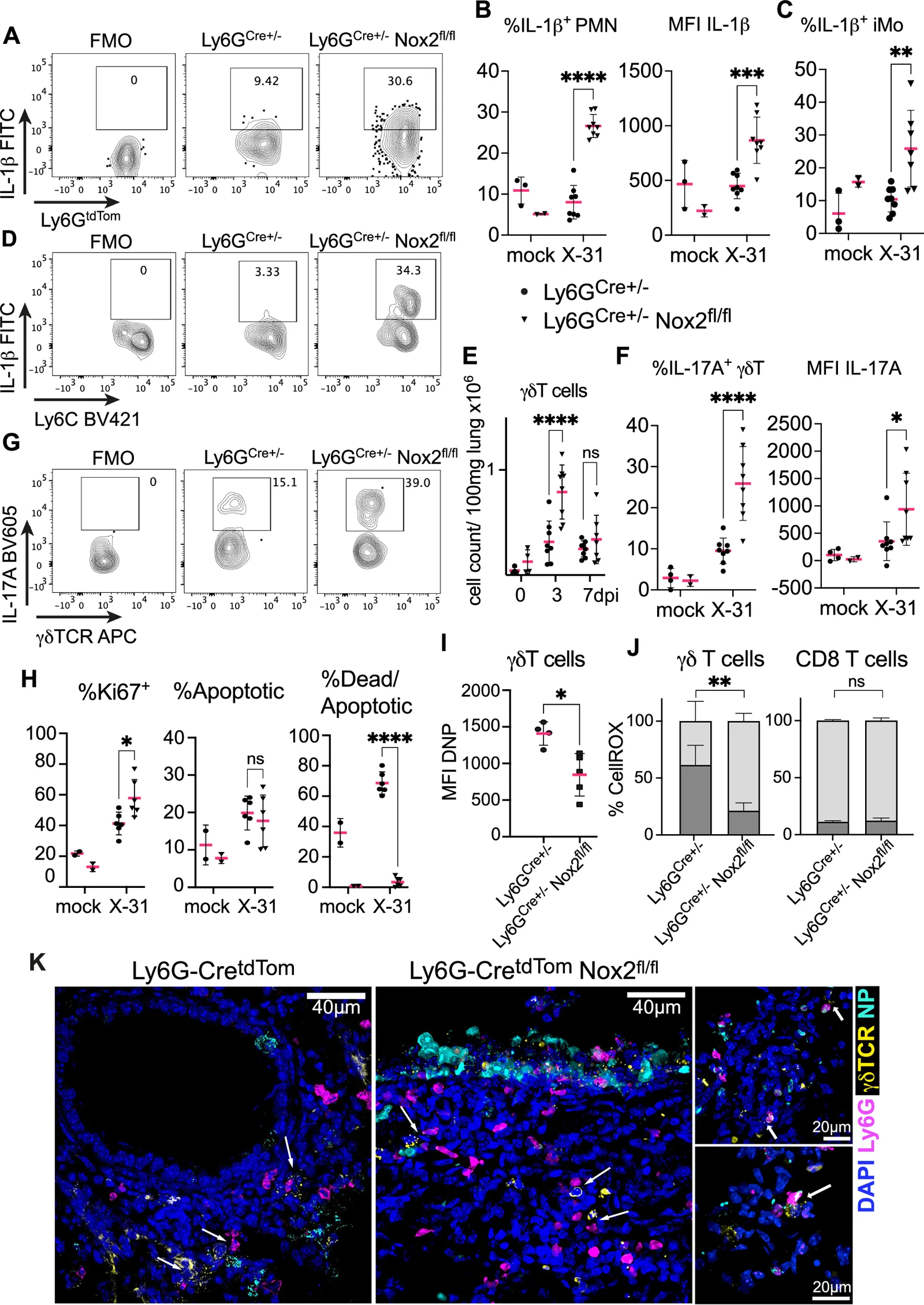
Enhanced γδ T cell-driven IL-17 response in IAV-infected Nox2-deficient animals. Male and female *Ly6G-Cre*^+/*−*^ and *Ly6G-Cre*^+/*−*^ *Nox2*^*fl/fl*^ mice were infected with 3 × 10^4^ TCID_50_ IAV strain X-31 or PBS (mock) in 30 µl intranasally for indicated time points. **A** Representative flow cytometry plots of IL-1β^+^ neutrophils from *Ly6G-Cre*^+/*−*^ (middle panel) and *Ly6G-Cre*^+/*−*^ *Nox2*^*fl/fl*^ (right panel) mice at 3 days post-infection. Neutrophils were gated as live CD45^+^, CD11b^+^ and Ly6G^tdTom^. IL-1β^+^ gates were set based on fluorescence minus one (FMO) controls (left panel). **B** Frequency (left panel) and geometric mean fluorescence intensity (MFI, right panel) of IL-1β^+^ neutrophils at 3 days post-infection (mock: *n* = 3 *Ly6G-Cre*^+/*−*^ and *n* = 2 *Ly6G-Cre*^+/*−*^ *Nox2*^*fl/fl*^, X-31: *n* = 8 both genotypes). Also see Supplementary Fig. 7A. **C** Frequency of IL-1β^+^ inflammatory monocytes gated as live CD45^+^, CD11b^+^, Ly6G^*−*^, Ly6C^hi^ and MHCII^+^ at 3 days post-infection (mock: *n* = 3 *Ly6G-Cre*^+/*−*^ and *n* = 2 *Ly6G-Cre*^+/*−*^ *Nox2*^*fl/fl*^, X-31: *n* = 8 *Ly6G-Cre*^+/*−*^ and *n* = 7 *Ly6G-Cre*^+/*−*^ *Nox2*^*fl/fl*^). **D** Representative flow cytometry plots of IL-1β^+^ inflammatory monocytes at 3 days post-infection. IL-1β^+^ gates were set based on FMO controls (left panel). **E** Number of γδ T cells gated as live CD45^+^, CD3e^+^ and γδTCR^+^ per 100 mg lung tissue at 3- and 7-days post-infection (mock: *n* = 4, day 3: *n* = 8 both genotypes, day 7: *n* = 8 *Ly6G-Cre*^+/*−*^ and *n* = 7 *Ly6G-Cre*^+/*−*^ *Nox2*^*fl/fl*^). Also see Supplementary Fig. 9C. **F** Frequency (left panel) and MFI (right panel) of IL-17A^+^ γδ T cells gated as live CD45^+^, CD3e^+^ and γδTCR^+^ (mock: *n* = 4 *Ly6G-Cre*^+/*−*^ and *n* = 2 *Ly6G-Cre*^+/*−*^ *Nox2*^*fl/fl*^, X-31: *n* = 8, both genotypes). Also see Supplementary Fig. 9D. **G** Representative flow cytometry plots of IL-17A^+^ γδ T cells at 3 days post-infection. IL-17A^+^ gates were set based on FMO controls (left panel). **H** Frequency of Ki67^+^ (left panel), Apotracker^+^ (middle panel) and ZombieViolet^+^ Apotracker^+^ double-positive (right panel) γδ T cells at 3 days post-infection (mock: *n* = 2, X-31: *n* = 6). Also see Supplementary Fig. 11A, B. **I** MFI of anti-dinitrophenylhydrazone (DNP)-staining at 3 days post-infection, measuring protein carbonylation as a readout of protein oxidation (*n* = 4 *Ly6G-Cre*^+/*−*^, *n* = 5 *Ly6G-Cre*^+/*−*^ *Nox2*^*fl/fl*^). **J** Frequency of CellROX^+^ γδ T (left panel, *n* = 6) and CD8 T cells (right panel, *n* = 3) at 3 days post-infection. Also see Supplementary Fig. 11C, D. **K** Representative immunofluorescence micrographs of lung sections stained for IAV nucleoprotein (NP, cyan), γδ T cells (γδTCR, yellow), neutrophils (Ly6G, magenta) and nuclei (DAPI, blue). Scale bars 40 µm (large panels). Magnifications of γδ T cell-neutrophil interactions in *Ly6G-Cre*^+/*−*^ *Nox2*^*fl/fl*^ mice. Scale bars 20 µm (left panels). White arrows highlight γδ T cells and neutrophils in close proximity. Repeated for 3 animals of each genotype. Bars represent mean ± SD of 2–4 independent experiments. Each data point represents an animal. *p* values are indicated, *<0.05, **<0.01, ***<0.001 and ****<0.0001, ns not significant. *p* values were determined using two-way ANOVA and Sidak’s post-test (**B**–**F**) and two-tailed Mann–Whitney test (**G**, **H**). Source data are provided as a Source Data file.

We performed intracellular cytokine staining to determine the cellular source of elevated proinflammatory cytokines. We identified neutrophils and inflammatory Ly6C^high^ monocytes as IL-1β producers in *Ly6G-Cre*^*+/−*^ *Nox2*^*fl/fl*^ mice at day 3 post-infection (frequency and geometric mean fluorescence intensity Fig. 3A–D, absolute count and isotype control Supplementary Fig. 7A, B). Moreover, conventional dendritic cells and alveolar macrophages were found to produce IL-1β (Supplementary Fig. 7B–G). Notably, the absolute number of TNF-producing alveolar macrophages was increased in *Ly6G-Cre*^*+/−*^ *Nox2*^*fl/fl*^ mice (Supplementary Fig. 7B, F, G). Alveolar macrophage-derived TNF has been reported previously to prime neutrophils for IL-1β production in a model of invasive pneumococcal disease^36^. Furthermore, depletion of neutrophils through repeated injection of anti-Ly6G antibody in *Ly6G-Cre*^*+/−*^ *Nox2*^*fl/fl*^ mice confirmed that neutrophils initiated the increase of *Il1b* mRNA and protein in lungs 3 days post-IAV infection (Supplementary Fig. 7H–J). These findings support a model in which Nox2-deficient neutrophils instigate a proinflammatory cascade that promotes IL-1β production not only within neutrophils themselves but also by activating downstream immune cell populations such as monocytes and macrophages.

To further investigate the phenotype of Nox2-deficient neutrophils during infection, we performed additional immunophenotyping of lung neutrophils at day 3 post-IAV infection in *Ly6G-Cre*^*+/−*^ and *Ly6G-Cre*^*+/−*^ *Nox2*^*fl/fl*^ mice (Supplementary Fig. 8A–G). Nox2-deficient neutrophils displayed increased expression of CD63 and Siglec-F (frequency and MFI, Supplementary Fig. 8A, B), suggesting enhanced degranulation and a shift toward an aged or tissue-adapted phenotype^37^. In contrast, CD11a, CD44 and MHC class II levels were reduced (Supplementary Fig. 8C–E), indicative of altered adhesion, migration and low antigen-presenting potential. Expression of CXCR2 and CD11b (Supplementary Fig. 8F, G) remained unchanged between genotypes. These phenotypic alterations support a broader role for Nox2 in neutrophil programming beyond ROS generation and align with the increased IL-1β production and pro-inflammatory profile observed in Nox2-deficient neutrophils.

Neither CD4 nor CD8 αβ T cells were found to express significant amounts of IL-17A upon IAV infection in *Ly6G-Cre*^*+/−*^ and *Ly6G-Cre*^*+/−*^ *Nox2*^*fl/fl*^ mice (Supplementary Fig. 9A, B). Since αβ T cells did not alone account for increased lung infiltration of CD3^+^ T cells at day 3, we further dissected the nature of lung-infiltrating T cells at this early time point. Strikingly, we observed a 2.5-fold elevated count of γδ T cells in *Ly6G-Cre*^*+/−*^ *Nox2*^*fl/fl*^ mice compared to *Ly6G-Cre*^*+/−*^ mice which rapidly declined at day 7 (absolute count Fig. 3E and frequency Supplementary Fig. 9C). Moreover, we identified γδ T cells as predominant source of elevated IL-17A at 3 days post-infection in *Ly6G-Cre*^*+/−*^ *Nox2*^*fl/fl*^ mice (frequency and geometric mean fluorescence intensity Fig. 3F, G, absolute count Supplementary Fig. 9D). While the typical virus-induced IFNγ expression^38^ was slightly decreased in γδ T cells (Supplementary Fig. 9E) as well as in CD4 and CD8 αβ T cells (Supplementary Fig. 9A, B).

In addition, we observed a marked increase of CD27^*−*^ γδ T cells and a reduction of CD27^+^ γδ T cells in the lungs of IAV-infected *Ly6G-Cre*^*+/−*^ *Nox2*^*fl/fl*^ mice (Supplementary Fig. 9F, G), consistent with the expansion of IL-17-producing γδ T cells. CD27^−^ γδ T cells represent a subset of γδ T cells specialised for IL-17A production during infection and inflammation^39^. Phenotypic analysis further revealed distinct alterations in surface marker expression (Supplementary Fig. 9H–M): CD27⁺ γδ T cells from *Ly6G-Cre*^*+/−*^ *Nox2*^*fl/fl*^ mice showed decreased expression of CXCR3, CD44 and PD-L1, which may indicate impaired chemotactic responsiveness, reduced activation or tissue retention and diminished immunoregulatory potential. Conversely, CD27^−^ γδ T cells displayed increased expression of CD71 and CD98 (Supplementary Fig. 9K, L), markers associated with enhanced metabolic activity and proliferative capacity. Together, these findings suggest that neutrophil-derived ROS via Nox2 influences the activation state, effector function and metabolic programming of γδ T cells during IAV infection.

We further examined the dynamics of DN αβ T cells. At 3 days post-infection, we observed a reduction in CD4^+^ and CD8^+^ αβ T cell frequencies, accompanied by a significant increase in the frequency and absolute number of DN αβ T cells gated as live CD45^+^, CD3^+^, TCRγδ^*−*^, CD4^*−*^, CD8^*−*^ in *Ly6G-Cre*^*+/−*^ *Nox2*^*fl/fl*^ mice (Supplementary Fig. 10A). By day 7, DN T cell frequency and numbers returned to control levels, indicating a transient accumulation during early infection. Intracellular cytokine staining did not detect IL-17A or IFNγ expression in DN αβ T cells at day 3 post-infection in either genotype (Supplementary Fig. 10B). Surface marker analysis revealed reduced CD62L^−^ frequency (Supplementary Fig. 10C), suggesting impaired effector transition or delayed activation. Lower MFI of CD25 and CD98 (Supplementary Fig. 10D–G) indicated diminished metabolic readiness, and decreased CCR2 and CXCR3 (Supplementary Fig. 10H–K) expression implied altered migratory potential in DN T cells from *Ly6G-Cre*^*+/−*^ *Nox2*^*fl/fl*^ mice. Additionally, we observed an increased frequency of CD27^+^ DN T cells (Supplementary Fig. 10L), consistent with a less differentiated phenotype. These findings suggest that Nox2-deficient neutrophils may stall T cell subset differentiation and contribute to the transient emergence of a functionally inert DN αβ T cell population.

Surging γδ T cell counts correlated with increased cell proliferation (measured by Ki67 staining, left panel), and while the frequency of single-stained apoptotic γδ T cells (middle panel) was not affected, the percentage of double-positive apoptotic dead cells (right panel) was significantly reduced in *Ly6G-Cre*^*+/−*^ *Nox2*^*fl/fl*^ mice (Fig. 3H and Supplementary Fig. 11A, B).

Confirming the changes in γδ T cell proliferation and cytokine expression depend on neutrophil Nox2-mediated ROS production, we found that protein oxidation in γδ T cells was 0.5-fold reduced in *Ly6G-Cre*^*+/−*^ *Nox2*^*fl/fl*^ mice by analysing protein carbonylation (Fig. 3I). Protein carbonylation occurs as a result of oxidative damage, and was quantified using 2,4-dinitrophenylhydrazine (DNPH) to convert carbonyl groups into stable dinitrophenyl (DNP) hydrazone adducts, that were detected using anti-DNP antibodies. Moreover, the percentage of CellROX^+^ γδ T cells was 0.35-fold reduced in *Ly6G-Cre*^*+/−*^ *Nox2*^*fl/fl*^ mice (left panel), while CD8 (right panel), CD4 and DN T cells, as well as NK cells, seemed unaffected by neutrophil ROS production, with generally low percentages of CellROX^+^ cells (Fig. 3J and Supplementary Fig. 11C, D). To substantiate a potential interaction between neutrophils and γδ T cells in IAV-infected lungs, we verified that both cell types are found in close proximity near sites of infection (Fig. 3K).

In conclusion, neutrophils and γδ T cells directly interact in IAV-infected lungs, where Nox2-derived ROS induce oxidative damage and apoptosis in γδ T cells and moreover suppress the proliferation of CD27^*−*^ γδ17 T cells.

### Nox2-deficient neutrophils induce γδ17 T cells

**Fig. 4:**
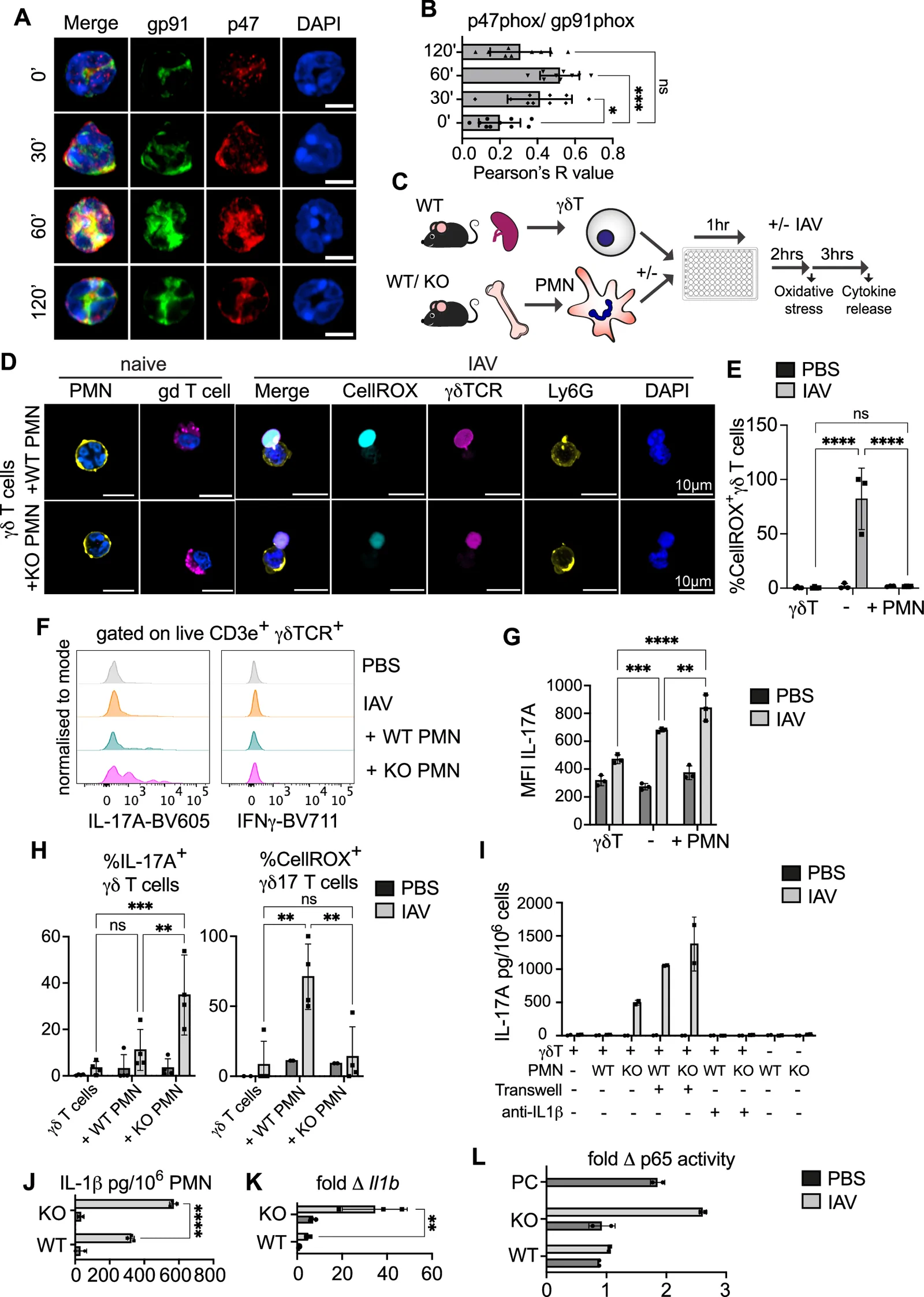
Nox2-deficient mouse neutrophils induce γδ17 T cells upon IAV exposure. γδ T cells isolated from the spleen of naïve C57Bl/6J mice were co-cultured ex vivo with WT (C57Bl/6) or KO (*Nox2*^*−/−*^) neutrophils (PMN) isolated from bone marrow at a ratio of 1:4 and were treated with PBS or stimulated with IAV strain PR8 at 0.2 MOI (multiplicity of infection). Also see Supplementary Fig. 12. **A** Representative immunofluorescence micrographs of WT neutrophils at indicated time points (minutes) post-stimulation stained for p47^phox^ (red), gp91^phox^ (green) and nuclei (DAPI, blue). Scale bar 5 µm. **B** Pearson’s *R* values for colocalisation of p47^phox^ and gp91^phox^ in mouse neutrophils stimulated with IAV for indicated time points per field of view (0′ and 30′: *n* = 10, 60′ and 120′: *n* = 9). **C** Schematic of experimental setup. γδ T cells were isolated from spleens of C57Bl/6J (wild-type) mice and cultured alone or with bone marrow-derived neutrophils from wild-type or *Nox2*^*−/−*^ mice. After 1 h of co-culture, cells were stimulated with IAV or mock-treated (PBS). Oxidative stress (CellROX staining) was assessed 2 h post-stimulation; cytokine release in cell culture supernatant was measured 5 h post-stimulation. **D** Representative immunofluorescence micrographs of ex vivo co-cultures 2 h post-stimulation with IAV or PBS (naïve) stained for oxidative stress (CellROX, cyan), neutrophils (Ly6G, magenta), γδ T cells (γδTCR, yellow) and nuclei (DAPI, blue). Scale bar 10 µm. **E** Frequency of CellROX^+^ γδ T cells 2 h post-stimulation cultured alone, in contact with WT or KO neutrophils (WT PMN: *n* = 3, KO PMN: *n* = 4). **F** Representative histograms of IL-17A^+^ (left panel) and IFNγ^+^ (right panel) γδ T cells gated as live CD45^+^, CD3e^+^, CD4^*−*^ and γδTCR^+^ 5 h post-stimulation with PBS or IAV, cultured alone and in contact with WT or KO neutrophils. **G** Geometric mean fluorescence intensity (MFI) of IL-17A+ γδ T cells 5 h post-stimulation with PBS or IAV, cultured alone and in contact with WT or KO neutrophils (*n* = 3). **H** Frequency of IL-17A^+^ γδ T cells (left panel) and CellROX^+^ γδ17 T cells (right panel) 5 h post-stimulation, cultured alone, in contact with WT or KO neutrophils (*n* = 4). Also see Supplementary Fig. 14E. **I** IL-17A release assessed by ELISA 16 h post-stimulation of γδ T cells, WT and KO neutrophils alone, γδ T cells co-cultured with WT or KO neutrophils, separated by transwell or pre-treated with anti-IL1β blocking antibody (*n* = 2). **J** IL-1β release of WT and KO neutrophils assessed by ELISA 16 h post-stimulation (*n* = 3). **K** *Il1b* mRNA expression of WT and KO neutrophils assessed by RT-qPCR 4 h post-stimulation (*n* = 3). **L** NFκB p65 DNA binding activity in lysates of WT and KO neutrophils 45 min post-stimulation. Positive control (PC) nuclear extract from Jurkat cells stimulated with TPA and calcium ionophore (*n* = 2). Bars represent mean ± SD of technical replicates representative of 2–4 independent experiments (**B**, **G**, **I**–**L**) or mean ± SD of 3–4 independent experiments (**E**, **H**). *p* values are indicated, *<0.05, **<0.01, ***<0.001 and ****<0.0001, ns not significant. *p* values were determined using the Kruskal–Wallis test with Dunn’s post-test (**B**), two-way ANOVA followed by Tukey’s post-test (**E**–**H**), and two-way ANOVA followed Sidak’s post-test (**I**–**L**). Source data are provided as a Source Data file.

To elucidate the Nox2-dependent interaction between γδ T cells and neutrophils, we conducted ex vivo experiments using cells isolated from mouse tissues. We obtained neutrophils via negative selection from bone marrow (BM), achieving a purity of 85.23 ± 9.86% neutrophils of live CD45^+^ cells (Supplementary Fig. 12A). Although isolated neutrophils do not support productive IAV infections, they respond to virus-associated molecular patterns via pattern recognition receptors and phagocytic mechanisms^40^. We confirmed assembly and activation of Nox2 in BM neutrophils upon stimulation with purified IAV by immunofluorescence staining (Fig. 4A). We observed translocation of cytoplasmic Nox2 subunit p47^phox^ (red) to the plasma membrane, colocalising with gp91^phox^ (green) starting 30 min post-stimulation. Colocalisation of both subunits was significantly increased at 60 min post-IAV-stimulation as measured by Pearson’s correlation (Fig. 4B).

We isolated γδ T cells from spleen and lymph nodes of wild-type mice (cell purity shown in Supplementary Fig. 12B) as well as from lung (cell purity shown in Supplementary Fig. 13) and co-cultured them with neutrophils from either wild-type or *Nox2*^*−/−*^ mice (experimental schematics Fig. 4C and Supplementary Fig. 14A). Direct cell contact was observed in co-cultures (Fig. 4D and Supplementary Fig. 14B), with a significant percentage of γδ T cells in contact with IAV-infected wild-type neutrophils staining CellROX^+^ (75 ± 36%) as compared to 1.7 ± 0.26% in contact with *Nox2*^*−/−*^ neutrophils (Fig. 4E and Supplementary Fig. 14C, D).

Infection of isolated γδ T cells with IAV did not change their IL-17A or IFNγ expression. However, co-culture with IAV-infected *Nox2*^*−/−*^ neutrophils significantly shifted γδ T cells towards IL-17A production (Fig. 4F, G and Supplementary Fig. 14E, F), resulting in a 10-fold increase in frequency of IL-17A-expressing γδ T cells (Fig. 4H, left panel and Supplementary Fig. 14G). Moreover, ROS production by wild-type neutrophils increased oxidative stress in γδ17 T cells, as indicated by a 5-fold increase in CellROX^+^ γδ T cells compared to those co-cultured with *Nox2*^*−/−*^ neutrophils (Fig. 4H, right panel).

In addition, the frequency of RORγt^+^ γδ T cells among total CD45^+^ cells increased, and we observed a higher proportion of IL-17A^+^ RORγt^+^ double-positive cells within the γδ T cell population following co-culture with *Nox2*^*−/−*^ neutrophils (Supplementary Fig. 15A–C), indicating expansion of IL-17-committed γδ T cells. In contrast, wild-type neutrophils insignificantly tended to induce IFNγ production in γδ T cells (Supplementary Fig. 15D, left panel), and Nox2 presence did not significantly impact oxidative stress in IFNγ-producing γδ T cells (Supplementary Fig. 15D, right panel). These findings align with Mensurado et al., who previously showed that CD27^*−*^ γδ17 T cells are more susceptible to oxidative stress due to low antioxidant gene expression^26^.

We further observed increased release of IL-17A in cell culture supernatant upon co-culturing with IAV-infected *Nox2*^*−/−*^ neutrophils measured by ELISA (Fig. 4I). Correspondingly, significantly elevated *Il17a* mRNA expression in IAV-infected γδ T cells co-cultured with *Nox2*^*−/−*^ neutrophils underlined a transcriptional regulation (Supplementary Fig. 15E). Of note, neutrophils alone did not produce significant levels of IL-17A.

Using transwell inserts to physically separate γδ T cells and neutrophils, we confirmed that suppression of IL-17A production by wild-type neutrophils was contact-dependent. In the absence of direct contact, co-culture with wild-type neutrophils led to increased IL-17A secretion from γδ T cells, comparable to levels induced by *Nox2*^*−/−*^ neutrophils (Fig. 4I). This suggests that ROS-mediated suppression of γδ T cells requires physical proximity due to local transfer of hydrogen peroxide from neutrophils to γδ T cells. This mechanism is supported by previous findings where neutrophil-derived ROS were shown to suppress neighbouring cells via contact-dependent delivery^23,24^.

Transwell experiments still showed higher induction of IL-17A release in co-culture with *Nox2*^*−/−*^ neutrophils compared to wild-type neutrophils, indicating the presence of a second, contact-independent activation mechanism. Since in vivo experiments showed elevated IL-1β production in lung neutrophils, we blocked IL-1β signalling in co-culture experiments by addition of a neutralising antibody and completely abrogated IL-17A release from γδ T cells (Fig. 4I).

We confirmed that *Nox2*^*−/−*^ neutrophils release more IL-1β protein and mRNA upon IAV infection compared to wild-type (Fig. 4J, K). Previous studies revealed a mechanism for Nox2-dependent regulation of neutrophil IL-1β production involving selective oxidation of the key transcription factor NFκB^20^, inhibiting its DNA-binding activity^18^. To examine if NFκB transcriptional activity was increased in Nox2-deficient neutrophils, we employed a transcription factor-binding assay that measures the binding of active p65 to its consensus sequence. In contrast to infected wild-type neutrophils, p65 activity was significantly upregulated in *Nox2*^*−/−*^ neutrophils (Fig. 4L).

Taken together, we identified two distinct Nox2-dependent mechanisms by which neutrophils modulate γδ T cell responses during viral infection: firstly, neutrophil-intrinsic redox regulation of NFκB activity reduces IL-1β production. Secondly, contact-dependent ROS transfer suppresses IL-17A expression in γδ T cells. While both wild-type and *Nox2*^*−/−*^ neutrophils activate γδ T cells via IL-1β, *Nox2*^*−/−*^ neutrophils, due to impaired ROS production, lack the suppressive proximity-dependent signal, thereby unmasking and amplifying the IL-1β-driven γδ T cell activation.

### Human neutrophils increase γδ17 T cells upon virus infection

A negative correlation between neutrophil and γδ T cell numbers has been noted in several human respiratory virus infections, suggesting an interaction between the two cell populations in humans as well. The ratio of immature neutrophils to γ(Vδ)2T cells has been proposed to predict severe COVID-19^41^. Further, in a human challenge study with RSV, it was observed that high mucosal neutrophil numbers before exposure would predict symptomatic RSV disease with presymptomatic decline in mucosal IL-17A^42^. We therefore explored whether our observations in mice could be extrapolated to human respiratory virus infections.

**Fig. 5:**
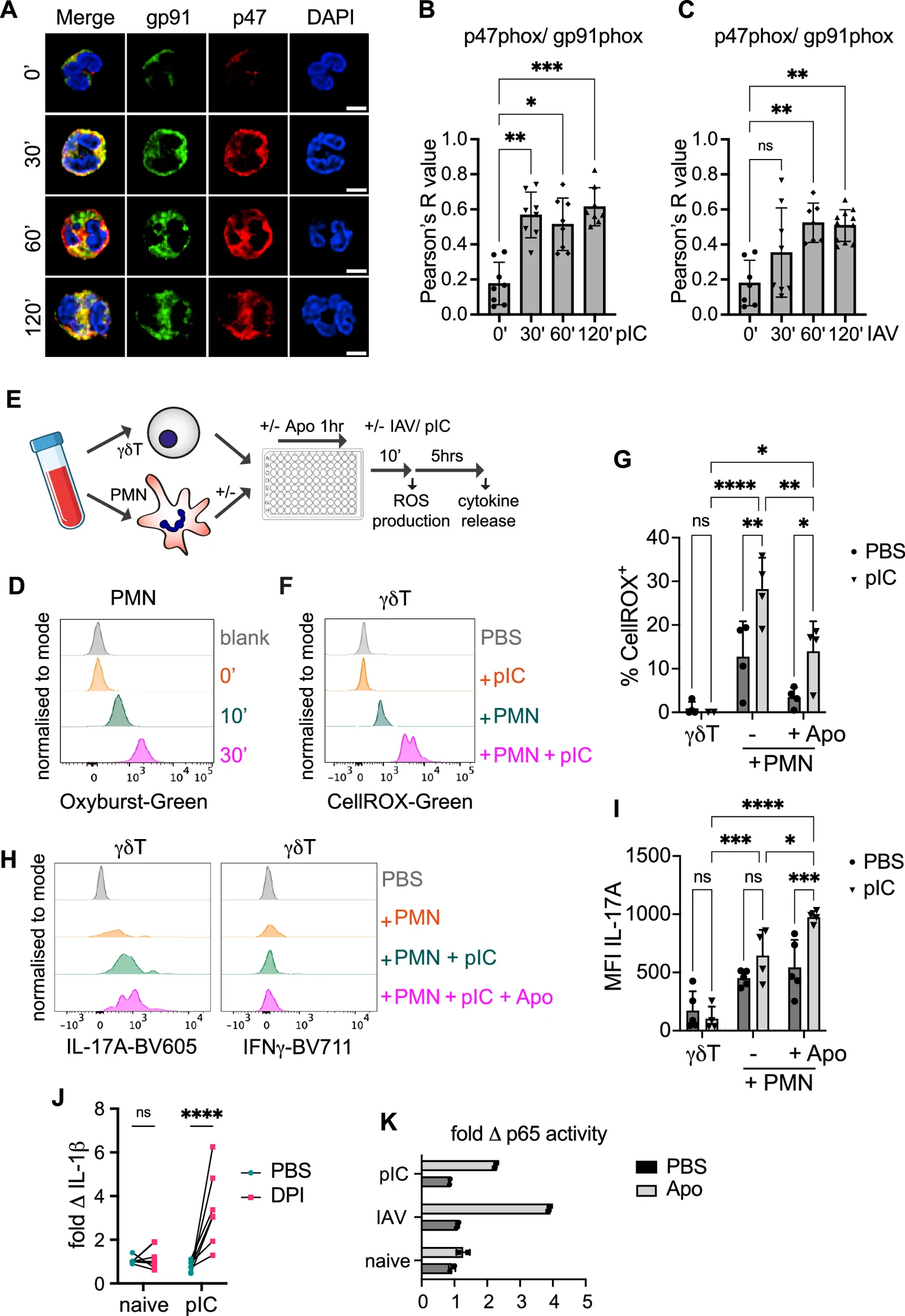
Human neutrophils enhance γδ17 T cells through Nox2-regulated IL-1β production during virus exposure. γδ T cells and neutrophils (PMN) isolated from peripheral blood of healthy volunteers were co-cultured ex vivo. Also see Supplementary Fig. 16A, D. **A** Representative immunofluorescence micrograph of isolated human neutrophils at indicated time points (minutes) post-treatment with 1 µg/ml complexed poly(I:C) (pIC) stained for p47^phox^ (red), gp91^phox^ (green) and nuclei (DAPI, blue). Scale bar 5 µm. **B**, **C** Pearson’s *R* values for colocalisation of p47^phox^ and gp91^phox^ in human neutrophils treated with 1 µg/ml pIC (**B**, *n* = 8) or stimulated with IAV PR8 at 0.2 MOI (**C**, 0′–60′: *n* = 7, 120’: *n* = 10) for indicated time points (minutes) per field of view. Also see Supplementary Fig. 16B. **D** Representative histogram plot of Oxyburst^+^ neutrophils stimulated for 0, 10 and 30 min with 1 µg/ml pIC gated as Live CD45^+^, CD11b^+^, CD16^+^ and CD14^*−*^. **E** Schematic of experimental setup. γδ T cells and neutrophils were isolated from the peripheral blood of healthy donors and co-cultured with or without the Nox2 inhibitor apocynin. After 1 h, cells were stimulated with IAV, pIC or mock-treated (PBS). ROS production (Oxyburst staining) was assessed 10–30 min post-stimulation; cytokine release in cell culture supernatant was measured 5 h post-stimulation. **F** Representative histogram plot of CellROX^+^ γδ T cells cultured alone, in co-culture (ratio 1:10) with autologous unstimulated or pIC-stimulated neutrophils for 5 h gated as Live CD45^+^, CD3^+^, CD4^*−*^ and γVδ2TCR^+^ cells. **G** Frequency of CellROX^+^ γδ T cells pre-treated 1 h with PBS or 300 µM apocynin (Apo) cultured alone, in co-culture with unstimulated or pIC-stimulated neutrophils for 5 h (γδ T alone: *n* = 3, co-culture: *n* = 4). **H** Representative histogram plots of IL-17A^+^ (left panel) and IFNγ^+^ (right panel) γδ T cells pre-treated 1 h with PBS or 300 µM apocynin (Apo) cultured alone, in co-culture with unstimulated or pIC-stimulated neutrophils for 5 h. **I** Geometric mean fluorescence intensity (MFI) of IL-17A^+^ γδ T cells pre-treated 1 h with PBS or 300 µM apocynin (Apo) cultured alone, in co-culture with unstimulated or pIC-stimulated neutrophils for 5 h (PBS: *n* = 5, pIC: *n* = 4). Also see Supplementary Fig. 16E, F. **J** IL-1β release of human neutrophils pre-treated 1 h with PBS or 10 µM diphenyleneiodonium (DPI) and stimulated with 1 µg/ml pIC for 5 h assessed by ELISA, calculated as fold change from untreated. Each data point represents one independent healthy donor. Values from the same donor are indicated (*n* = 6). **K** NFκB p65 DNA binding activity in human neutrophils pre-treated 1 h with PBS or 300 µM apocynin (Apo) and stimulated with 1 µg/ml pIC or IAV PR8 at 0.2 MOI for 45 min calculated as fold change from untreated (*n* = 2). Bars represent mean ± SD of technical replicates representative of 2–3 independent experiments (**B**, **C**, **K**) or mean ± SD of 3–6 independent experiments (**G**, **I**, **J**). *p* values are indicated, *<0.05, **<0.01, ***<0.001 and ****<0.0001, ns not significant. *p* values were determined using the Kruskal–Wallis test with Dunn’s post-test (**B**, **C**), two-way ANOVA with Tukey’s post-test (**G**, **I**, **K**), and two-way ANOVA with Sidak’s post-test (**J**). Source data are provided as a Source Data file.

Firstly, we confirmed that viral recognition induces ROS production via Nox2 activation in human neutrophils. We isolated neutrophils from the peripheral blood of healthy donors using centrifugation over a discontinuous density gradient according to a previously established protocol^43^ and assessed purity by flow cytometry. On average 99.1 ± 0.4% of Live CD45^+^ gated cells were neutrophils defined as CD11b^+^, CD14^*−*^, CD16^+^, CD66b^+^, CCR3^*−*^ and Siglec-8^*−*^ cells, confirming minimal contamination by eosinophils (Supplementary Fig. 16A). Nox2 assembly was observed by immunofluorescence staining for subunits p47^phox^ (red) and gp91^phox^ (green) 30 min post-stimulation with synthetic double-stranded RNA virus analogue poly(I:C) (polyinosinic-polycytidylic acid, Fig. 5A), or 1-h post-stimulation with IAV (Supplementary Fig. 16B). Transient colocalisation of subunits p47^phox^ and gp91^phox^ was confirmed by a significant rise in Pearson’s *R* value (Fig. 5B, C). Additionally, we measured production of hydrogen peroxide with Oxyburst reagent in poly(I:C)-stimulated human neutrophils after 10 and 30 min (Fig. 5D). We further explored the ROS burst induced by human neutrophils in response to virus detection with two chemiluminescent probes: luminol, which is cell-permeable and detects both intracellular and extracellular ROS, and isoluminol, which detects only extracellular ROS. Poly(I:C) elicited a potent ROS burst within 20–60 min, peaking at 30 min; however, very little extracellular ROS was measured (Supplementary Fig. 16C). The initial delay in ROS detection is consistent with ROS being consumed intracellularly.

Next, we isolated human γδ T cells from the peripheral blood of healthy donors. On average, 78 ± 11.9% of live CD45^+^ T cells were γδ2 T cells (Supplementary Fig. 16D). γδ2 T cells were co-cultured with autologous neutrophils that were pre-treated with either PBS or Nox2 inhibitor apocynin prior to stimulation with poly(I:C) or IAV (Experimental schematic Fig. 5E).

Poly(I:C) stimulation alone did not induce oxidative stress in γδ2 T cells as indicated by CellROX^+^ staining. However, poly(I:C)-stimulated neutrophils significantly upregulated CellROX^+^ γδ2 T cells, indicating that neutrophils actively transferred ROS to γδ2 T cells (Fig. 5F, G). This was reversed by pre-treating neutrophils with apocynin, confirming the role of Nox2-derived ROS.

Flow cytometry analysis of neutrophil-γδ2 T cell co-cultures showed that poly(I:C) alone did not increase IL-17A or IFNγ production in γδ2 T cells (Fig. 5H, I and Supplementary Fig. 16E, F). However, co-culture with poly(I:C)-stimulated neutrophils significantly induced IL-17A production. Moreover, blocking ROS production in poly(I:C)-stimulated neutrophils with apocynin pre-treatment significantly increased IL-17A^+^ γδ2 T cells. This suggests that human neutrophils, like mouse neutrophils, suppress IL-17A production in γδ T cells via Nox2-derived ROS. Consistently, direct treatment of isolated γδ2 T cells with hydrogen peroxide abolished IL-17A expression, whereas scavenging ROS through addition of superoxide dismutase and catalase increased IL-17A release from IAV-stimulated γδ2 T cells (Supplementary Fig. 16G, H).

In parallel, we found that Nox2 inhibition in poly(I:C)-stimulated neutrophils resulted in increased IL-1β secretion (Fig. 5J) and a significant upregulation of NF-κB p65 transcriptional activity (Fig. 5K).

Our results suggest that the interplay between neutrophils and γδ T cells in regulating inflammatory cytokine signalling is a conserved mechanism observed in both mice and humans. Neutrophil Nox2 regulates γδ T cell function by two distinct yet complementary mechanisms: (1) suppression of IL-1β production via redox control of NF-κB activity in neutrophils and (2) contact-dependent inhibition of γδ T cell IL-17A production through ROS transfer.

### Early Nox2-deficient neutrophil-derived IL-1β induces protective γδ17 T cell response in mice

**Fig. 6:**
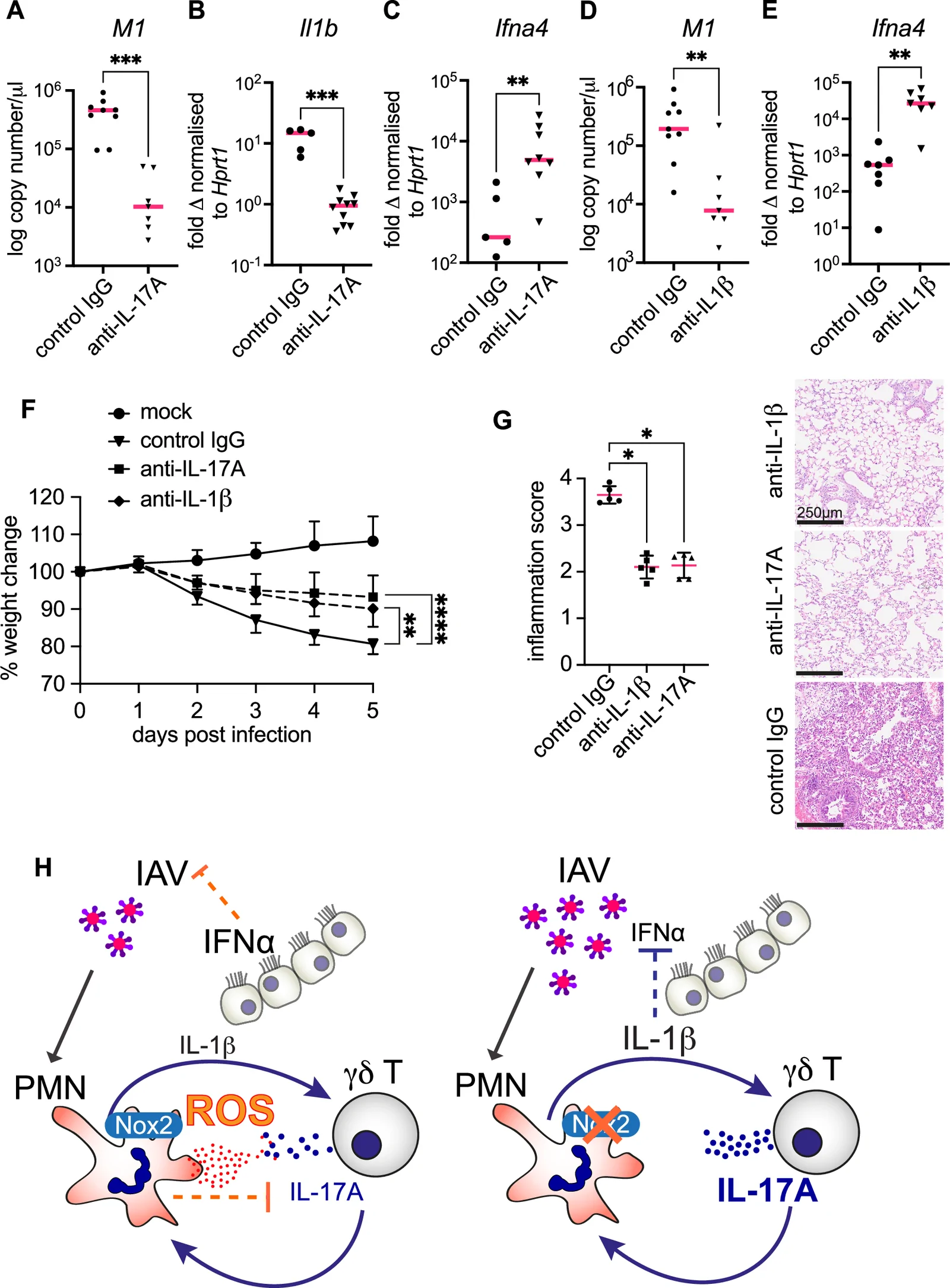
Early IL-1β production from Nox2-deficient neutrophils induces γδ17 T cell response and inhibits protective antiviral interferon response. Male and female *Ly6G-Cre*^+/*−*^ *Nox2*^*fl/fl*^ mice were infected with 3 × 10^4^ TCID_50_ IAV strain X-31 or PBS (mock) in 30 µl intranasally for 3 days. **A**–**C** Mice were treated with 100 µg anti-IL17A or control IgG antibody in 200 µl intraperitoneally at 1- and 2-days post-infection. Virus burden was assessed by RT-qPCR of influenza matrix *M1* RNA in whole lungs (**A**, control IgG: *n* = 9, anti-IL17A: *n* = 7). *Il1b* (**B**) and *Ifnα4* (**C**) mRNA expression in whole lungs was quantified by RT-qPCR (control IgG: *n* = 5, anti-IL17A: *n* = 10 *Il1b* and *n* = 8 *Ifna4*). **D**, **E** Mice were treated with 100 µg anti-IL1β or control IgG antibody in 200 µl intraperitoneally at 1- and 2-days post-infection. Virus burden was assessed by RT-qPCR of influenza matrix *M1* RNA in whole lungs (**D**, control IgG: *n* = 9, anti-IL1β: *n* = 7). *Ifnα4* mRNA expression in whole lung was quantified by RT-qPCR (**E**, *n* = 7). **F** Mice were treated with 100 µg anti-IL1β, anti-IL17A or control IgG antibody in 200 µl intraperitoneally at 1-, 2-, 3- and 4-days post-infection. Weight loss was monitored over 5 days (*n* = 5). **G** Mice were treated with 100 µg anti-IL1β, anti-IL17A or control IgG antibody in 200 µl intraperitoneally at 1-, 2-, 3- and 4-days post-infection. Inflammation was graded as follows: 0, normal; 1, mild; 2, moderate; 3, severe; and 4, very severe inflammation (Left panel, *n* = 5). Representative micrographs of H&E-stained lung sections at 5 days post-infection. Scale bar 250 µm (right panel). **H** Proposed model of neutrophil-γδ T cell interaction during early IAV infection. In wild-type mice, IAV infection triggers neutrophil Nox2 activation, leading to the production of ROS. Nox2-derived ROS suppress IL-1β signalling from neutrophils and, in turn, limit proliferation of IL-17-producing γδ T cells (left panel). In the absence of Nox2, this regulatory mechanism is disrupted, and amplified IL-1β/IL-17 signalling weakens the antiviral type I IFN response, impairing virus clearance (right panel). Bars represent mean ± SD of 2–3 independent experiments. Each data point represents one animal (**A**–**E**, **G**). *p* values are indicated, *<0.05, **<0.01 and ***<0.001, ns not significant. *p* values were determined using a two-tailed Mann–Whitney test (**A**–**E**), two-way ANOVA followed by Sidak’s post-test (**F**), or a Kruskal–Wallis test with Dunn’s post-test (**G**). Source data are provided as a Source Data file.

Ultimately, we investigated the impact of neutrophil Nox2-mediated suppression of γδ17 T cells on the outcome of IAV infection in mice. We blocked IL-17A signalling by injecting an anti-IL-17A neutralising antibody and compared virus burden 3 days post-infection to that of control IgG antibody-injected *Ly6G-Cre*^*+/−*^ *Nox2*^*fl/fl*^ mice. We observed a significant decrease in virus burden in IL-17A-depleted animals (Fig. 6A), along with downregulation of *Il1b* mRNA transcripts in the lungs (Fig. 6B) and elevated levels of antiviral *Ifna4* (Fig. 6C), indicating restoration of the antiviral immune response.

Moreover, we reversed the viral burden by neutralising IL-1β signalling (Fig. 6D), which also correlated with increased *Ifna4* production (Fig. 6E). In addition, mice receiving anti-IL-1β or anti-IL-17A treatment experienced reduced weight loss (Fig. 6F) and showed decreased lung inflammation as assessed by histological scoring (Fig. 6G), consistent with improved disease outcomes. In conclusion, neutrophil Nox2 plays a crucial role in controlling inflammation during virus infection and maintaining an effective antiviral immune response.

## Discussion

Our work identifies neutrophil Nox2-derived reactive oxygen species as critical regulators of the immune response during the early stages of IAV infection. While traditionally regarded as tissue-damaging molecules, our data reveal that neutrophil Nox2-generated ROS play a pivotal role in restraining excessive inflammation, promoting balanced cytokine responses and supporting effective antiviral immunity.

We demonstrate that neutrophils are a predominant early source of ROS during IAV infection, and that intracellular Nox2-derived ROS suppress IL-1β production, thereby limiting the expansion of IL-17-producing γδ T cells. In the absence of Nox2, unchecked IL-1β production by neutrophils leads to γδ T cell proliferation and IL-17 secretion, establishing a feed-forward IL-1β/IL-17 loop. This exacerbation interfered with the antiviral type I IFN response, resulting in diminished viral clearance and heightened lung inflammation. Importantly, this feedback circuit was also recapitulated in human cells stimulated with viral analogues, highlighting its translational relevance.

Our findings extend previous work showing that ROS modulate immune responses in a context-dependent manner, particularly influenced by pathogen size and localisation. During bacterial and fungal infections, phagocytosis of small microbes triggers intracellular ROS, which suppress IL-1β through redox modification of NF-κB, while extracellular ROS induced by large microbes promote IL-1β expression and neutrophil recruitment^20^. We show that viral sensing by neutrophils induces rapid intracellular ROS production within 30 min via Nox2 complex assembly, suppressing NF-κB activity and IL-1β expression. This suppression is reversed by Nox2 inhibition, confirming ROS-dependent control. ROS-dependent regulation of NFκB binding to DNA has been demonstrated previously^18^. However, we cannot rule out the involvement of redox-sensitive caspase-1 in regulating cleavage of pro-IL-1β into active IL-1β, which remains a potential area for further exploration^19^.

In *Ly6G-Cre*^*+/−*^ *Nox2*^*fl/fl*^ mice, elevated IL-1β promotes excessive neutrophil recruitment and swarming to sites of infection and enhances IL-17A production by γδ T cells. Ultimately, this impaired virus clearance and caused lung damage, indicating that ROS act as gatekeepers to prevent inflammation from undermining antiviral immunity.

A caveat is the cellular specificity of Ly6G-Cre targeting. While Ly6G-Cre is widely used to manipulate mature neutrophils, recent work has identified a recruited Ly6G-expressing macrophage population in the lung under inflammatory conditions^32^. We therefore cannot fully exclude contributions from Ly6G^+^ macrophages to phenotypes observed in conditional Nox2 deletion. Importantly, in our dataset this Ly6G^+^ macrophage population remained small during the early window analysed here (days 1–3 post-infection).

Beyond γδ T cells, Nox2-deficient neutrophils alter the broader myeloid landscape. We observed a significant rise in inflammatory Ly6C^hi^ monocytes and macrophages at day 3 post-infection, potentially fuelling inflammation. Moreover, surface marker changes on neutrophils of *Ly6G-Cre*^*+/−*^ *Nox2*^*fl/fl*^ mice revealed increased degranulation and tissue retention, suggesting that neutrophils have potentially enhanced their intrinsic capacity to drive inflammation, consistent with a more inflammatory phenotype.

Mechanistically, our data show that neutrophil-derived IL-1β promotes the proliferation of IL-17-producing γδ T cells while Nox2-generated ROS act as a counterbalance, suppressing this pathway. Secondly, direct cell contact between neutrophils and γδ T cells increased Nox2-dependent oxidative stress in γδ T cells, influencing their function and survival.

Our findings align with previous research showing that neutrophil-derived ROS suppress γδ17 T cell function. In fungal and bacterial lung infection models, neutrophilic NLRP3 inflammasome-dependent IL-1β secretion regulates γδ17 T cell responses^36^, and neutrophil depletion increased IL-17A production by γδ T cells^44^. Excessive ROS production in superoxide dismutase 3 (SOD3)-deficient mice led to early neutrophil apoptosis, which in turn reduced IL-1β levels and limited γδ T cell proliferation^45^. Catalase addition to co-cultures of human blood-isolated neutrophils and γδ T cells to break down hydrogen peroxide reversed γδ T cell suppression^46^. Interestingly, nitrogen-containing bisphosphonates (N-BP) are taken up by neutrophils and turned into isopentenyl pyrophosphate (IPP), which is known to activate γδ T cells. However, while N-BP-stimulated monocytes drive γδ T cell proliferation, neutrophils inhibited their activation, acting as a regulatory checkpoint^25^. The same authors suggested the release of hydrogen peroxide and serine proteases by neutrophils as inhibiting factors^25^.

Further studies have focussed on the role of neutrophil ROS production in γδ T cell inhibition. In various sterile and infectious models, including the tumour microenvironment, bacterial lung infections and the autoimmune skin disease psoriasis, neutrophil-induced oxidative stress has been implicated in the suppression of γδ T cell activity^26,27,45^. Neutrophil-mediated intercellular transfer of ROS has previously been demonstrated to modulate the function of various cell types, including human CD8^+^ T cells^23^, murine megakaryocytes^24^ and γδ T cells^26^. Mechanistically, Mensurado et al. demonstrated that low expression of the antioxidant glutathione in CD27^−^ γδ17 T cells rendered them particularly susceptible to neutrophil-derived ROS^26^, explaining the shift from IFNγ to IL-17A production in our model upon IAV infection of *Ly6G-Cre*^*+/−*^ *Nox2*^*fl/fl*^ mice.

γδ T cells are functionally diverse and can be broadly divided into two major subsets based on CD27 expression: CD27^+^ γδ T cells, which typically produce IFNγ and are associated with cytotoxic and pro-inflammatory responses; and CD27^−^ γδ T cells, which preferentially produce IL-17A and are implicated in early antimicrobial defence and inflammatory amplification. These subsets arise from distinct developmental pathways and exhibit differential metabolic and transcriptional profiles.

Our results reveal a significant impact of neutrophil-derived ROS on γδ T cell metabolic programming and activation status. Specifically, we observed a marked increase in CD27^−^ γδ T cells and a reduction in CD27^+^ γδ T cells in the lungs of IAV-infected *Ly6G-Cre*^*+/−*^ *Nox2*^*fl/fl*^ mice. While CD27^+^ γδ T cells showed diminished chemotactic potential, CD27^−^ γδ T cells exhibited increased expression of markers indicative of heightened metabolic activity and proliferation.

We cannot exclude an additional role for neutrophil-released serine proteases, such as neutrophil elastase, in γδ T cell inhibition. In in vitro culture systems, neutrophil elastase has been shown to suppress the proliferation of blood-derived Vγ9δ2 T cells and to modulate their cytokine production^47,48^. While we focused on the Nox2-ROS axis, other redox-regulated pathways may contribute to the overall immune response during viral infections. Future studies could investigate how ROS interact with various neutrophil-secreted factors to modulate γδ T cell activity and antiviral immunity.

Interestingly, in our model, increased γδ T cell-derived IL-17 in the absence of neutrophil Nox2 coincided with amplified viral burden, inflammation and lung pathology. However, the role of IL-17 in respiratory infections is context-dependent: while early IL-17 signalling in the upper airway can aid pathogen clearance and tissue repair^49,50^, excessive or prolonged IL-17 responses may compromise barrier function and amplify inflammation^51,52^. Carissimo et al. showed an elevated neutrophil-to-Vδ2 T cell ratio correlating with severe COVID-19, suggesting that increased numbers of neutrophils could cause γδ T cell deficiency in patients contributing to the dysregulated immune response^41^. A human challenge study with RSV found that neutrophilic inflammation in the nasal mucosa predisposes to RSV infection, which was linked to a decline of mucosal IL-17^42^. This highlights the differing effects of IL-17 in the upper —especially nasal mucosa—versus lower respiratory tract, raising the question of when IL-17 becomes detrimental during IAV infection by impairing lung barrier function. The interplay between neutrophils and γδ T cells is crucial in determining the balance between effective antiviral responses and excessive inflammation, underscoring the potential for targeted therapeutic interventions to modulate these interactions.

Furthermore, proinflammatory IL-1β/IL-17 signalling can downregulate the antiviral type I IFN response. Previous studies have shown that IL-1β-driven upregulation of PGE2 inhibits type I IFN production during IAV infection in mice. When PGE2 receptors on macrophages are activated, they trigger the PI3K-Akt pathway, which prevents the phosphorylation of IRF3, a crucial transcription factor for type I IFN production. This suppression leads to increased viral replication and a weakened antiviral immune response^35,53^. Elevated PGE2 levels, correlating with increased IL-1β signalling and viral load, were also observed in IAV-infected *Ly6G-Cre*^*+/−*^ *Nox2*^*fl/fl*^ mice.

In addition to γδ T cell skewing, our results reveal a transient expansion of double-negative (CD4^−^CD8^−^) αβ T cells (DN T cells) at day 3 post-infection in the lungs of *Ly6G-Cre*^*+/−*^ *Nox2*^*fl/fl*^ mice, coinciding with a reduction in CD4^+^ and CD8^+^ αβ T cells. These DN T cells display an inert phenotype, lacking IL-17A and IFNγ production and showing reduced activation marker expression. In contrast to γδ T cells, DN T cells were not directly affected by ROS-induced oxidative stress. These observations suggest that neutrophil ROS may be required for appropriate αβ T cell differentiation and effector programming early during infection. Whether DN T cells represent a developmental stall or are diverted from CD4^+^/CD8^+^ fates remains unclear.

Our study highlights the dual nature of ROS in viral infections. While excessive ROS can damage host tissues, tightly regulated Nox2-derived ROS from neutrophils act as immunomodulatory signals that limit cytokine-driven inflammation and preserve antiviral immunity. This positions Nox2-derived ROS as promising therapeutic targets. However, effective modulation requires precise understanding of their cellular source and localisation. Neutrophil-derived ROS differ functionally from ROS produced by other cells, with their localisation being key to regulating IL-1β and IL-17 signalling. Therefore, therapies should focus on targeted regulation rather than broad-spectrum approaches.

Broad-spectrum antioxidants may lack efficacy because they fail to discriminate between beneficial and harmful ROS. Instead, spatial and cell-specific targeting—for example, limiting antioxidants to the airway epithelium—could mitigate epithelial damage without compromising neutrophil function.

Our results are consistent with observations made in chronic granulomatous disease (CGD) patients, who have genetic defects in Nox2 expression. CGD patients experience recurring bacterial and fungal infections and are also prone to develop chronic inflammation and autoimmune symptoms^54,55^. Moreover, an expansion of Th17 cells has been particularly noted in CGD patients, further contributing to immune dysregulation^56,57^.

Supporting the importance of cell-specific ROS regulation, studies using full Nox2 KO mice showed a modest improvement in the resolution of IAV infection, but with increased inflammation^15,16^. These findings suggest that the absence of Nox2 enhances viral clearance at the expense of heightened inflammatory responses due to the loss of ROS-mediated regulation across all cell types. In contrast, our study using neutrophil-specific Nox2 KO mice reveals that neutrophil-derived ROS specifically regulate IL-1β and IL-17 signalling, playing a critical role in maintaining a balanced inflammatory response while preserving the antiviral functions of other immune cells.

A limitation of our study is the complexity of translating findings from murine models to human disease. Although we observed similar mechanisms in human cells exposed to viral analogues, clinical studies are needed to validate the therapeutic potential of targeting Nox2-derived ROS in humans. Furthermore, the context-dependent role of IL-17, which can be both protective and detrimental depending on the site of inflammation (upper versus lower respiratory tract), warrants further investigation to delineate its precise role in respiratory viral infections.

Our findings have significant implications for chronic respiratory diseases like asthma and chronic obstructive pulmonary disease (COPD), where oxidative stress and inflammation play key roles. The dual role of ROS suggests that modulating ROS levels through targeted interventions could help balance inflammation and enhance antiviral immunity, potentially reducing exacerbations in asthma and COPD patients. Recognising the beneficial role of ROS in immune regulation supports the concept of 'good oxidants'^58^, where controlled ROS production is essential for maintaining immune homeostasis. Over-suppression of ROS might hinder essential immune functions, highlighting the need for targeted antioxidant therapies.

## Methods

### Mice

All animal experiments were approved by the King’s College London Animal Welfare and Ethical Review Body (AWERB) and conducted under a UK Home Office Project Licence (PP2442888) in accordance with the Animals (Scientific Procedures) Act 1986.

All mice strains used were derived from the C57Bl/6J background. Wild-type (WT) mice were purchased from The Jackson Laboratory. *Nox2*^*−/−*^ mice were kindly provided by Venizelos Papayannopoulos (Francis Crick Institute) and described previously^59^, *Nox2*^*fl/fl*^ mice by Ajay Shah (King’s College London)^60^ and *Ly6G-Cre* tdTomato mice by Matthias Gunzer (University Duisburg-Essen)^28^. *Ly6G-Cre* and *Nox2*^*fl/fl*^ mice were crossed to generate *Ly6G-Cre*^*+/−*^ *Nox2*^*fl/fl*^ and *Ly6G-Cre*^*−/−*^ *Nox2*^*fl/fl*^ mice.

Mice were maintained under specific pathogen-free conditions in the King’s College London Biological Services Unit on a 12-h light-dark cycle at an ambient temperature of 20–24 °C and relative humidity of 45–65%, with free access to food and water and environmental enrichment. Animals were 8–14 weeks old and age-matched within 1–2 weeks for experiments. Littermates of both sexes were randomly assigned to experimental groups. Control animals were cohoused. A full list of animals used for each experiment is provided in the Source data file.

### Human volunteers

Human participants were recruited under a protocol sponsored by King’s College London (co-sponsored by Guy’s and St Thomas’ NHS Foundation Trust) that was approved by the London Bridge Research Ethics Committee (REC reference 14/LO/1699; IRAS Project ID 147574).

All participants provided written informed consent prior to enrolment. Healthy adult donors had no history of respiratory disease and met the study inclusion and exclusion criteria. Recruitment was based on voluntary participation and therefore may be subject to self-selection bias, whereby individuals willing to participate in research may differ from the general population in health status or other unmeasured characteristics. A total of 22 donors were recruited (13 female, 9 male), aged 22–61 years (mean ± SD: 35 ± 12 years). A list of donors is provided in the source data file.

### Viruses

Influenza A virus strains X-31^61^ and PR8^62^ were kindly provided by John McCauley (Francis Crick Institute). SARS-CoV2 England 02 strain was kindly provided by Rui Pedro Ribeiro Galao (King’s College London)^63^.

### Virus purification and titration

Influenza A virus strains were grown in chicken eggs, filtered and titrated. TCID_50_ was determined using Madin-Darby Canine Kidney (MDCK, Sigma) cells. MDCK cells were seeded in 96-well plates in Dulbecco’s Modified Eagle Medium (DMEM, Gibco) supplemented with 10% fetal calf serum (FCS, Hyclone), 100 U/mL penicillin and 100 μg/mL streptomycin. At 80–90% confluency, plates were washed with phosphate-buffered saline (PBS), and 8-fold serially diluted virus was added in DMEM supplemented with 0.1% FCS, 0.3% bovine serum albumin (BSA, Thermo Fisher), 100 U/mL penicillin and 100 μg/mL streptomycin, 200 μM GlutaMAX, 20 mM HEPES (both from Gibco) and 1 µg/ml L-1-tosylamido-2-phenylethyl chloromethyl ketone (TPCK)-treated trypsin (2BScientific). Cells were fixed in 4% paraformaldehyde (PFA, VWR) after 5 days and stained with crystal violet (Sigma). Plates were analysed for cytopathic effect and TCID_50_ calculated using Reed and Muench methodology^64^.

Influenza A virus was purified as described previously^65^. Briefly, MDCK cells grown in T175 flasks were inoculated at multiplicity of infection (MOI) 0.001 in 6 ml serum-free Minimum Essential Medium (MEM, Gibco). Cells were incubated for 1 h and 25 ml serum-free MEM was added per flask and incubated for 3 days. Supernatant was filtered and layered above 5 ml of 30% sucrose (Sigma). Ultracentrifugation was carried out at 100,000 × *g* (acceleration 9, deceleration 0) for 90 min at 4 °C. Supernatant was removed, and virus was resuspended in 3 ml DMEM.

### Infection of mice and treatments

Eight to 14-week-old mice were anesthetised with isoflurane and infected intranasally with 3 × 10^4^ TCID_50_ IAV X-31 in a volume of 30 µl. Body weight and health status were monitored daily. Whole lungs or bronchoalveolar lavage (BAL), blood, spleen and lymph nodes were collected at various time points post-infection.

For cytokine neutralisation, 100 µg anti-IL-1β, anti-IL-17A antibody or the corresponding isotype control was administered intraperitoneally in a volume of 200 µl at day 1 and day 2 post-infection. For neutrophil depletion, 150 µg anti-Ly6G or corresponding isotype control antibody (all antibodies BioXcell) were administered intraperitoneally in a volume of 200 µl at −1 day, −1 h prior, and +1 day post-infection.

Mice were euthanised if they lost more than 15% of their initial body weight sustained for more than 48 h in conjunction with additional clinical signs, or more than 20% body weight irrespective of other signs. Additional humane endpoints included continuous or recurrent hunched posture (over 24 h), reduced food or water intake to less than 50% of normal (over 48 h), subdued behaviour or isolation from peers (over 24 h), diarrhoea lasting more than 48 h or changes in fur (piloerection over 48 h). Animals were euthanised by cervical dislocation, except when bronchoalveolar lavage or lung perfusion was performed, in which case euthanasia was by pentobarbital overdose.

### Viral load assessment

Virus was quantified in infected lungs by qPCR for the Influenza A Matrix gene M1. Whole lungs were collected in RNAlater solution (Invitrogen), and RNA was isolated using TriReagent-Chloroform extraction followed by isopropanol precipitation (all chemicals from Sigma-Aldrich). The TaqMan RNA-to-CT *1-Step* Kit (Applied Biosystems) in combination with the PCRmax Human Influenza A virus M1 kit (Cole Parmer) was used to quantify M1 copy number.

### Histology

Whole lungs were harvested at various time points post-infection, fixed overnight in 4% paraformaldehyde (PFA, VWR), embedded in paraffin, sectioned and stained with hematoxylin and eosin. Images were acquired using the NanoZoomer slide scanner system (Hamamatsu). Histopathological analysis was performed, blinded to groupings and genotype for various parameters as follows: bronchiolitis classified as damage to airway epithelial cells, necrotic bodies, or denudation of airway epithelial lining; alveolitis classified as damaged alveolar epithelial cells or denuded epithelial lining; bronchiole inflammation classified as bronchioles filled with inflammatory cells; haemorrhage classified as presence of erythrocytes in the alveolar airspace, damaged capillaries and haemorrhagic effusions in the damaged areas; interstitial inflammation classified as inflammation in the alveoli or thickening of the alveolar interstitium; endothelial damage classified as necrotic endothelium present within small blood vessels or capillaries. Damage severity was scored on a 4-point scale: 0 indicates none or very minor; 1 indicates mild; 2 indicates intermediate; 3 indicates moderately severe and 4 indicates severe and widespread.

### BAL analysis

Bronchoalveolar lavage (BAL) of the whole lung was performed with 0.5 mL saline via a tracheal cannula. Samples were centrifuged at 300 × *g* for 10 min at 4 °C, and supernatant was analysed for IFNγ, IL-17A, IL-17F, IL-1β, IL-6, KC, MCP-1, TNF using Cytometric bead array (CBA, BD Bioscience) as well as proteome profiler mouse XL cytokine array (R&D) according to the manufacturer’s instructions. Sandwich ELISA kits were used for the detection of mouse IL-1β, IL-17A, IFNα4 (all Invitrogen), IFNβ (PBL), IFNλ (Biotechne), PGE2 (LSBio) and TNF (Stemcell).

### Cell culture

Mouse neutrophils were isolated from the bone marrow of C57Bl/6J and *Nox2*^*−/−*^ mice using the EasySep Mouse Neutrophil Enrichment kit (Stemcell Technologies) according to the manufacturer’s instructions. Mouse γδ T cells were isolated from lymphoid tissues (spleen and lymph nodes) or lungs harvested from C57Bl/6J mice using the EasySep Release Mouse APC Positive Selection kit (Stemcell Technologies) and 2 µg/ml anti-γδTCR antibody (GL3, Biolegend).

Human neutrophils and peripheral blood mononuclear cells (PBMC) layers were freshly isolated over Histopaque-1119 (Merck) gradient followed by a discontinuous Percoll (GE Healthcare) gradient for neutrophil isolation as described previously^43^. Human γδ T cells were isolated from PBMCs using the EasySep human γδ T cell Isolation kit (Stemcell Technologies) according to the manufacturer’s instructions.

Mouse and human neutrophils alone or in co-culture with γδ T cells were plated in Hank’s balanced salt solution plus Ca^2+^ and Mg^2+^ (Gibco) supplemented with 3% human plasma or 10% fetal calf serum (FCS, Hyclone) for mouse neutrophils, respectively. Cells were pretreated for 1 h with 10 µM NADPH oxidase inhibitor diphenyleneiodonium (DPI, Cambridge Bioscience), 300 µM Nox2 inhibitor apocynin (Merck), 700 U/ml superoxide dismutase (Merck) in combination with 5000 U/ml catalase (Merck) or 500 µM hydrogen peroxide. Mouse and human cultures were stimulated with IAV strain PR8 at MOI 0.2 or 1 µg/ml poly(I:C)(Invivo Gen).

### Flow cytometry

Mouse tissues were processed into single-cell suspensions by passing through 70 µm cell strainers. Lung tissue was digested in 0.4 mg/ml Liberase TL (Roche) for 45 min at 37 °C with shaking. After erythrocyte lysis with ammonium-chloride-potassium (ACK, Gibco) buffer, cells were washed, filtered, and resuspended in Iscove’s modified Dulbecco’s medium (IMDM, Gibco) supplemented with 10% FCS (Hyclone).

For cell surface staining, 1 × 10^6^ cells per sample were incubated with 2.5–5 µg/ml antibodies for 15 min at room temperature, washed and fixed in fixation buffer (Biolegend) for 20 min at 4 °C.

For intracellular cytokine staining, 2 × 10^6^ cells were incubated at 37 °C for 5 h with 1× cell activation cocktail (Biolegend). Protein transport blockers 1× Brefeldin and 1× Monensin (both Biolegend) were added for the last 3 h of incubation. Cells were fixed and permeabilised in BD Cytoperm/Cytofix buffer (BD Bioscience) for 20 min at 4 °C, blocked for 10 min with 10 µg/mL TruStain FcX (Biolegend), and stained with 5–10 µg/ml antibody for 1 h at room temperature in the dark.

Dead cells were excluded with the LIVE/DEAD Fixable Aqua Dead Cell Stain Kit (Invitrogen). Cells were acquired on a Fortessa or Cytoflex cell analyser (BD Biosciences), sorted on FACS Aria and data analysed using FlowJo v10.9 software (BD Biosciences).

### Immunofluorescence staining and confocal microscopy of fixed cells and tissue

Mouse lungs were collected 1, 2, 3 and 5 days post-infection, fixed overnight in 4% paraformaldehyde (VWR), and dehydrated in 20% sucrose (Sigma) for 72 h. For γδTCR immunostaining, lungs were fixed by inflation and immersion in 1% paraformaldehyde in PBS for 2 h^66^. After embedding in OCT (Optimum Cutting Temperature Compound, VWR), lungs were frozen on dry ice and sectioned at mid-levels on a Leica CM3050 S Cryostat.

1 × 10^5^ human or mouse neutrophils were plated on poly-D-lysine-coated (Corning) glass coverslips 1 h before stimulation. After various time points, cells were fixed in 2% Image-iT fixative solution (Invitrogen) for 20 min at 37 °C and washed 3 times in PBS.

Fixed cells and lung sections were permeabilised with 0.5% Triton-X 100 in PBS for 2 min, blocked with animal-free blocking diluent (2B Scientific) for 1 h and incubated with anti-IAV nucleoprotein (Invitrogen), anti-mouse Ly6G-Alexa Fluor 647 (clone 1A8), anti-hamster γδTCR (both Biolegend), or anti-human/mouse p47^phox^ (Santa Cruz), anti-gp91^phox^-Alexa Fluor 488 (SantaCruz, for detection in isolated cells), anti-gp91^phox^ (GeneTex, for detection in tissue sections) antibodies. Primary antibodies were detected with Alexa Fluor-conjugated secondary antibodies (Invitrogen). Cells and tissue sections were mounted in ProLong Diamond containing 4′,6-diamidino-2-phenylindole (DAPI, Invitrogen) and imaged with a Nikon A1R confocal microscope. Images were analysed with *Fiji* software (ImageJ version 1.54p, NIH).

For quantification of NP and Ly6G staining, immunofluorescence images were acquired using identical microscope settings and analysed using *Fiji*. Representative regions of interest (ROIs) were selected for each tissue section, and the mean fluorescence intensity within each ROI was measured using the *Measure* function following background subtraction and thresholding. Mean intensity values were averaged across multiple ROIs per sample. All image analysis was performed blinded to experimental condition.

Colocalisation of p47^phox^/gp91^phox^ was assessed using a Python (v3.9) script in JupyterLab (v3.6.7) based on calculation of Pearson’s correlation coefficient available on GitHub (Repository: AnniWarn/Nox2)^67^.

To verify neutrophil-specific deletion of Nox2 by immunofluorescence staining, detection of gp91^phox^ together with tdTomato-expressing neutrophils was quantitated in frozen lung sections from *Ly6G-Cre*^*+/−*^ and *Ly6G-Cre*^*+/−*^ *Nox2*^*fl/fl*^ mice using CellProfiler (version 4.2.8). Briefly, nuclei were identified from the DAPI channel using the IdentifyPrimaryObjects module, and tdTomato^+^ neutrophils were segmented from the tdTomato channel. Nox2 fluorescence intensity was quantified within individual tdTomato^+^ neutrophil masks using the MeasureObjectIntensity module. Mean Nox2 fluorescence intensity per tdTomato^+^ neutrophil was calculated for each image and averaged per animal.

To analyse percentage of tdTomato and Ly6G expression from lung sections, per-cell marker classification was performed using object-level intensity measurements in CellProfiler. For each DAPI-defined cell object, we measured mean tdTomato and Ly6G fluorescence intensities, applied predefined positivity thresholds for each marker based on negative and single-stain controls, and assigned each cell to one of four classes: tdTomato^−^/Ly6G^−^, tdTomato^+^/Ly6G^−^, tdTomato^−^/Ly6G^+^ or tdTomato^+^/Ly6G^+^. Counts and percentages for each class were then calculated per animal.

### DHE staining

For in situ ROS detection, mouse lungs were collected 18 h post-infection in PBS at room temperature. Immediately, lungs were embedded in OCT medium (VWR), frozen on dry ice, and sectioned at 30 μm using a Leica cryostat. Sections were mounted on Superfrost Plus glass slides (Thermo Fisher) and incubated with 10 μM dihydroethidium (DHE, Invitrogen) for 30 min at 37 °C in the dark, then washed 3 times for 5 min each in PBS. Coverslips were added with ProLong Diamond containing DAPI (all from Invitrogen) and immediately imaged with identical settings on a Nikon A1R confocal microscope.

For assessment of in vivo superoxide production by flow cytometry, BAL was collected in ice-cold PBS supplemented with 10% FCS (Hyclone) and centrifuged at 300 × *g* for 6 min at 4 °C. Cell pellets were resuspended in PBS and 1 × 10^6^ cells were stained with 100 μM DHE for 45 min at 37 °C in the dark. Cells were washed twice with FACS buffer, fixed in fixation buffer (Biolegend) for 20 min at 4 °C, and resuspended in FACS buffer for analysis on a Fortessa cell analyser (BD Biosciences).

### Oxyburst assay

For assessment of ex vivo ROS production, 1 × 10^6^ isolated human neutrophils were plated in 96-well plates and left to settle for 1 h. Cells were incubated with 10 µM OxyBURST Green H2DCFDA (Invitrogen) in Krebs-Ringer PBS (KRP buffer), which contains phosphate-buffered saline pH 7.4 with 1 mM Ca^2+^, 1.5 mM Mg^2+^ and 5.5 mM glucose. Samples were stimulated and acquired at 0, 10 and 30 min on a Fortessa cell analyser (BD Biosciences).

### Protein oxidation

Protein oxidation was measured by flow cytometry using the FlowCellect oxidative stress kit (Merck), following manufacturer’s instructions. This assay is based on the detection of protein carbonyl groups, a hallmark of oxidative protein damage, using 2,4-dinitrophenylhydrazine (DNPH) derivatisation. Briefly, carbonyl groups on oxidised proteins were derivatised to 2,4-dinitrophenylhydrazone (DNP) adducts by treatment with DNPH. These DNP-modified proteins were then detected using a fluorochrome-conjugated anti-DNP antibody. Samples were analysed by flow cytometry, and mean fluorescence intensity was used as a readout of protein carbonylation.

### Luminol assay

For plate-based ex vivo ROS detection, 1 × 10^6^ isolated human neutrophils were plated in white Nunclon 96-well plates and left to settle for 1 h at 37 °C. Total ROS production was measured by adding 100 µM cell-permeable luminol; extracellular ROS were assessed by adding 100 µM cell-impermeable isoluminol, both in the presence of 1.2 U/ml horseradish peroxidase (all chemicals Sigma-Aldrich). Chemiluminescence was measured immediately after stimulation every 30 s over 2 h on a Spectramax plate reader (Molecular Devices).

### Quantitative real-time RT-PCR

Total RNA from mouse lungs stabilised in RNAlater solution (Invitrogen) as well as mouse and human neutrophils was isolated using TriReagent-Chloroform extraction followed by isopropanol precipitation (all chemicals from Sigma-Aldrich). For analysis of interferon mRNA levels, samples were additionally purified using the RNeasy kit (Qiagen). Subsequently, 1 µg RNA were reverse transcribed to generate cDNA with the Transcriptor First strand cDNA synthesis kit (Roche) using anchored-oligo(dT)_18_ primer. Gene expression was measured in triplicates using TaqMan Universal PCR Master Mix with gene-specific primers and probes on a QuantStudio 7 Real-Time PCR machine (all from Applied Biosystems). The cycling-threshold (CT) for each gene was measured and normalised to that of the housekeeping gene (HPRT1 or B2M). The relative gene expression was calculated by the change-in-cycling-threshold (ΔΔCT) method.

### Subcellular fractionation and p65 binding assay

5 × 10^6^ neutrophils were seeded in 10 cm cell culture dishes. Forty-five minutes post-stimulation, cells were lysed using the Nuclear Extraction Kit (Cambridge Bioscience) according to the manufacturer’s instructions. Nuclear lysates containing 10 µg protein were used in a TransAM NFκB p65 transcription factor assay (Active Motif).

### Enzyme-linked immunosorbent assay

4 × 10^5^ neutrophils human or mouse neutrophils were seeded in a 96-well U-bottom plate. Cell culture supernatant was collected 5 h post-stimulation. In co-culture experiments, murine γδ T cells and neutrophils were combined in a 4:1 ratio. For cell interaction studies, γδ T cells were seeded in the bottom compartment of a Transwell plate. To block IL-1β signalling, 5 µg/ml anti-IL-1β antibody (BioXcell) was added. Supernatants were analysed using human IL-1β, mouse IL-1β and mouse IL-17A ELISA kits (all from Invitrogen) according to the manufacturer’s instructions.

### Statistics and reproducibility

Experiments were designed to use the minimum number of animals and human participants required to detect the hypothesised effects, in accordance with ethical principles and sample sizes were informed by power calculations. Animals and human samples were randomly allocated to experimental groups. Littermate controls were used for animal experiments wherever applicable to minimise genetic variability. For in vivo infection experiments, successful infection was confirmed by detecting influenza A virus M1 transcripts by quantitative PCR, and animals in which no viral burden was detected were excluded from the analysis. Investigators were not blinded to group allocation during experiments, except for histopathological assessment, which was performed blinded. All experiments were reproduced in at least two independent experiments, with the numbers of biological replicates and independent experiments specified in the corresponding figure legends. Statistical analyses and graph preparation were performed using GraphPad Prism version 10. The statistical tests applied to individual datasets, the definition of *n*, and the presentation of summary data are specified in the figure legends. All statistical tests were two-sided, and adjustments for multiple comparisons were applied where appropriate. A *P* value of less than 0.05 was considered statistically significant.

### Reporting summary

Further information on research design is available in the Nature Portfolio Reporting Summary linked to this article.

## Supplementary information

## Source data

## Data Availability

Source data are provided with this paper. The image data generated in this study have been deposited in the figshare database https://figshare.com/s/5b48e3e40145ff38822c. Further information and requests for resources and reagents should be directed to the corresponding author. Source data are provided with this paper.

## Code availability

Scripts and Cellprofiler pipelines used for Image analysis can be accessed on the GitHub Repository github.com/AnniWarn/Nox2^67^.
