# Supplementary material for: Neutrophil NADPH oxidase breaks the inflammatory IL-1β/IL-17A circuit to enhance pathogen clearance during respiratory virus infections
Source: Nat Commun. 2026 Aug 5;17:9425. doi: 10.1038/s41467-026-76327-4 (PMC13538623; doi:10.1038/s41467-026-76327-4)
Supplement: Supplementary file 1 [file 41467_2026_76327_MOESM1_ESM.pdf]

Supplementary information

for

Neutrophil NADPH oxidase breaks the inflammatory IL-1β/IL-17A circuit to enhance pathogen clearance during respiratory virus infections

Aderonke Sofoluwe[+], Angelos Petropoulos[+], Zehra F. Ali-Khan, Abhilesh Salil Goomanee, Simon J. Cleary and Annika Warnatsch[*]

[+] equally contributing first authors
[*] corresponding author

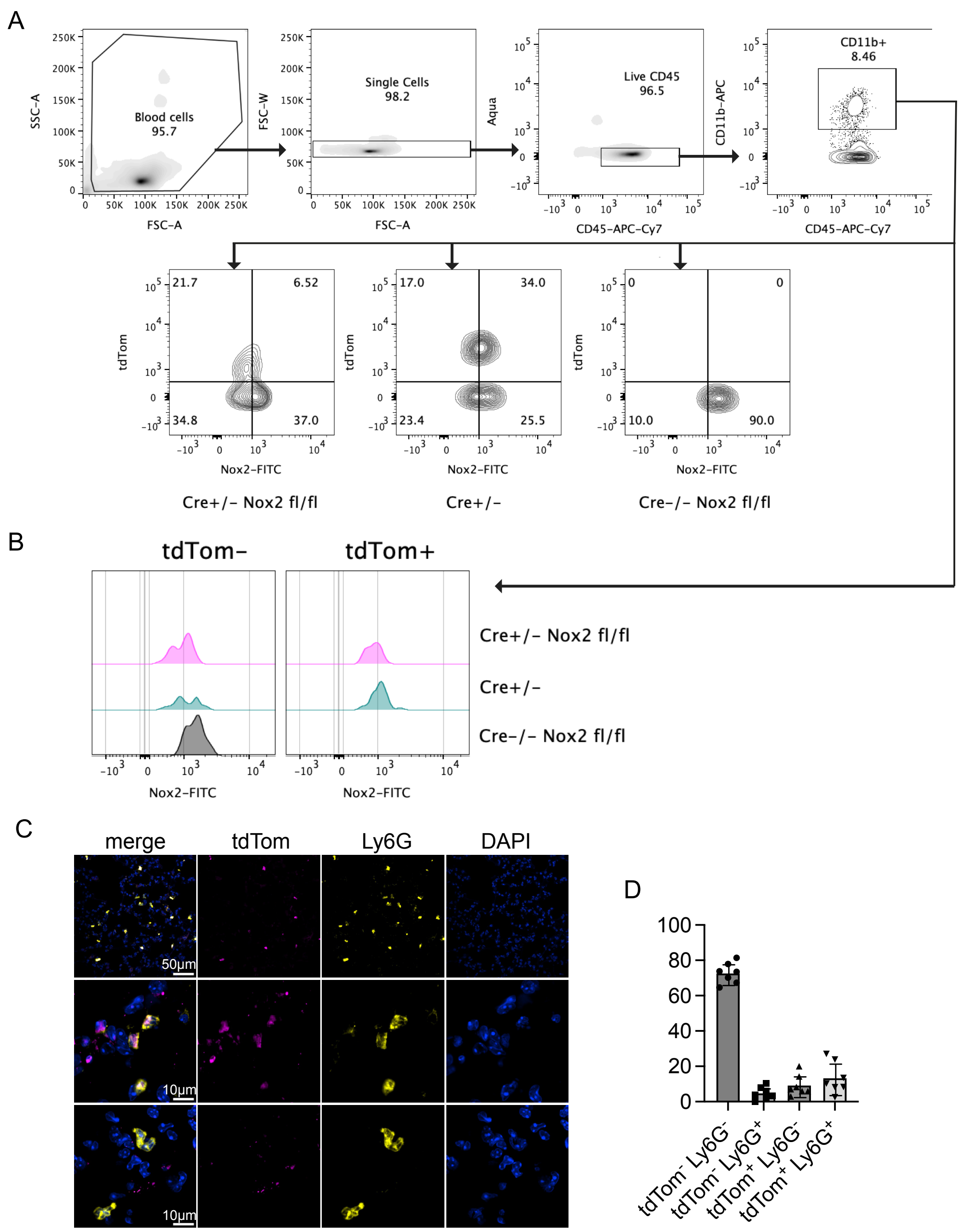

Supplementary Figure 1. Assessment of Ly6G-Cre driven tdTomato and Nox2 expression.

A) Gating strategy for naïve blood neutrophils gated as Live CD45$^+$, CD11b$^+$ and representative flow cytometry plots showing tdTomato versus Nox2 expression in male and female *Ly6G-Cre*$^{+/-}$ *Nox2*$^{fl/fl}$, *Ly6G-Cre*$^{+/-}$ and *Ly6G-Cre*$^{-/-}$ *Nox2*$^{fl/fl}$ littermates.

B) Representative histogram plots of Nox2 expression in tdTomato$^-$ (left panel) and tdTomato$^+$ (right panel) Live CD45$^+$, CD11b$^+$ blood cells from male and female *Ly6G-Cre*$^{+/-}$ *Nox2*$^{fl/fl}$, *Ly6G-Cre*$^{+/-}$ and *Ly6G-Cre*$^{-/-}$ *Nox2*$^{fl/fl}$ littermates.

D) Representative immunofluorescence micrographs of lung sections from *Ly6G-Cre*$^{+/-}$ *Nox2*$^{fl/fl}$ mice showing tdTomato expression (magenta), anti-Ly6G-Alexa Fluor647 staining (yellow) and nuclei (DAPI, blue). Scale bars: 50 µm (upper panels) and 10 µm (lower panels).

E) Percentage of tdTomato$^-$ Ly6G$^-$, tdTomato$^-$ Ly6G$^+$, tdTomato$^+$ Ly6G$^-$ and tdTomato$^+$ Ly6G$^+$ cells identified by DAPI staining in lung sections from male and female *Ly6G-Cre*$^{+/-}$ and *Ly6G-Cre*$^{+/-}$ *Nox2*$^{fl/fl}$ mice. Each data point represents one field of view from an independent animal (n = 7). Source data are provided as a Source Data file.

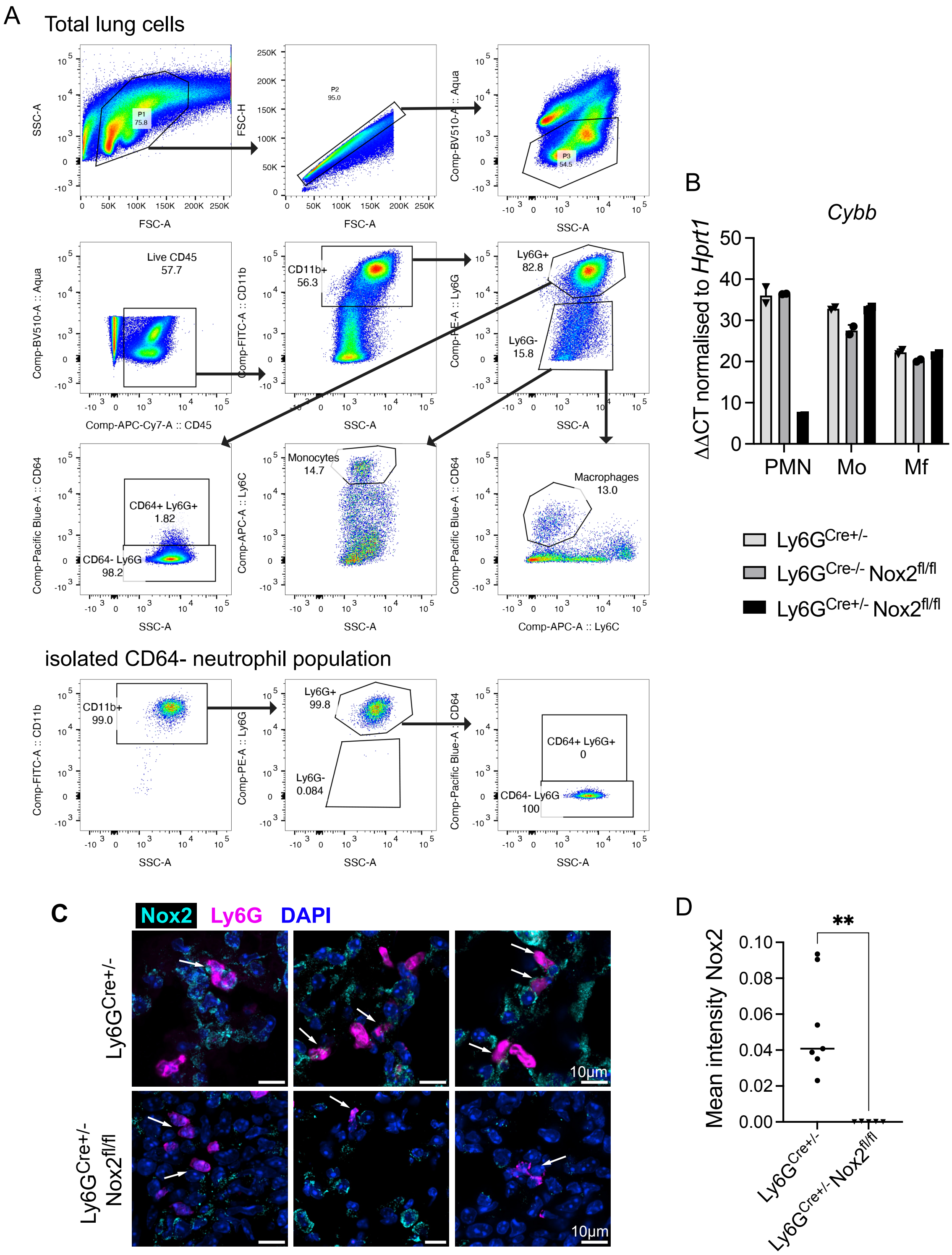

Supplementary Figure 2. Verification of neutrophil-specific Nox2 deletion by flow cytometric sorting and RT qPCR.

A) Gating strategy for flow-sorting of lung myeloid subsets, neutrophils (PMN, gated as Live $CD45^{+}$, $CD11b^{+}$, $Ly6G^{+}$, $CD64^{-}$), macrophages (Mf, gated as Live $CD45^{+}$, $CD11b^{+}$, $CD64^{+}$) and monocytes (Mo, gated as Live $CD45^{+}$, $CD11b^{+}$, $Ly6G^{-}$, $Ly6C^{hi}$). Lower panels show purity of isolated of PMN population.

B) Quantitative PCR analysis of *Nox2* (*Cybb*) expression in lung myeloid subsets sorted in (A), from *Ly6G-Cre*$^{+/-}$, *Ly6G-Cre*$^{-/-}$ *Nox2*$^{fl/fl}$ and *Ly6G-Cre*$^{+/-}$ *Nox2*$^{fl/fl}$ mice. DDCT values are normalised

to *Hprt*1. Bars represent mean ± SD of 2 technical replicates and are representative of 2 independent experiments. Source data are provided as a Source Data file.

C) Representative immunofluorescence micrographs of lung sections from *Ly6G-Cre*$^{+/-}$and *Ly6G-Cre*$^{+/-}$ *Nox2*$^{fl/fl}$ mice showing neutrophils (Ly6G^{tdTom}, magenta), Nox2 (gp19^{phox}, cyan) and nuclei (DAPI, blue). Scale bars 10 µm. White arrows point to neutrophils.

D) Mean Nox2 fluorescence intensity per tdTomato^{+} neutrophil measured in (C) and averaged per animal. Each data point represents one animal (*Ly6G-Cre*$^{+/-}$: n = 7, *Ly6G-Cre*$^{+/-}$ *Nox2*$^{fl/fl}$: n = 5). p value as indicated (**<0.01), determined using two-tailed Mann–Whitney test. Source data are provided as a Source Data file.

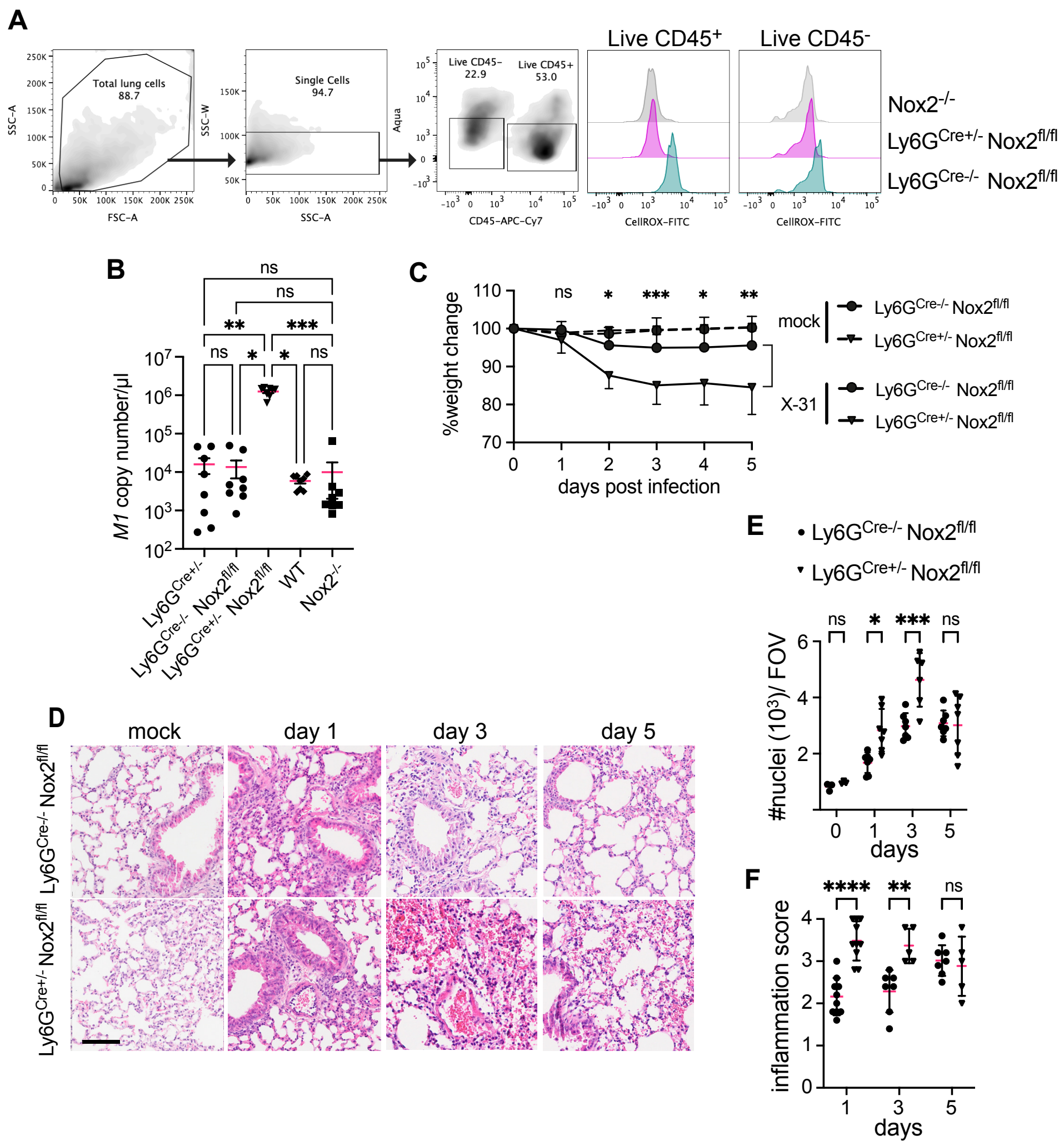

Supplementary Figure 3. Virus burden, weight loss and histopathology.

A) Gating strategy for Live CD45$^+$ and CD45$^-$ lung cells (left) and representative histogram plots of CellROX$^+$ CD45$^+$ and CD45$^-$ lung cells 3 days post-infection with IAV strain X-31 at 3x10^4 TCID$_{50}$ in *Nox2$^{-/-}$*, *Ly6G-Cre$^{+/-}$ Nox2$^{fl/fl}$ and Ly6G-Cre$^{-/-}$ Nox2$^{fl/fl}$* mice. Related to Figures 1C and D.

B) RT-qPCR of influenza matrix *M1* RNA in whole lungs 3 days post-infection with IAV strain X-31 at 3x10^4 TCID$_{50}$ in *Ly6G-Cre$^{+/-}$*, *Ly6G-Cre$^{-/-}$ Nox2$^{fl/fl}$*, *Ly6G-Cre$^{+/-}$ Nox2$^{fl/fl}$*, wild-type and *Nox2$^{-/-}$* mice (n = 8). Related to Figure 1B.

C) Weight loss monitored over 5 days post-infection with IAV strain X-31 at 3x10^4 TCID$_{50}$ in *Ly6G-Cre$^{-/-}$ Nox2$^{fl/fl}$* and *Ly6G-Cre$^{+/-}$ Nox2$^{fl/fl}$* mice (mock: n = 2, X-31: n = 8).

D) Representative micrographs of H&E-stained lung sections at indicated time points after infection with 3x10^4 TCID$_{50}$ X-31 in *Ly6G-Cre$^{+/-}$* and *Ly6G-Cre$^{+/-}$ Nox2$^{fl/fl}$* mice. Scale bar 100 µm.

E) Number of nuclei per field of view per animal at indicated time points after infection with 3x10^4 TCID$_{50}$ X-31 (day 0: n = 3, day 1 - 5: n =7).

F) Scoring of inflammation at indicated time points after infection with 3x10^4 TCID$_{50}$ X-31. The degree of inflammation was graded as follows: 0, normal; 1, mild; 2, moderate; 3, severe; and 4, very severe inflammation (n = 7).

Bars represent mean ± SEM (B) and mean ± SD (C, E, F) of 2 – 3 independent experiments (B - F). Each data point represents one animal (B, E, F). p values are indicated, *<0.05, **<0.01, ***<0.001, ****<0.0001, ns not significant. p values were determined using Kruskal-Wallis test and Dunn's multiple comparisons post-test (B), or two-way ANOVA and Sidak's post-test (C, E, F). Source data are provided as a Source Data file.

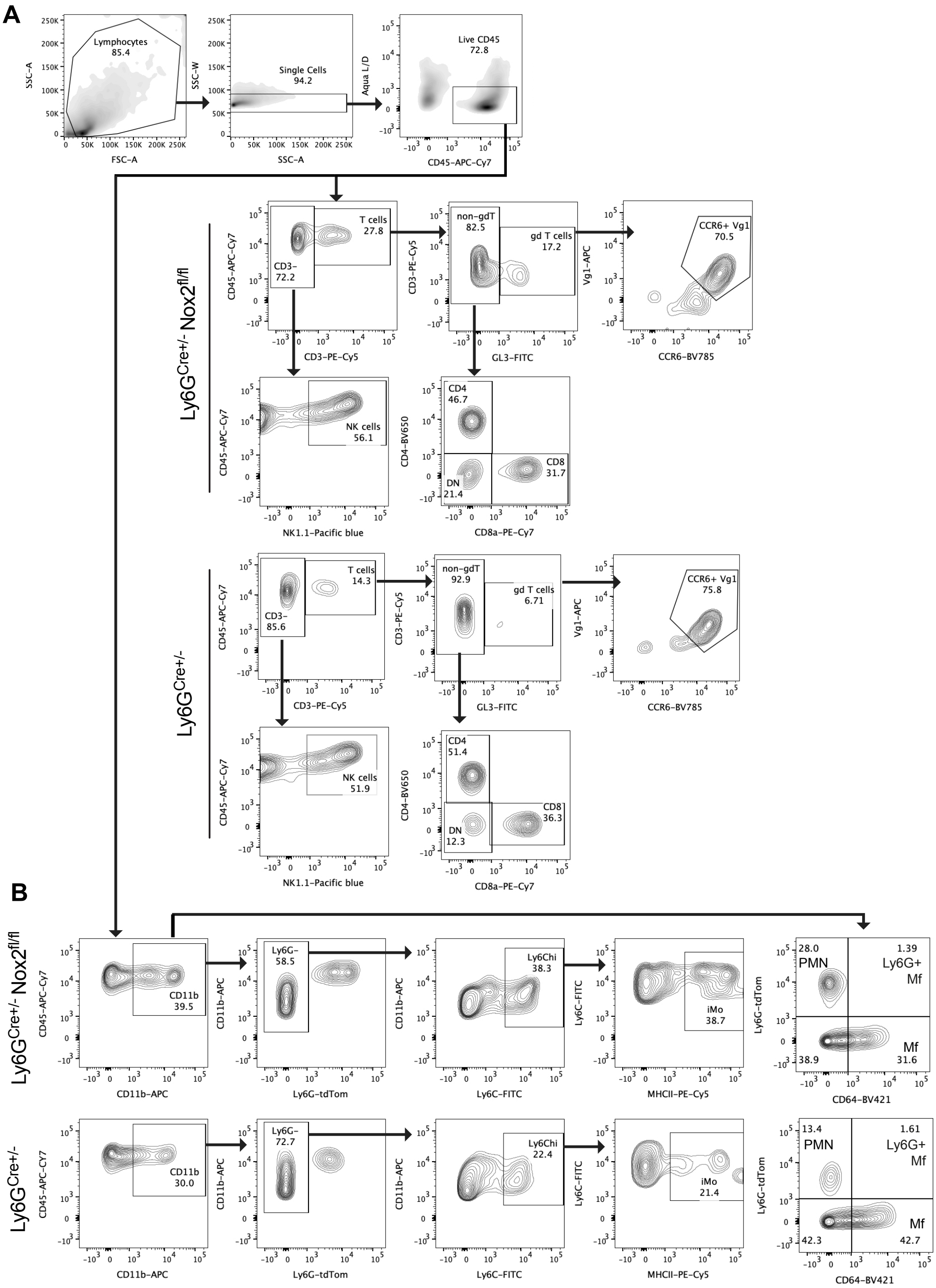

Supplementary Figure 4. Gating strategies.

Male and female *Ly6G-Cre*$^{+/-}$ and *Ly6G-Cre*$^{+/-}$ *Nox2*$^{fl/fl}$ mice were infected with $3x10^4$ $TCID_{50}$ IAV strain X-31 or PBS (mock) in 30 µl intranasally.

A) Gating strategy for NK (Live $CD45^+$, $CD3e^-$, NK1.1$^+$), γδ T (Live $CD45^+$, $CD3e^+$, γδ TCR^+, $V\gamma1^+$, $CCR6^+$), CD4 (Live $CD45^+$, $CD3e^+$, γδ TCR^-,$CD4^+$), CD8 (Live $CD45^+$, $CD3e^+$, γδ TCR^-, $CD8a^+$) and double negative (DN; Live $CD45^+$, $CD3e^+$, γδ TCR^-,$CD4^-$, $CD8a^-$) αβ T cells shown in *Ly6G-Cre*$^{+/-}$ and *Ly6G-Cre*$^{+/-}$ *Nox2*$^{fl/fl}$ mice. Related to Figures 2F – K, 3E – J, Supplementary Figures 5E – F, 7B, 9C – L, 10, 11.

B) Gating strategy for neutrophils (PMN; Live $CD45^+$, $CD11b^{hi}$, $Ly6G^+$, $CD64^-$), macrophages (Mf; Live $CD45^+$, $CD11b^{hi}$, $Ly6G^-$, $CD64^+$) and $Ly6C^{hi}$ monocytes (iMo; Live $CD45^+$, $CD11b^{hi}$, $Ly6G^-$, $Ly6C^{hi}$, $MHCII^+$) shown in *Ly6G-Cre*$^{+/-}$ and *Ly6G-Cre*$^{+/-}$ *Nox2*$^{fl/fl}$ mice. Related to Figures 2A – E, 3A - D, Supplementary Figures 5G – J, 7A, D, K, 8A - G.

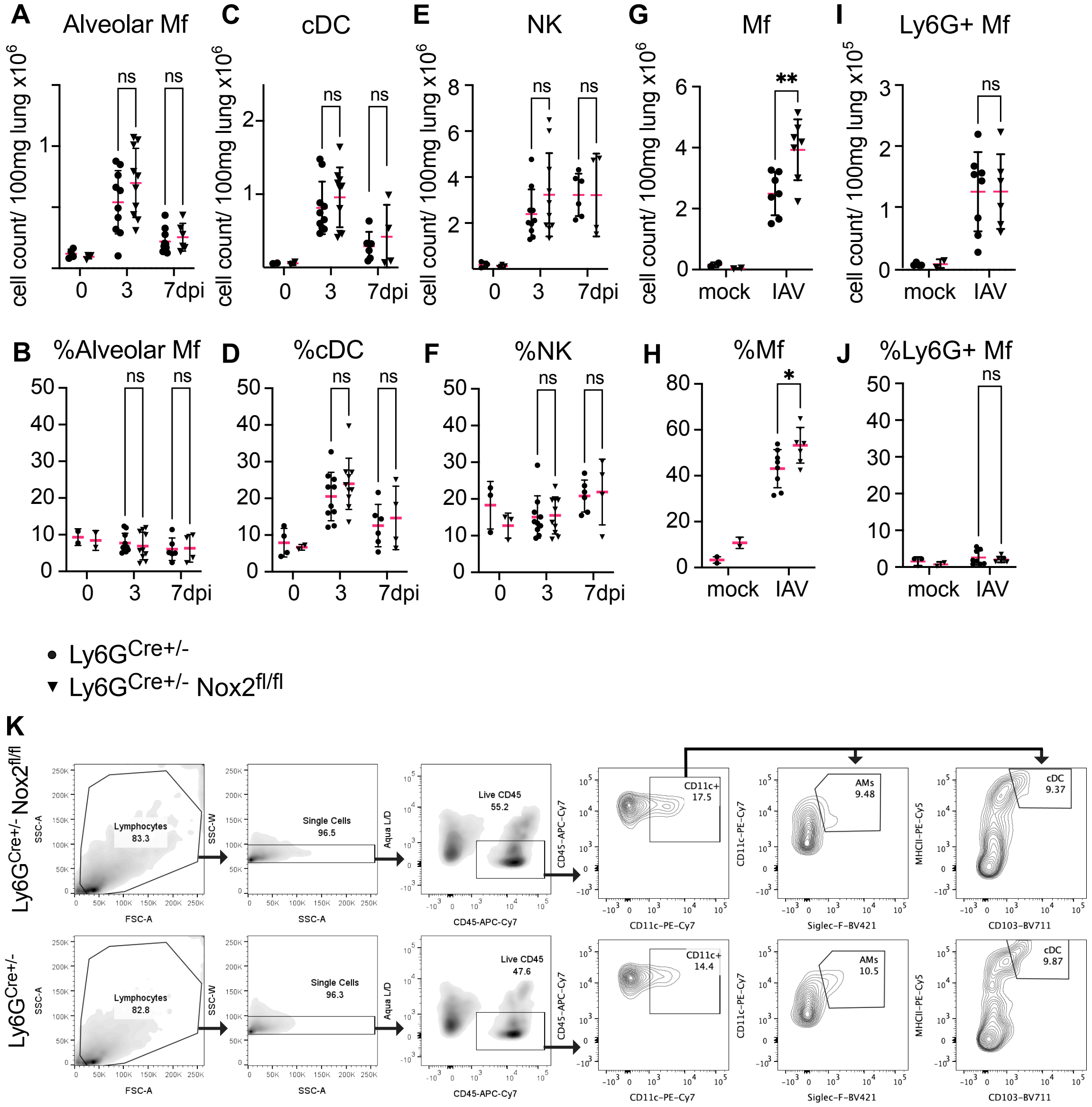

Supplementary Figure 5. Myeloid cell infiltration.

Male and female *Ly6G-Cre*$^{+/-}$ and *Ly6G-Cre*$^{+/-}$ *Nox2*$^{fl/fl}$ mice were infected with $3x10^4$ $TCID_{50}$ IAV strain X-31 or PBS (mock) in 30 µl intranasally.

A – J) Absolute counts per 100 mg lung tissue and frequency of alveolar macrophages gated as Live $CD45^+$, $CD11c^+$, Siglec-F^+ (A, B), conventional dendritic cells gated as Live $CD45^+$, $CD11c^+$, $CD103^+$, $MHCII^+$ (C, D), NK cells gated as Live $CD45^+$, $CD3e^-$ and $NK1.1^+$ (E, F) at 3 and 7 days post-infection, and mature macrophages gated as Live $CD45^+$, $CD11b^+$, $CD64^+$ (G, H) and $Ly6G^+$, $CD64^+$ macrophages (I, J) at 3 and 7 days post-infection (day 0: n = 2, day 3: n = 10, day 7: n = 6 *Ly6G-Cre*$^{+/-}$ and 4 *Ly6G-Cre*$^{+/-}$ *Nox2*$^{fl/fl}$).

K) Gating strategy for alveolar macrophages (AM; Live $CD45^+$, $CD11c^+$, Siglec-F^+) and conventional dendritic cells (cDC; Live $CD45^+$, $CD11c^+$, $CD103^+$, $MHCII^+$) shown in *Ly6G-Cre*$^{+/-}$ and *Ly6G-Cre*$^{+/-}$ *Nox2*$^{fl/fl}$ mice. Related to Supplementary Figures 5A – D, 7E – H.

Bars represent mean ± SD of 4 independent experiments (A - J). Each data point represents one animal. p values are indicated, *<0.05, **<0.01, ns not significant. p values were determined using Two-way ANOVA and Sidak's post-test. Source data are provided as a Source Data file.

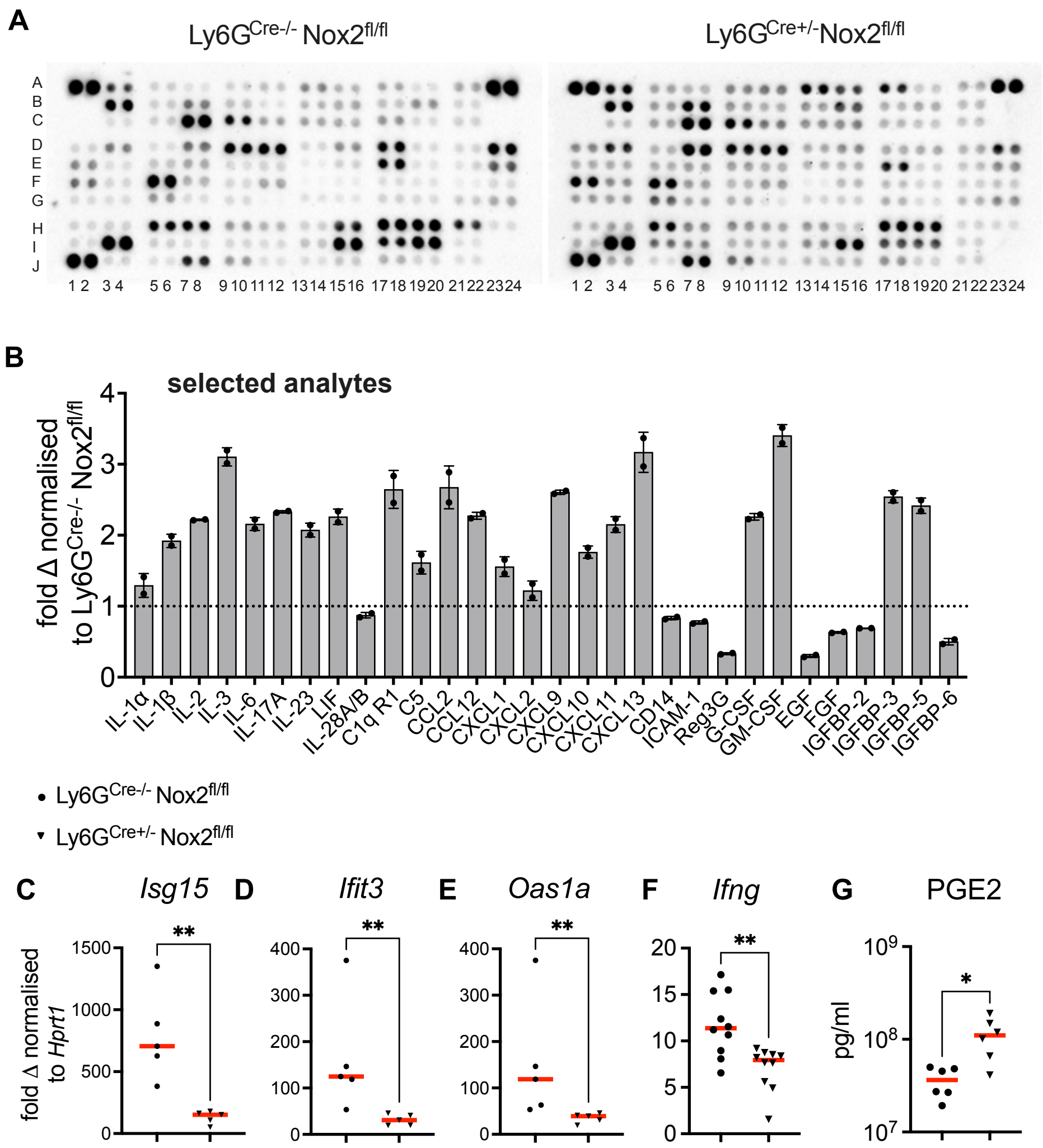

Supplementary Figure 6. Cytokine array.

Male and female *Ly6G-Cre*$^{-/-}$ *Nox2*$^{fl/fl}$ and *Ly6G-Cre*$^{+/-}$ *Nox2*$^{fl/fl}$ mice were infected with $3x10^4$ $TCID_{50}$ IAV strain X-31 or PBS (mock) in 30 µl intranasally.

A) Representative nitrocellulose membranes of cytokine/chemokine arrays (proteome profiler by R&D) incubated with bronchoalveolar lavage (BAL) from *Ly6G-Cre*$^{-/-}$ *Nox2*$^{fl/fl}$ and *Ly6G-Cre*$^{+/-}$ *Nox2*$^{fl/fl}$ mice infected with IAV strain X-31 at $3x10^4$ $TCID_{50}$ for 3 days. BAL samples were pooled from 5 mice per experimental group across 4 independent experiments. Coordinates of 111 analytes are shown. Each cytokine/ chemokine is spotted in duplicate.

B) Mean pixel density of selected interleukines and chemokines of *Ly6G-Cre*$^{+/-}$ *Nox2*$^{fl/fl}$ mice identified in the array shown in (A), calculated as fold change relative to infected *Ly6G-Cre*$^{-/-}$ *Nox2*$^{fl/fl}$ animals.

C – F) *Isg15, Ifit3, Oas1a* (n = 5) and *Ifng* (n = 10) mRNA expression as fold change relative to mock-infected normalised to *Hprt1* in lung tissue of *Ly6G-Cre*$^{-/-}$ *Nox2*$^{fl/fl}$ and *Ly6G-Cre*$^{+/-}$ *Nox2*$^{fl/fl}$ animals 2 days post-infection was quantified by RT-qPCR.

G) Prostaglandin E2 (PGE2) protein in bronchoalveolar lavage of *Ly6G-Cre*$^{-/-}$ *Nox2*$^{fl/fl}$ and *Ly6G-Cre*$^{+/-}$ *Nox2*$^{fl/fl}$ animals 3 days post-infection was quantified by ELISA (n = 6).

Bars represent mean ± SD of 4 (B) and mean of 2 - 3 independent experiments (C - G). Each data point represents one animal (C - G). p values are indicated, *<0.05, **<0.01. p values were determined using two-tailed Mann-Whitney test (C - G). Source data are provided as a Source Data file.

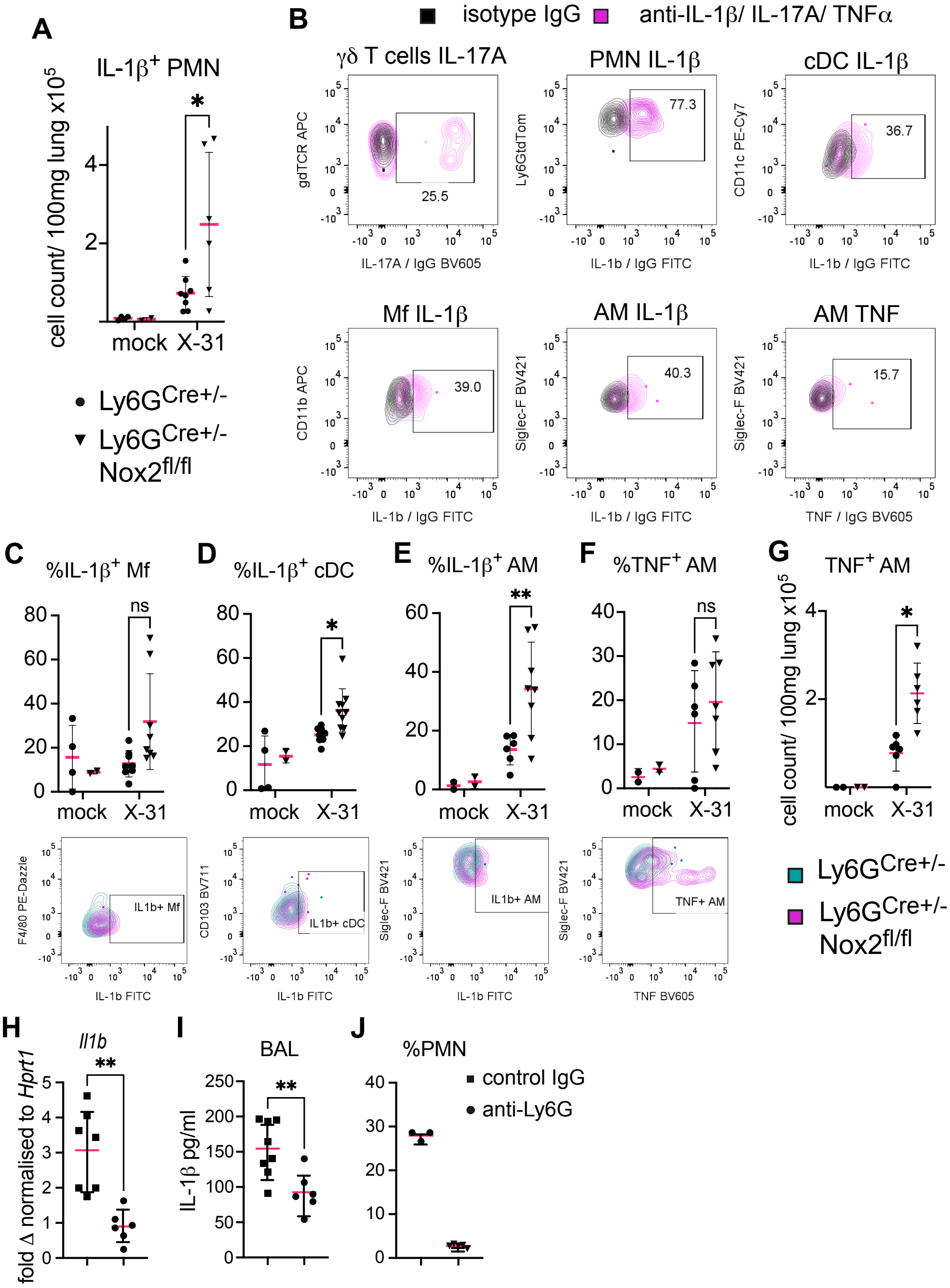

Supplementary Figure 7. Cellular cytokine production.

Male and female *Ly6G-Cre*$^{+/-}$ and *Ly6G-Cre*$^{+/-}$ *Nox2*$^{fl/fl}$ mice were infected with $3x10^4$ $TCID_{50}$ IAV strain X-31 or PBS (mock) in 30 µl intranasally.

A) Absolute cell counts per 100mg lung tissue of IL-1β$^+$ neutrophils gated as Live CD45$^+$, CD11b$^+$ an Ly6G^{tdTom} (*Ly6G-Cre*$^{+/-}$: n = 4 mock and n = 6 X-31, *Ly6G-Cre*$^{+/-}$ *Nox2*$^{fl/fl}$: n = 2 mock, n = 8 X-31). Related to Figure 3B.

B) Representative flow cytometry plots of isotype staining (black) and cytokine staining (magenta) of IL-17A$^+$ γδ T cells gated as Live CD45$^+$, CD3e$^+$ and γδTCR$^+$, IL-1β$^+$ neutrophils (PMN) gated as Live CD45$^+$, CD11b$^+$ an Ly6G^{tdTom}, IL-1β$^+$ conventional dendritic cells (cDC) gated as CD11c$^+$, CD103$^+$ and MHCII$^+$, IL-1β$^+$ macrophages (Mf) gated as CD11b$^+$, F4/80$^+$ and MHCII$^+$, IL-

$1\beta^+$ and $TNF\alpha^+$ alveolar macrophages (AM) gated as Live $CD45^+$, $CD11c^+$ and Siglec-F^+ at 3 days post-infection in lungs of *Ly6G-Cre*$^{+/-}$ *Nox2*$^{fl/fl}$ mice.

C - F) Frequency and representative flow cytometry plots of IL-$1\beta^+$ macrophages gated as $CD11b^+$, F4/80^+ and $MHCII^+$ (C), IL-$1\beta^+$ conventional dendritic cells gated as $CD11c^+$, $CD103^+$ and $MHCII^+$ (D), IL-$1\beta^+$ Alveolar macrophages (AM) gated as $CD11c^+$ and Siglec-F^+ (E) and $TNF\alpha^+$ AM (F) at 3 days post-infection (*Ly6G-Cre*$^{+/-}$: n = 4 mock and n = 6 X-31, *Ly6G-Cre*$^{+/-}$ *Nox2*$^{fl/fl}$: n = 2 mock, n = 8 X-31).

G) Absolute cell count per 100mg lung tissue of $TNF\alpha^+$ AM at 3 days post-infection (n = 2 mock, n = 6 X-31).

H - J) *Ly6G-Cre*$^{+/-}$ *Nox2*$^{fl/fl}$ mice were treated with 100 µg anti-Ly6G or control IgG antibody in 200 µl intraperitoneally at -1 day, -1 hour prior- and +1 day post-infection with IAV strain X-31 at $3x10^4$ $TCID_{50}$ or PBS (mock) in 30 µl intranasally. mRNA expression of *Il1b* as fold change from naïve normalised to *Hprt1* assessed by RTqPCR in lung tissue 2 days post-infection (I, n = 6). IL-1β protein in pg/ml bronchoalveolar lavage 2 days post-infection by ELISA (J, n = 8 control IgG and n = 6 anti-Ly6G). Frequency of neutrophils gated as Live $CD45^+$ $CD11b^+$ $Ly6G^+$ $CD64^-$ in blood 2 days post-infection (K, n = 3 control IgG and n = 5 anti-Ly6G).

Bars represent mean ± SD of 2 - 4 independent experiments (A - J). Each data point represents one animal. p values are indicated, $^*<0.05$, $^{**}<0.01$, ns not significant. p values were determined using Two-way ANOVA and Sidak's post-test (A – G) or two-tailed Mann-Whitney test (H - I). Source data are provided as a Source Data file.

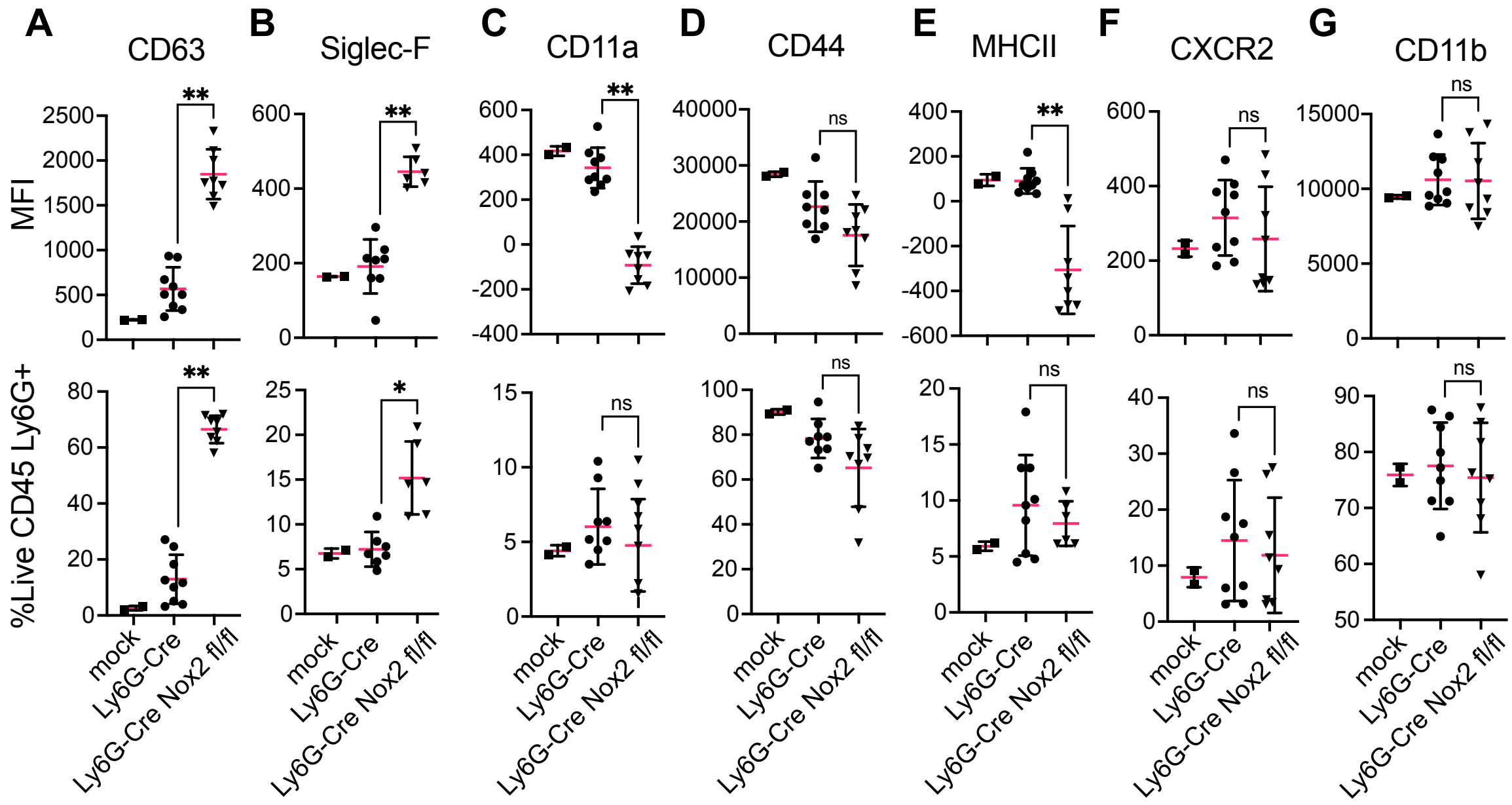

Supplementary Figure 8. Characterisation of neutrophils.

Male and female *Ly6G-Cre*$^{+/-}$ and *Ly6G-Cre*$^{+/-}$ *Nox2*$^{fl/fl}$ mice were infected with $3x10^4$ $TCID_{50}$ IAV strain X-31 or PBS (mock) in 30 µl intranasally.

A – G) Geometric mean fluorescence intensity (MFI) and frequency of CD63 (A), Siglec-F (B), CD11a (C), CD44 (D), MHC class 2 (E), CXCR2 (F) and CD11b (G) expression on lung neutrophils gated as Live $CD45^+$, $CD11b^+$, $Ly6G^+$ and $CD64^-$ in lung tissue at 3 days post-infection (n = 2 mock, n = 8 X-31). Bars represent mean ± SD of 4 independent experiments. Each data point represents one animal. p values are indicated, *<0.05, **<0.01, ***<0.001, ****<0.0001, ns not significant. p values were determined using Kruskal Wallis and Dunn's post-test. Source data are provided as a Source Data file.

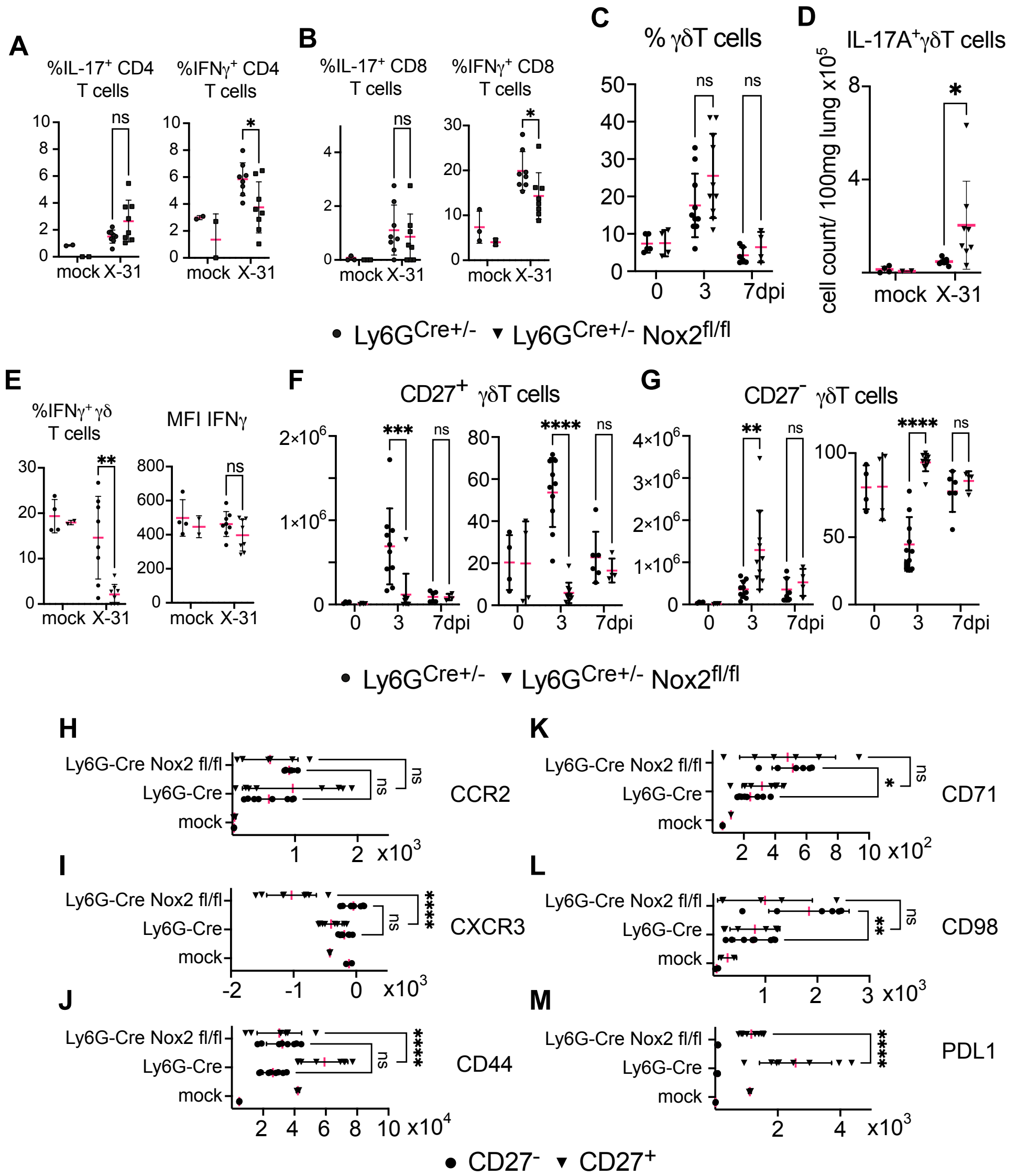

Supplementary Figure 9. Characterisation of αβ and γδ T cells.

Male and female *Ly6G-Cre*$^{+/-}$ and *Ly6G-Cre*$^{+/-}$ *Nox2*$^{fl/fl}$ mice were infected with $3x10^4$ $TCID_{50}$ IAV strain X-31 or PBS (mock) in 30 µl intranasally.

A - B) Frequency of IL-17$^+$ and IFNγ$^+$ CD4$^+$ (A) and CD8$^+$ (B) T cells gated as Live CD45$^+$, CD3e$^+$ and γδTCR$^-$ at 3 days post-infection (n = 2 mock, n = 8 X-31).

C) Frequency of γδ T cells gated as Live CD45$^+$, CD3e$^+$ and γδTCR$^+$ in lung 3- and 7-days post-infection (day 0: n = 4, day 3: n = 10 both genotypes, day 7: n = 6 *Ly6G-Cre*$^{+/-}$ and n= 4 *Ly6G-Cre*$^{+/-}$ *Nox2*$^{fl/fl}$).

D) Absolute cell counts per 100mg lung tissue of IL-17A$^+$ γδ T cells gated as Live CD45$^+$, CD3e$^+$ and γδTCR$^+$ (*Ly6G-Cre*$^{+/-}$: n = 4 mock and n = 6 X-31, *Ly6G-Cre*$^{+/-}$ *Nox2*$^{fl/fl}$: n = 2 mock, n = 8 X-31). Related to Figure 3F.

E) Frequency (left panel) and geometric mean fluorescence intensity (MFI, right panel) of IFNγ$^+$ γδ T cells at 3 days post-infection (n = 2 mock, n = 8 X-31).

F - G) Absolute cell count per 100 mg lung tissue (left) and frequency (right) of CD27$^+$ (F) and CD27$^-$ (G) γδ T cells 3- and 7-days post-infection (day 0: n = 4, day 3: n = 10 both genotypes, day 7: n = 6 *Ly6G-Cre*$^{+/-}$ and n= 4 *Ly6G-Cre*$^{+/-}$ *Nox2*$^{fl/fl}$).

H – M) Geometric mean fluorescence intensity (MFI) of CCR2 (H), CXCR3 (I), CD44 (J), CD71 (K), CD98 (L) and PDL1 (M) expression on $CD27^+$ and $CD27^-$ γδ T cells in lung tissue at 3 days post-infection (mock: n = 2, *Ly6G-Cre*$^{+/-}$ n = 8, *Ly6G-Cre*$^{+/-}$ *Nox2*$^{fl/fl}$ n = 6).

Bars represent mean ± SD of 4 independent experiments (A - M). Each data point represents one animal. p values are indicated, *<0.05, **<0.01, ***<0.001, ****<0.0001, ns not significant. p values were determined using Two-way ANOVA and Sidak's post-test (A - G), or Two-way ANOVA and Tukey's post-test (H - M). Source data are provided as a Source Data file.

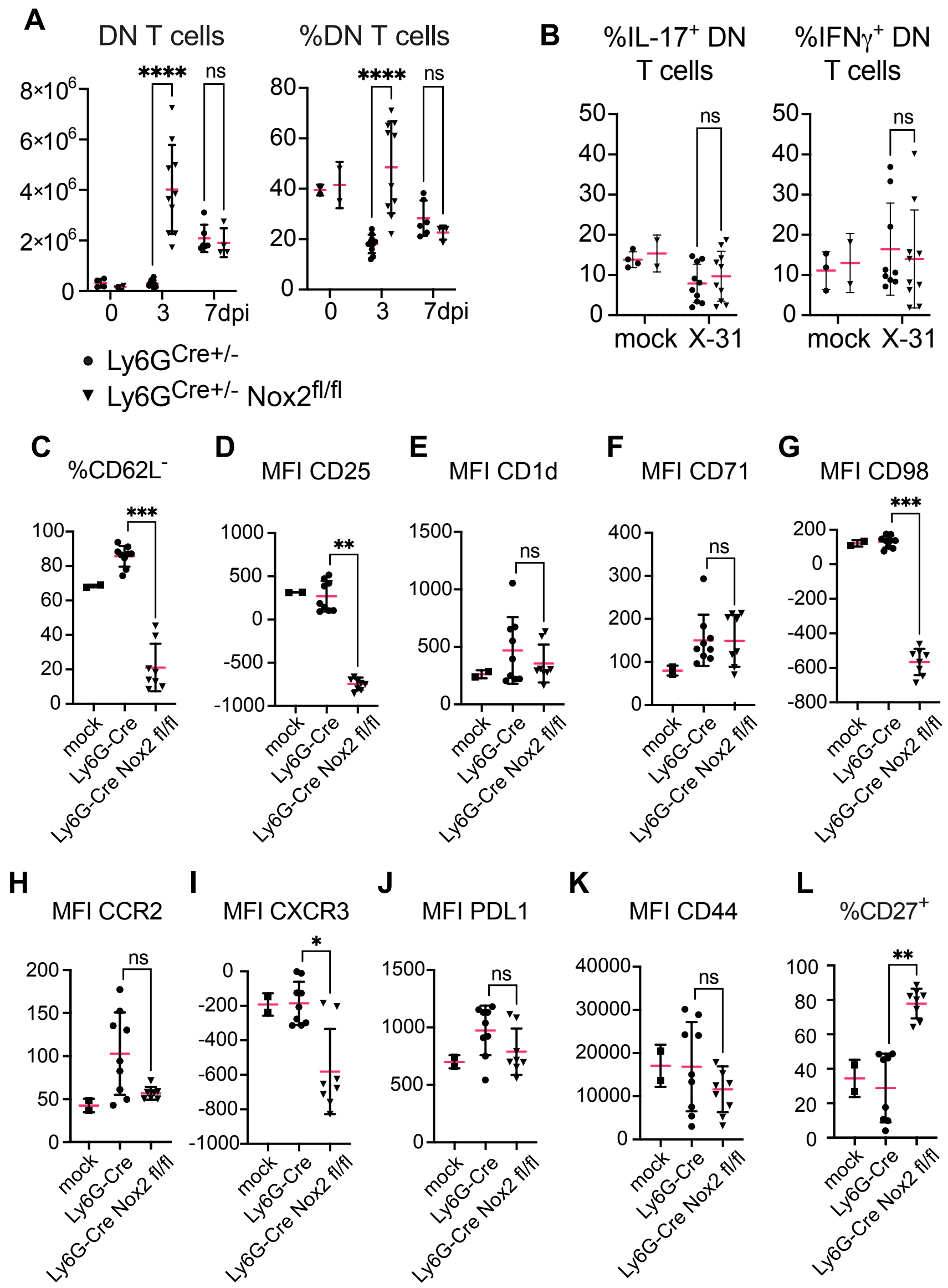

Supplementary Figure 10. Characterisation of DN T cells.

Male and female *Ly6G-Cre*$^{+/-}$ and *Ly6G-Cre*$^{+/-}$ *Nox2*$^{fl/fl}$ mice were infected with $3x10^4$ $TCID_{50}$ IAV strain X-31 or PBS (mock) in 30 µl intranasally.

A) Absolute cell count per 100 mg lung tissue (left) and frequency (right) of DN T cells gated as Live CD45$^+$, CD3e$^+$, γδTCR$^-$, CD4$^-$ and CD8$^-$ 3- and 7-days post-infection (day 0: n = 4, day 3 n = 10, day 7: n = 5).

B) Frequency of IL-17$^+$ and IFNγ$^+$ DN T cells gated as Live CD45$^+$, CD3e$^+$, γδTCR$^-$, CD4$^-$ and CD8$^-$ at 3 days post-infection (n = 2 mock, n = 10 X-31).

C) Frequency of CD62L$^-$ DN T cells (mock: n = 2, *Ly6G-Cre*$^{+/-}$ n = 9, *Ly6G-Cre*$^{+/-}$ *Nox2*$^{fl/fl}$ n = 8).

D - K) Geometric mean fluorescence intensity (MFI) of CD25 (D), CD1d (E), CD71 (F), CD98 (G), CCR2 (H), CXCR3 (I), PDL1 (J) and CD44 (K) DN T cells (mock: n = 2, *Ly6G-Cre*$^{+/-}$ n = 9, *Ly6G-Cre*$^{+/-}$ *Nox2*$^{fl/fl}$ n = 8).

L) Frequency of CD27$^+$ DN T cells (mock: n = 2, *Ly6G-Cre*$^{+/-}$ n = 9, *Ly6G-Cre*$^{+/-}$ *Nox2*$^{fl/fl}$ n = 8).

Bars represent mean ± SD of 4 independent experiments (A - L). Each data point represents one animal. p values are indicated, *<0.05, **<0.01, ***<0.001, ****<0.0001, ns not significant. p values were determined using Two-way ANOVA and Sidak's post-test (A - B) or Kruskal-Wallis and Dunn's post-test (C - L). Source data are provided as a Source Data file.

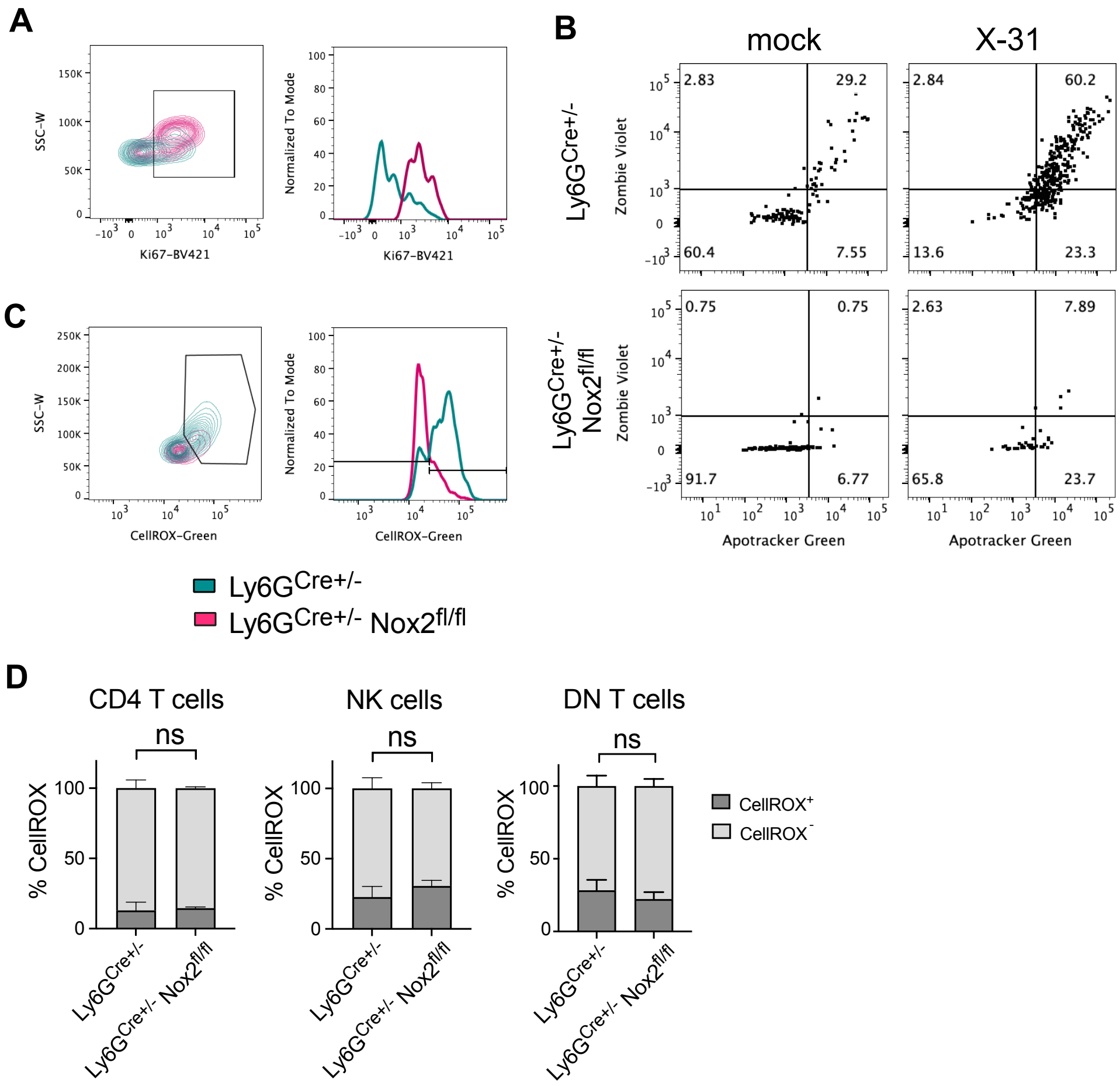

Supplementary Figure 11. Proliferation, apoptosis and oxidative stress.

Male and female *Ly6G-Cre*$^{+/-}$ and *Ly6G-Cre*$^{+/-}$ *Nox2*$^{fl/fl}$ mice were infected with $3x10^4$ $TCID_{50}$ IAV strain X-31 or PBS (mock) in 30 µl intranasally.

A) Representative flow cytometry (left panel) and histogram (right panel) plot of Ki67$^+$ γδ T cells gated as Live CD45$^+$, CD3e$^+$ and γδTCR$^+$ in lungs at 3 days post-infection. Related to Figure 3H.

B) Representative flow cytometry plots of ZombieViolet$^+$ and Apotracker$^+$ γδ T cells in lungs at 3 days post-infection. Related to Figure 3H.

C) Representative flow cytometry (left panel) and histogram (right panel) plot of CellROX$^+$ γδ T cells in lungs at 3 days post-infection. Related to Figure 3J.

D) Frequency of CellROX$^+$ CD4 T cells gated as Live CD45$^+$, CD3e$^+$, γδTCR$^-$, CD4$^+$ (left panel, n = 3), NK cells gated as Live CD45$^+$, CD3e$^-$ and NK1.1$^+$ (middle panel, n = 3) and DN T cells gated as Live CD45$^+$, CD3e$^+$, γδTCR$^-$, CD4$^-$ and CD8$^-$ (left panel, n = 3) in lungs at 3 days post-infection. Bars represent mean ± SD of 2 independent experiments. p values are indicated, ns not significant. p values were determined using two-tailed Mann Whitney test.

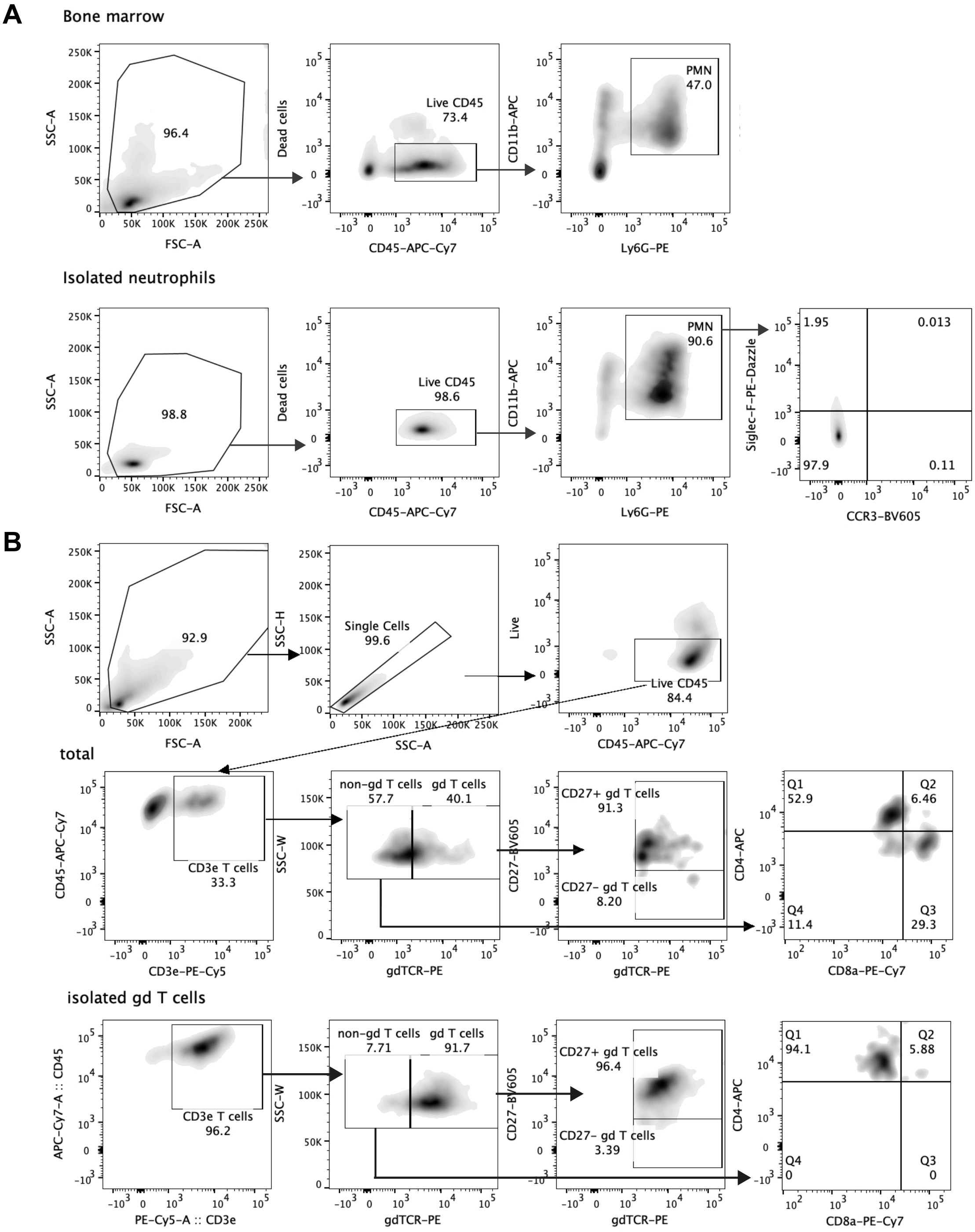

Supplementary Figure 12. Isolation of mouse neutrophils and γδ T cells from bone marrow.

Neutrophils were isolated from bone marrow and γδ T cells from spleen and lymph nodes of naïve C57Bl/6 and *Nox2*$^{-/-}$ mice.

(A) Gating strategy and representative flow cytometry plots of neutrophils before (upper panels) and after isolation (lower panels) gated as Live CD45^{+}, CD11b^{+}, Ly6G^{+}, CCR3^{-} and Siglec-F^{-}.

(B) Gating strategy and representative flow cytometry plots of γδ T cells before (upper panels) and after isolation (lower panels) gated as Live CD45^{+}, CD3e^{+}, γδTCR^{+}, CD4^{-} and CD8^{-}. Related to Figure 4.

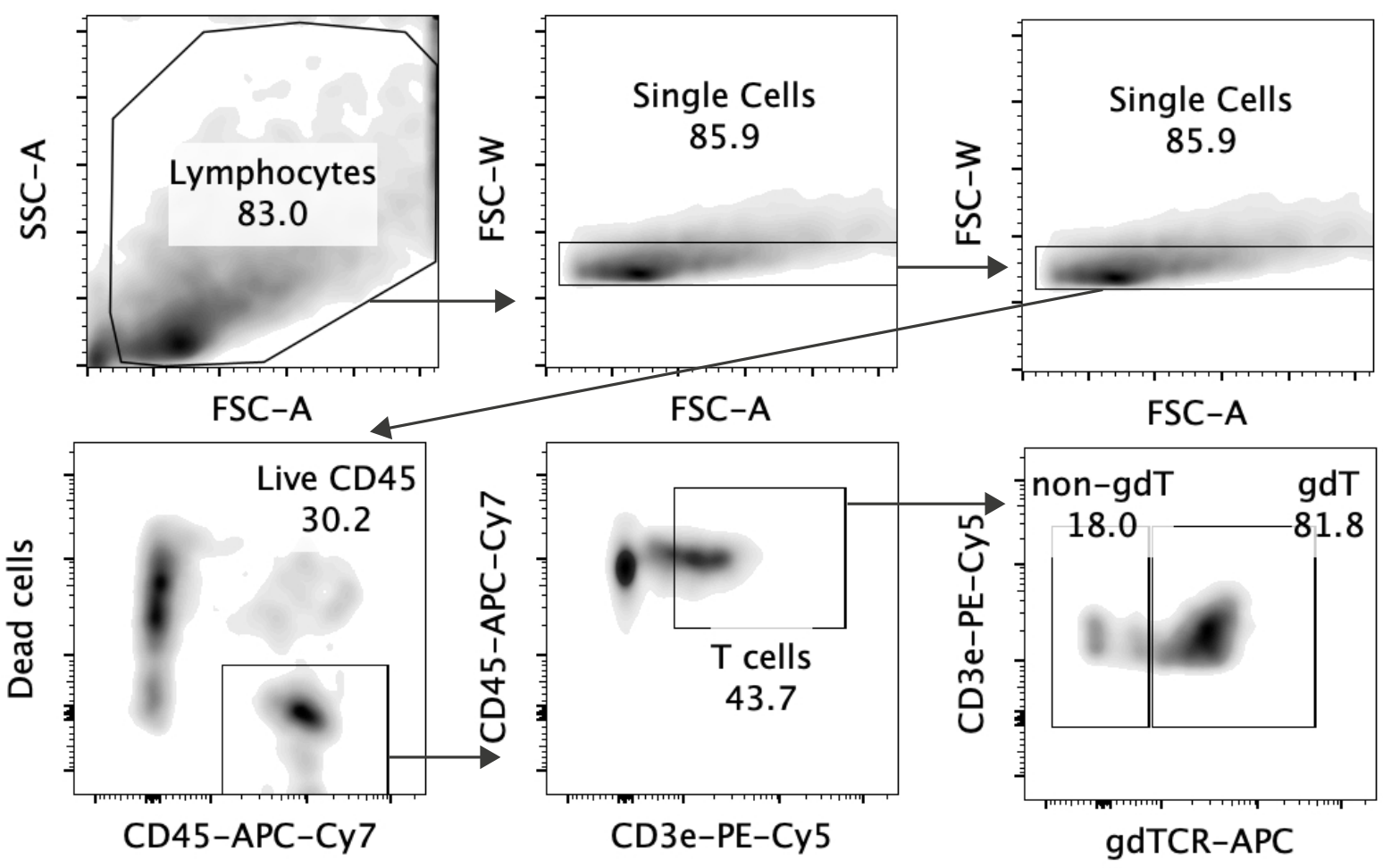

Supplementary Figure 13. Isolation of mouse γδ T cells from lung

Gating strategy and representative flow cytometry plots of γδ T cells after isolation from lungs of C57Bl/6J wild-type mice gated as Live CD45^{+}, CD3e^{+}, γδTCR^{+}.

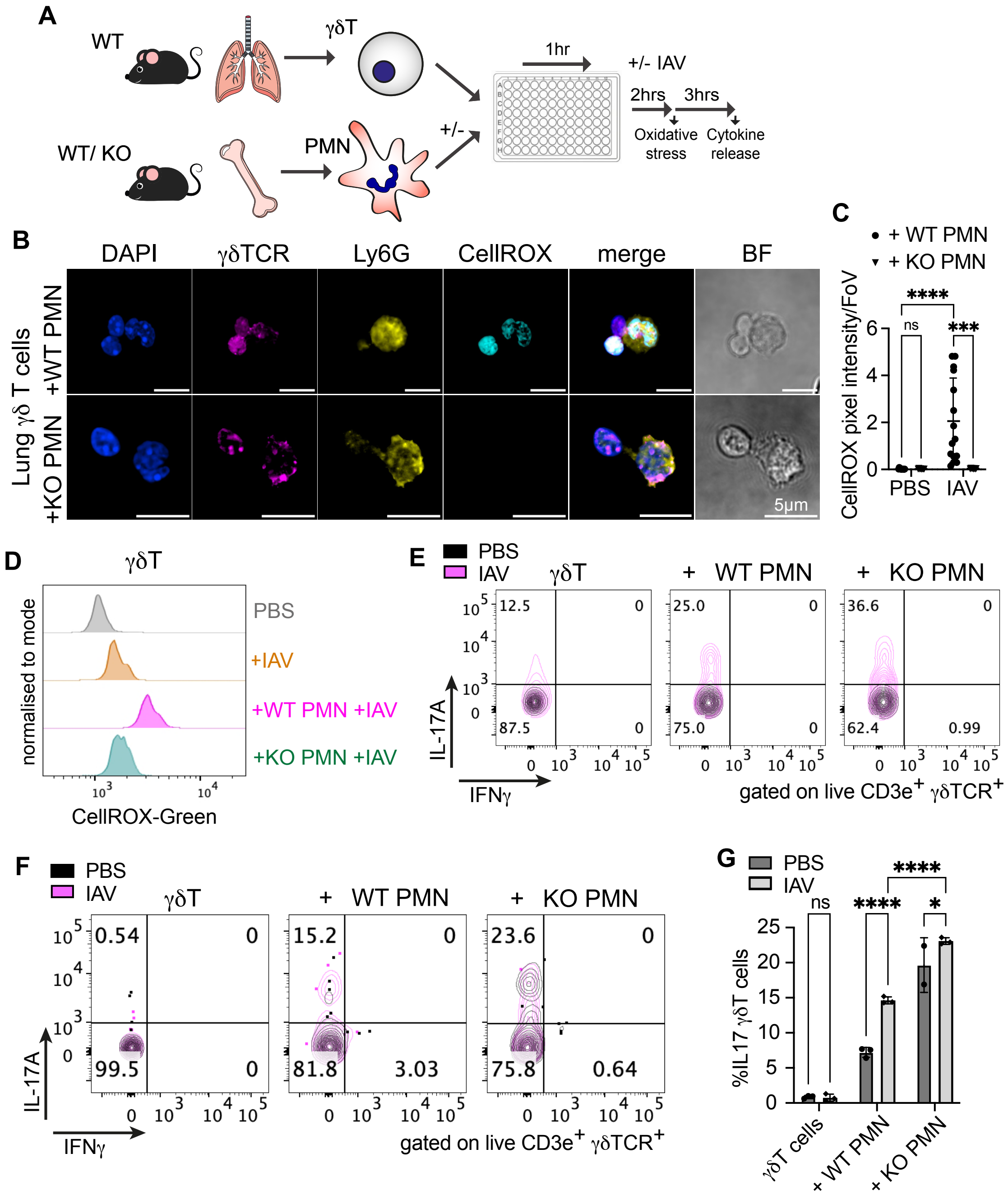

Supplementary Figure 14. Mouse neutrophil and γδ T cell co-cultures.

Isolated γδ T cells gated as Live CD45$^+$, CD3e$^+$, CD4$^-$ and γδTCR$^+$ were co-cultured *ex vivo* with WT (C57Bl/6) or KO (*Nox2*$^{-/-}$) neutrophils gated as Live CD45$^+$, CD11b$^+$ and Ly6G$^+$ were treated with PBS or infected with IAV strain PR8 at 0.2 MOI (multiplicity of infection).

A) Schematic of experimental setup. γδ T cells were isolated from lungs of C57Bl/6J (wild-type) mice and cultured alone or with bone marrow-derived neutrophils from wild-type or Nox2$^{-/-}$ mice. After 1 hour of co-culture, cells were infected with IAV or mock-treated (PBS). Oxidative stress (CellROX staining) was assessed 2 hours post-infection; cytokine release in cell culture supernatant was measured 5 hours post-infection

B - C) Representative micrographs of *ex vivo* co-cultures 2 hours post-infection with IAV stained for Oxidative stress (CellROX, cyan), neutrophils (Ly6G, magenta), γδ T cells (γδTCR, yellow) and nuclei (DAPI, blue). Scale bar 5 μm (B). CellROX pixel intensity per FoV quantitated in (C, n = 15).

D) Representative histogram plot of CellROX$^+$ γδ T cells 2 hours post-infection with IAV or PBS cultured alone, in contact with WT or KO neutrophils. Related to Figure 4E.

E - F) Representative flow cytometry plots of IL-17A$^+$/ IFNγ$^+$ γδ T cells isolated from bone marrow (E) or lung (F) gated as Live CD45$^+$, CD3e$^+$, CD4$^-$ and γδTCR$^+$ 5 hours post-infection with IAV (magenta) or PBS (black), cultured alone and in contact with WT or KO neutrophils. Related to Figure 4G – H.

G) Frequency of IL-17A$^+$ γδ T cells 5 hours post-infection cultured alone, in contact with WT or KO neutrophils (n = 3).

Bars represent mean ± SD of 2 – 3 independent experiments. p values are indicated, *<0.05, ***<0.001, ****<0.0001, ns not significant. p values were determined using two-way ANOVA and Tukey's post-test (C, G). Source data are provided as a Source Data file.

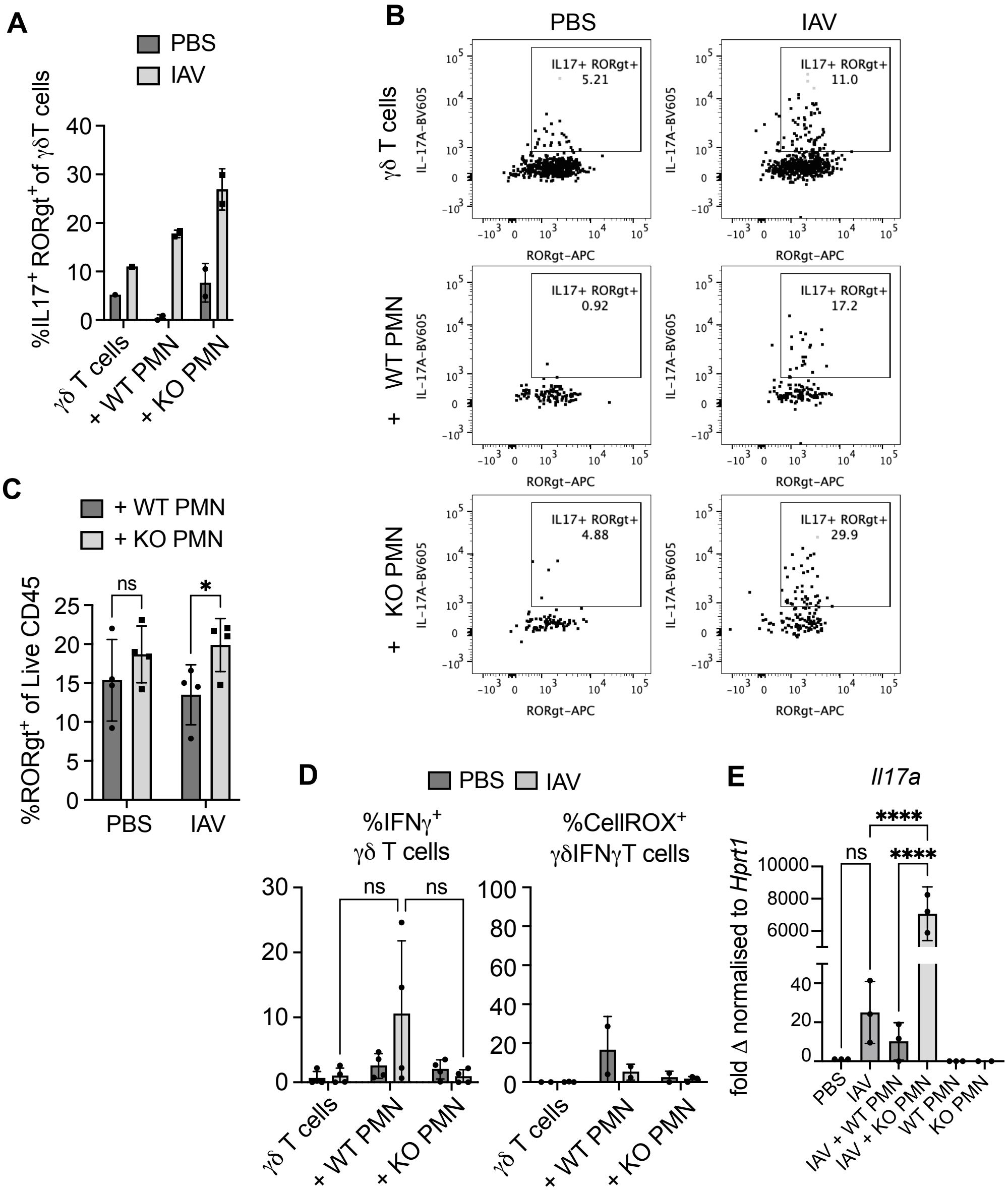

Supplementary Figure 15. Mouse neutrophil and γδ T cell co-cultures.

Isolated γδ T cells gated as Live CD45⁺, CD3e⁺, CD4⁻ and γδTCR⁺ were co-cultured *ex vivo* with WT (C57Bl/6) or KO (*Nox2*$^{-/-}$) neutrophils gated as Live CD45⁺, CD11b⁺ and Ly6G⁺ were treated with PBS or infected with IAV strain PR8 at 0.2 MOI (multiplicity of infection).

A - B) Frequency (A) and representative flow cytometry plots (B) of IL-17A⁺/RORγt⁺ γδ T cells 5 hours post-infection cultured alone, in contact with WT or KO neutrophils (n = 2).

C) Total Frequency of RORγt⁺ cells of Live CD45⁺ in coculture with WT or KO neutrophils 5 hours post-infection (n = 4).

D) Frequency of IFNγ⁺ γδ T cells (left panel, n = 4) and CellROX⁺ γδ $^{IFN\gamma}$ T cells (right panel, n = 2) 5 hours post-infection cultured alone, in contact with WT or KO neutrophils.

E) *Il17a* mRNA expression of γδ T cells, WT and KO neutrophils alone, γδ T cells co-cultured with WT or KO neutrophils assessed by RT-qPCR 4 hours post-infection (n = 3).

Bars represent mean ± SD of 2 - 4 independent experiments (C, D). Bars represent mean ± SD of technical replicates representative of 2 independent experiments (A, E). p values are indicated, *<0.05, ****<0.0001, ns not significant. p values were determined using two-way ANOVA with Tukey's post-test (C - E). Source data are provided as a Source Data file.

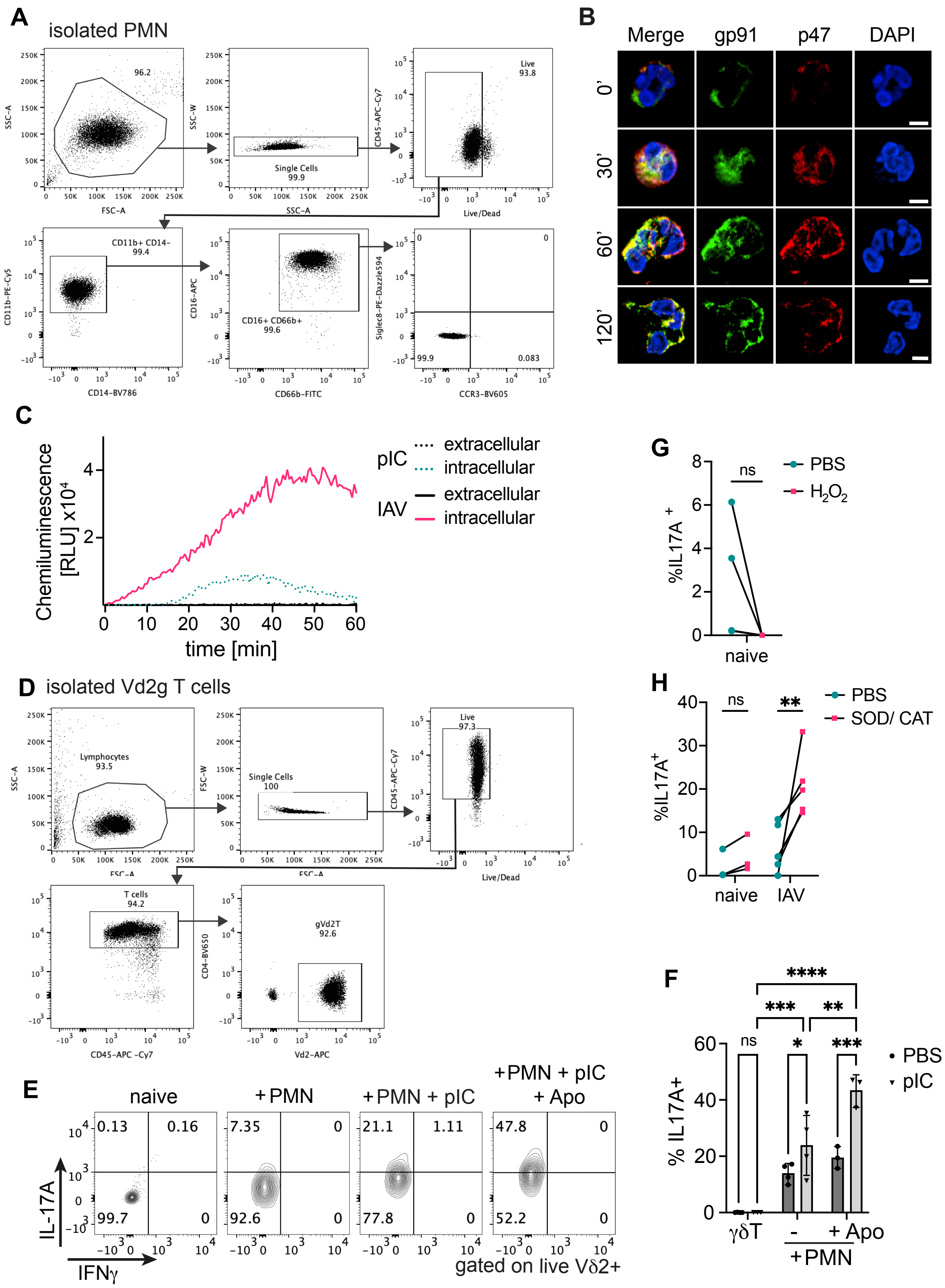

Supplementary Figure 16. Human neutrophil and γδ T cell co-cultures.

γδ T cells and neutrophils isolated from peripheral blood of healthy volunteers were co-cultured *ex vivo*.

A) Gating strategy and representative flow cytometry plots of isolated neutrophils gated as Live CD45RA$^-$, CD11b$^+$, CD14$^-$, CD16$^+$, CD66b$^+$, CCR3$^-$ and Siglec-8$^-$ cells. Related to Figure 5.

B) Representative micrographs of isolated human neutrophils at indicated time points (minutes) post-stimulation with IAV PR8 at 0.2 MOI stained for $p47^{phox}$ (red), $gp91^{phox}$ (green) and nuclei (DAPI, blue). Scale bar 5 µm. Related to Figure 5C.

C) ROS production by human blood-derived neutrophils stimulated with 1 µg/ml poly(I:C) (pIC) and IAV PR8 at 0.2 MOI detected by chemiluminescence of cell-impermeable isoluminol to detect extracellular ROS or by the cell-permeable luminol to detect intra- and extracellular ROS, in the presence of exogenous horseradish peroxidase to detect superoxide production. Representative of 2 independent experiments.

D) Gating strategy and representative flow cytometry plots of isolated γδ T cells gated as Live $CD45^+$, $CD3^+$, $CD4^-$ and $\gamma V\delta 2TCR^+$ cells. Related to Figure 5.

E) Representative flow cytometry plots of IL-$17A^+$/ $IFN\gamma^+$ γδ T cells gated as Live $CD45^+$, $CD3^+$, $CD4^-$ and $\gamma V\delta 2TCR^+$ pre-treated 1 hour with PBS or 300 µM Apocynin (Apo) cultured alone, in co-culture with unstimulated or pIC-stimulated neutrophils for 5 hours.

F) Frequency of IL-$17A^+$ γδ T cells pre-treated 1 hour with PBS or 300 µM Apocynin (Apo) cultured alone, in co-culture with unstimulated or pIC-stimulated neutrophils for 5 hours, gated as Live $CD45^+$, $CD3^+$, $CD4^-$ and $\gamma V\delta 2TCR^+$ cells (n = 4 without, n = 3 with Apocynin).

G) Frequency of IL-$17A^+$ γδ T cells 5 hours post addition of 500 µM hydrogen peroxide H_2O_2. Each data point represents one donor. Values from same donor are indicated (n = 3).

H) Frequency of IL-$17A^+$ γδ T cells pretreated with PBS or 700 U/ml Superoxide dismutase in combination with 5000 U/ml Catalase, 5 hours post infection with IAV PR8 at MOI 0.2. Each data point represents one donor. Values from same donor are indicated (naïve: n = 3, IAV: n = 5).

Bars represent mean ± SD of 3 - 5 independent experiments. p values are indicated, *<0.05, **<0.01, ***<0.001, ****<0.0001, ns not significant. p values were determined using two-way ANOVA with Tukey's post-test (F), Wilcoxon matched pairs signed rank test (G) and two-way ANOVA with Uncorrected Fisher's LSD test (H). Source data are provided as a Source Data file.

Resources table

Reagent or Resource	Source and concentration/ dilution	Identifier (Cat.no., RRID)
**Antibodies**		
Anti-goat IgG Alexa Fluor 488	Invitrogen, f.c. 5μg/ml	Cat# A11055, RRID: AB2534102
Anti-hamster IgG Alexa Fluor 647	Invitrogen, f.c. 5μg/ml	Cat# A-21451, RRID: AB2535868
Anti-mouse IgG Alexa Fluor 568	Invitrogen, f.c. 5μg/ml	Cat# A10037, RRID: AB11180865
Anti-rabbit IgG, Alexa 568	Invitrogen, f.c. 5μg/ml	Cat# A10042, RRID: AB2534017
Anti-human CD3-PE-Cy5 (clone OKT3)	Biolegend, 1:200	Cat# 317355, RRID: AB2904343
Anti-human CD4 BV650 (clone RPA-T4)	Biolegend, 1:100	Cat# 300535, RRID: AB2632791
Anti-human CD8a BV785 (clone RPA-T8)	Biolegend, 1:400	Cat# 301045, RRID: AB2563264
Anti-human CD11b PE-Cy5 (clone ICRF44)	Biolegend, 1:200	Cat# 301308, RRID: AB314160
Anti-human CD14 BV786 (clone M5E2)	Biolegend, 1:400	Cat# 301840, RRID: AB2563425
Anti-human CD16 PE (clone 3G8)	Biolegend, 1:400	Cat# 302056, RRID: AB314207
Anti-human CD45 APC-Cy7 (clone HI30)	Biolegend, 1:200	Cat# 304014, RRID: AB314402
Anti-human CD193/ CCR3 BV605 (clone 5E8)	Biolegend, 1:200	Cat# 310715, RRID: AB2562548
Anti-human Siglec-8 PE/Dazzle 594 (clone 7C9)	Biolegend, 1:200	Cat# 347109, RRID: AB2629717
Anti-human IL-1β Alexa Fluor 647 (clone H1b-98)	Biolegend, 1:50	Cat# 511707, RRID: AB2124352
Anti-human IL-17A BV605 (clone BL168)	Biolegend, 1:50	Cat# 512326, RRID: AB2563887
Anti-human IFNγ BV711 (clone B27)	BD Bioscience, 1:50	Cat# 564039, RRID: AB2738557
Anti-human TCR Vδ2 APC (B6)	Biolegend, 1:50	Cat# 331418, RRID: AB2687323
Anti-Influenza A nucleoprotein (NP)	Invitrogen, f.c. 4.8μg/ml	Cat# PA5-32242, RRID: AB2549715
Anti-mouse CD1d BV421 (clone 1B1)	Biolegend, 1:500	Cat# 123527, RRID: AB2800592
Anti-mouse CD3e PE-Cy5 (clone 145-2C11)	Biolegend, 1:200	Cat# 100310, RRID: AB312674

Anti-mouse CD4 BV650 (clone RM4-5)	Biolegend, 1:300	Cat# 100546, RRID: AB2562098
Anti-mouse CD8a PE-Cy7 (clone 53-6.7)	Biolegend, 1:500	Cat# 100722, RRID: AB312746
Anti-mouse CD11a PerCP-Cy5.5 (clone 2D7)	Biolegend, 1:200	Cat# 162908, RRID: AB3097463
Anti-mouse CD11b APC (clone M1/70)	Biolegend, 1:1000	Cat# 101212, RRID: AB312795
Anti-mouse CD11c PE-Cy7 (clone N418)	Biolegend, 1:500	Cat# 117317, RRID: AB493569
Anti-mouse CD19 BV650 (clone 6D5)	Biolegend, 1:500	Cat# 115541, RRID: AB11204087
Anti-mouse CD25 BV605 (clone PC61)	Biolegend, 1:100	Cat# 102035, RRID: AB11126977
Anti-mouse/human CD27 PE/Dazzle 594 (clone LG.3A10)	Biolegend, 1:500	Cat# 124228, RRID: AB2565794
Anti-mouse CD44 BV605 (clone IM7)	Biolegend, 1:200	Cat# 103047, RRID: AB2562451
Anti-mouse CD45 APC-Cy7 (clone 30-F11)	Biolegend, 1:1500	Cat# 103116, RRID: AB312981
Anti-mouse CD49d FITC (clone R1-2)	Biolegend, 1:500	Cat# 103605, RRID: AB313036
Anti-mouse CD62L PE/Dazzle 594 (clone MEL-14)	Biolegend, 1:200	Cat# 104447, RRID: AB2566162
Anti-mouse CD63 PE/Dazzle 594 (clone NVG-2)	Biolegend, 1:200	Cat# 143914, RRID: AB2565504
Anti-mouse CD64 BV421 (clone X54-5/7.1)	Biolegend, 1:500	Cat# 139309, RRID: AB2562694
Anti-mouse CD71 BV421 (clone RI7217)	Biolegend, 1:100	Cat# 113813, RRID: AB10899739
Anti-mouse CD98 PerCP-Cy5.5 (clone RL388)	Biolegend, 1:100	Cat# 128217, RRID: AB2783081
Anti-mouse CD103 BV711 (clone 2E7)	Biolegend, 1:500	Cat# 121435, RRID: AB2686970
Anti-mouse CD170 BV421 (clone S17007L)	Biolegend, 1:500	Cat# 155509, RRID: AB2810421
Anti-mouse CD182/ CXCR2 APC (clone SA044G4)	Biolegend, 1:200	Cat# 149311, RRID: AB2728184
Anti-mouse CD183/ CXCR3 PerCP-Cy5.5 (clone CXCR3-173)	Biolegend, 1:200	Cat# 126513, RRID: AB1186017
Anti-mouse CD192/ CCR2 BV785 (clone SA203G11)	Biolegend, 1:200	Cat# 150621, RRID: AB2721565

Anti-mouse CD193/ CCR3 APC (clone J073E5)	Biolegend, 1:200	Cat# 144511, RRID: AB2565737
Anti-mouse CD196/ CCR6 BV785 (clone 29-2L17)	Biolegend, 1:200	Cat# 129823, RRID: AB2715923
Anti-mouse CD274/ PD-L1 BV785 (clone 10F.9G2)	Biolegend, 1:200	Cat# 124331, RRID: AB2629659
Anti-mouse/human Ki-67 BV421 (clone 11F6)	Biolegend, 1:50	Cat# 151208, RRID: AB2629748
Anti-mouse I-A/I-E PE-Cy5 (clone M5/114.15.2)	Biolegend, 1:1000	Cat# 107611, RRID: AB313326
Anti-mouse IL-1β DyLight 488 (clone B122)	Universal Biologicals Ltd, 1:50	Cat# I-960, RRID: AB28310
Anti-mouse IL-17A BV605 (clone TC11-18H10.1)	Biolegend, 1:50	Cat# 506927, RRID: AB11126144
Anti-mouse IFN-γ BV711 (clone XMG1.2)	Biolegend, 1:50	Cat# 505836, RRID: AB2650928
Anti-mouse Ly-6C Pacific Blue (clone HK1.4)	Biolegend, 1:1500	Cat# 128013, RRID: AB1732090
Anti-mouse Ly-6G PE (clone 1A8)	Biolegend, 1:1000	Cat# 127608, RRID: AB1186099
Anti-mouse Ly-6G Alexa Fluor 647 (clone 1A8)	Biolegend, f.c. 5µg/ml	Cat# 127610, RRID: AB1186099
Anti-mouse NK1.1 Pacific Blue (clone PK136)	Biolegend, 1:500	Cat# 108721, RRID: AB2234352
Anti-mouse Rorγt APC (clone 1181A)	R&D, 10µl/10^6 cells	Cat# IC9125A-025
Anti-mouse TCR γ/δ APC (clone GL3)	Biolegend, 1:500	Cat# 118116, RRID: AB1731813
Anti-mouse TCR Vγ 1.1 APC (clone 2.11)	Biolegend, 1:500	Cat# 141107, RRID: AB1089780
Anti-p47^{phox} (clone D-10)	Santa Cruz, 1:200	Cat# sc-17845, RRID: AB627986
Anti-gp91^{phox} Alexa Fluor 488 (clone 54.1)	Santa Cruz, 1:200	Cat# sc-130543, RRID: AB2261483
Anti-Cybb FITC	Cusabio, 1:120	Cat# PA006325LC01HU
Anti-Nox2/ gp91^{phox}	GeneTex, 0.5µg/ml	Cat# GTX12024
InVivoPlus Anti-mouse IL-1β (clone B122)	BioXcell, 500µg/ml	Cat# BE0246, RRID: AB2687727
InVivoPlus Anti-mouse IL-17A (clone 17F3)	BioXcell, 500µg/ml	Cat# BE0173, RRID: AB10950102
InVivoPlus Anti-mouse Ly6G (clone 1A8)	BioXcell, 750µg/ml	Cat# BE0075, RRID: AB1107721
InVivoPlus polyclonal Armenian hamster IgG	BioXcell, 500µg/ml	Cat# BE0091, RRID: AB1107773

InVivoPlus mouse IgG1 isotype control (clone MOPC-21)	BioXcell, 500μg/ml	Cat# BE0083, RRID: AB1107784
InVivoPlus rat IgG2a isotype control (clone 2A3)	BioXcell, 750μg/ml	Cat# BE0089, RRID: AB1107769
Human TruStain FcX	Biolegend, 5μl/10^6 cells	Cat# 422302, RRID: AB2818986
TruStain FcX (Anti-mouse CD16/32, clone 93)	Biolegend, 10μg/ml	Cat# 101320, RRID: AB1574975
**Virus strains**		
Influenza A/ HKx31 (X-31) H3N2	Townsend AR, Skehel JJ. Influenza A specific cytotoxic T-cell clones that do not recognize viral glycoproteins. Nature. 1982 Dec 16;300(5893):655-7. doi: 10.1038/300655a0. PMID: 6183597.	
Influenza A/ Puerto Rico/ 8/ 34 (PR8) H1N1	Francis T Jr. Transmission of influenza by a filterable virus. *Science* 1934 Nov 16;80(2081):457-9. doi: 10.1126/science.80.2081.457-a. PMID: 17795179.	
SARS-CoV2 England 02/2020/407073	Lillie PJ, Samson A, Li A, Adams K, Capstick R, Barlow GD, Easom N, Hamilton E, Moss PJ, Evans A, Ivan M, Phe Incident Team, Taha Y, Duncan CJA, Schmid ML, The Airborne Hcid Network. Novel coronavirus disease (Covid-19): The first two patients in the UK with person to person transmission. J Infect. 2020 May;80(5):578-606. doi: 10.1016/j.jinf.2020.02.020. Epub 2020 Feb 28. PMID: 32119884; PMCID: PMC7127394.	
**Chemicals, reagents, peptides and recombinant proteins**		
Ammonium-chloride-potassium (ACK) buffer	Gibco	Cat# A104920
Animal-Free Blocker and Diluent	2B Scientific	Cat# SP-5035-100
Apocynin	Merck	Cat# A10809
Apotracker Green	Biolegend	Cat# 427402
LIVE/DEAD Fixable Aqua Dead Cell Stain Kit	Invitrogen	Cat# L34957
Bicinchoninic acid (BCA) protein assay kit	Pierce	Cat# 23227
Bovine serum albumin (BSA)	Thermo Fisher	Cat# BP1600-100
Brefeldin	Biolegend	Cat# 420601
Catalase from bovine liver	Merck	Cat# C30
CBA Mouse/Rat Soluble Protein Master Buffer Kit	BD Bioscience	Cat# 558266
Cell activation cocktail	Biolegend	Cat# 423302
CellROX Green Reagent, for oxidative stress detection	Invitrogen	Cat# C10444
Complete Mini protease inhibitor cocktail	Roche	Cat# 1836153001

Complexed Polyinosinic:polycytidylic acid (Poly-IC)	Invivo Gen	Cat# tlrl-picwlv
Crystal Violet	Sigma	Cat# C0775
Fixation buffer	Biolegend	Cat# 420801
Cytoperm/Cytofix buffer	BD Bioscience	Cat# 554714
Dihydroethidium Reagent (DHE)	Invitrogen	Cat# D11347
Diphenyleneiodonium chloride (DPI)	Cambridge Bioscience	Cat# 81050
Dulbecco's modified Eagle medium (DMEM)	Gibco	Cat# 10564011
EasySep human γ/δ T cell isolation kit	Stemcell Technologies	Cat# 19255
EasySep Mouse Neutrophil enrichment kit	Stemcell Technologies	Cat# 19762
EasySep Release Mouse APC positive selection kit	Stemcell Technologies	Cat# 100-0033
Enhanced Chemiluminescence (ECL) substrate	Pierce	Cat# 35055
Epoxomicin	Generon	Cat# A2606
Fetal bovine serum (FBS)	Cytiva Hyclone	Cat# 12694237
FlowCellect oxidative stress kit	Merck	Cat# FCCH025111
Fixation/Permeabilisation kit	BD Bioscience	Cat# 554714
GlutaMAX Supplement	Gibco	Cat# 35050061
Hank's Balanced Salt solution (HBSS) with Ca^{2+} and Mg^{2+}	Gibco	Cat# 14025092
Hank's Balanced Salt solution (HBSS) without Ca^{2+} and Mg^{2+}	Gibco	Cat# 14170112
HEPES 4-(2-hydroxyethyl)-1-piperazineethanesulfonic acid	Gibco	Cat# 15630056
Histopaque 1119	Merck	Cat# 11191-100ML
Human IL-1β Uncoated ELISA kit	Invitrogen	Cat# 88-7261-88
Horse radish peroxidase HRP	Merck	Cat# 1162160001
Image-iT fixative solution	Invitrogen	Cat# FB002
Iscove's Modified Dulbecco's Medium (IMDM)	Gibco	Cat# 12440053
Isoluminol	Sigma	Cat# A8264-1G
Liberase TL	Roche	Cat# 5401020001
Luminol	Sigma	Cat# 123072-2.5G

Minimum essential medium (MEM)	Gibco	Cat# 41090028
Monensin	Biolegend	Cat# 420701
Mouse IFNα ELISA kit	Invitrogen	Cat# BMS6027TWO
Mouse IFNβ ELISA kit	PBL	Cat# 42410-1
Mouse IFNγ CBA Flex set A4	BD Bioscience	Cat# 558296
Mouse IFNγ ELISA kit	Invitrogen	Cat# 88-7314-22
Mouse IFNλ ELISA kit	Biotechne	Cat# DY1789B-05
Mouse IL-1β CBA Flex set E5	BD Bioscience	Cat# 560232
Mouse IL-1β Uncoated ELISA kit	Invitrogen	Cat# 88-7013-22
Mouse IL-17A CBA Flex set C5	BD Bioscience	Cat# 560283
Mouse IL-17A Uncoated ELISA Kit	Invitrogen	Cat# 88-7371-22
Mouse IL-17F CBA Flex set D6	BD Bioscience	Cat# 562174
Mouse IL-6 CBA Flex Set B4	BD Bioscience	Cat# 558301
Mouse KC CBA Flex set A9	BD Bioscience	Cat# 558340
Mouse MCP-1 CBA Flex set B7	BD Bioscience	Cat# 558342
Mouse PGE2 ELISA kit	LSBio	Cat# LS-F32354-LSP
Mouse TNF CBA Flex set C8	BD Bioscience	Cat# 558299
Mouse TNF ELISA kit	Stemcell Technologies	Cat# 2030
N-Ethylmaleimide	Cambridge Bioscience	Cat# HY-D0843
Nuclear Extraction kit	Cambridge Bioscience	Cat# 10009277
NuPAGE 4-12% Bis Tris protein gel	Invitrogen	Cat# 10247002
Optimal cutting temperature (OCT) medium	VWR	Cat# 361603E
Oxyburst Green H2DCFDA	Invitrogen	Cat# D-2935
Paraformaldehyde 4% (PFA)	VWR	Cat# 9713
Phosphate-buffered saline (PBS)	Gibco	Cat# 10010023
PCRmax Human Influenza Type A M1 Kit	Cole Parmer	Cat# PKIT10035
Percoll	GE Healthcare	Cat# 17544502
Poly-D-lysine BioCoat	Corning	Cat# 354210
ProLong Diamond Antifade Mountant	Invitrogen	Cat# P36970
Proteome profiler mouse XL cytokine array	R&D	Cat# ARY028
Recombinant human TNF	Abcam	Cat# ab259410
RNAlater Stabilisation solution	Invitrogen	Cat# AM7020

TaqMan RNA-to-C_T *1-Step* Kit	Applied Biosystems	Cat# 10158014
RNeasy kit	Qiagen	Cat# 74104
Sucrose	Sigma	Cat# S9378
Superoxide dismutase	Merck	Cat# S5395
TaqMan Universal Master Mix II, no UNG	Applied Biosystems	Cat# 4440047
TransAM NFκB p65 assay kit	Active Motif	Cat# 40096
Transcriptor Frist Strand cDNA synthesis kit	Roche	Cat# 4379012001
TPCK-treated Trypsin	2BScientific	Cat# 21560034-1
TRI Reagent Solution	Invitrogen	Cat# AM9738
Zombie Violet	Biolegend	Cat# 423113
4',6-diamidino-2-phenylindole (DAPI)	Insight Biotechnologies	Cat# AR1176
**Experimental models: Cell lines**		
Madin-Darby canine kidney (MDCK) cell line (NBL-2)	Sigma	Cat# 00062106, RRID: CVCL_0422
**Experimental models: Organism**		
Mouse: C57BL/6-Ly6G(tm2621(Cre-tdTomato)Arte; Ai14	Matthias Gunzer (University Duisburg-Essen)	Hasenberg et al. Nat Methods 2015 doi: 10.1038/nmeth.3322. Epub 2015 Mar 16. PMID: 25775045.
Mouse: Nox2 fl/fl	Ajay Shah (King's College London)	Sag et al. Circulation 2017 doi: 10.1161/CIRCULATIONAHA.116.023877. PMID:28298457.
Mouse: Nox2 KO, B6.129S-Cybbtm1Din/J	Mary Dinauer (University St. Louis)	Pollock et al. Nat Genet 1995 https://doi.org/10.1038/ng0295-202 IMSR_JAX:002365
Mouse: C57BL/6J	Jackson Laboratory	IMSR_JAX:000664
**Gene expression assays**		
Human HPRT1	Applied Biosystems	Hs02800695_m1
Human IL-1β	Applied Biosystems	Hs01555410_m1
Mouse B2M	Applied Biosystems	Mm00437762_m1
Mouse HPRT1	Applied Biosystems	Mm03024075_m1
Mouse IFIT3	Applied Biosystems	Mm01704846_s1
Mouse IFNα4	Applied Biosystems	Mm00833969_s1
Mouse IFNβ1	Applied Biosystems	Mm00439552_s1
Mouse IFNγ	Applied Biosystems	Mm01168134_m1
Mouse IFNλ3	Applied Biosystems	Mm00663660_g1
Mouse IL-1β	Applied Biosystems	Mm00434228_m1
Mouse IL-17A	Applied Biosystems	Mm00439618_m1
Mouse ISG15	Applied Biosystems	Mm01705338_s1
Mouse OAS	Applied Biosystems	Mm00836412_m1
Mouse Rsad2	Applied Biosystems	Mm00491265_m1

Mouse Stat1	Applied Biosystems	Mm01257286_m1
Mouse TNF	Applied Biosystems	Mm00443258_m1
Mouse Cybb	Applied Biosystems	Mm01287743_m1
**Software and Algorithms**		
GraphPad Prism v10	GraphPad by Dotmatics	https://www.graphpad.com/
FlowJo Version v10.9	Becton, Dickinson and Company	https://www.flowjo.com/
ImageJ 1.54p	Schneider et al., 2012/ NIH	https://imagej.org
CellProfiler 4.2.8	Broad Institute, Inc.	https://cellprofiler.org
Jupyterlab v3.6.7	Project Jupyter	Jupyter.org
Python 3.9.19	Python software foundation	
