# Supplementary material for: Neutrophil NADPH oxidase breaks the inflammatory IL-1β/IL-17A circuit to enhance pathogen clearance during respiratory virus infections
Source: Nat Commun. 2026 Aug 5;17:9425. doi: 10.1038/s41467-026-76327-4 (PMC13538623; doi:10.1038/s41467-026-76327-4)
Supplement: Supplementary file 2 [file 41467_2026_76327_MOESM2_ESM.pdf]

nature portfolio

Corresponding author(s): Annika Warnatsch

Last updated by author(s): 16/7/2026

Reporting Summary

Nature Portfolio wishes to improve the reproducibility of the work that we publish. This form provides structure for consistency and transparency in reporting. For further information on Nature Portfolio policies, see our Editorial Policies and the Editorial Policy Checklist.

Statistics

For all statistical analyses, confirm that the following items are present in the figure legend, table legend, main text, or Methods section.

n/a	Confirmed	
☐	☒	The exact sample size (*n*) for each experimental group/condition, given as a discrete number and unit of measurement
☐	☒	A statement on whether measurements were taken from distinct samples or whether the same sample was measured repeatedly
☐	☒	The statistical test(s) used AND whether they are one- or two-sided<br>*Only common tests should be described solely by name; describe more complex techniques in the Methods section.*
☒	☐	A description of all covariates tested
☐	☒	A description of any assumptions or corrections, such as tests of normality and adjustment for multiple comparisons
☐	☒	A full description of the statistical parameters including central tendency (e.g. means) or other basic estimates (e.g. regression coefficient) AND variation (e.g. standard deviation) or associated estimates of uncertainty (e.g. confidence intervals)
☐	☒	For null hypothesis testing, the test statistic (e.g. *F*, *t*, *r*) with confidence intervals, effect sizes, degrees of freedom and *P* value noted<br>*Give P values as exact values whenever suitable.*
☒	☐	For Bayesian analysis, information on the choice of priors and Markov chain Monte Carlo settings
☒	☐	For hierarchical and complex designs, identification of the appropriate level for tests and full reporting of outcomes
☐	☒	Estimates of effect sizes (e.g. Cohen's *d*, Pearson's *r*), indicating how they were calculated

Our web collection on statistics for biologists contains articles on many of the points above.

Software and code

Policy information about availability of computer code

Data collection	Flow cytometry data were acquired using an LSRFortessa or Cytoflex cell analyser, and FACS Aria cell sorter (all BD Biosciences). Microscopy images were captured on a Nikon A1R or AXR/NSPARC confocal microscope with spectral detector. Gene expression data were obtained using a Quant Studio 7 (Applied Biosystems), histological images were scanned using a NanoZoomer slide scanner (Hamamatsu), and plate assay data with chemiluminescence/absorbance readouts were acquired using a Spectramax plate reader (Molecular Devices).
Data analysis	Flow cytometry analysis was conducted using FlowJo v11 (BD Biosciences). Statistical analysis and graphing were performed with GraphPad Prism v10, and spreadsheet management was handled using Excel (Microsoft Office). Microscopy image analysis was completed using ImageJ 1.54f.<br>Scripts and Cellprofiler pipelines used for Image analysis can be assessed on GitHub Repository github.com/AnniWarn/Nox2.

For manuscripts utilizing custom algorithms or software that are central to the research but not yet described in published literature, software must be made available to editors and reviewers. We strongly encourage code deposition in a community repository (e.g. GitHub). See the Nature Portfolio guidelines for submitting code & software for further information.

Data

Policy information about availability of data

All manuscripts must include a data availability statement. This statement should provide the following information, where applicable:

- Accession codes, unique identifiers, or web links for publicly available datasets
- A description of any restrictions on data availability
- For clinical datasets or third party data, please ensure that the statement adheres to our policy

Source Data are provided with this paper. Further information and requests for resources and reagents should be directed to the lead contact, Annika Warnatsch (annika.warnatsch@kcl.ac.uk).

Research involving human participants, their data, or biological material

Policy information about studies with human participants or human data. See also policy information about sex, gender (identity/presentation), and sexual orientation and race, ethnicity and racism.

Reporting on sex and gender	Healthy adult donors of both sexes and genders (self-reported) participated in the study, and sex was not considered a factor in the selection of samples. The inclusion criteria for donors were 18 years of age or older and no prior history of respiratory disease.
Reporting on race, ethnicity, or other socially relevant groupings	The selection of samples for studies on primary human blood content did not take race or ethnicity into consideration.
Population characteristics	Population characteristics are reflective of healthy volunteers. Participants were 18 years of age or older and had no history of respiratory disease and were not recruited based on genotype, ethnicity, sex, or other demographic characteristics. Individuals with major medical conditions, pregnancy, anaemia, recent large-volume blood donation, or blood-borne viral infections (including HIV, hepatitis B, or hepatitis C) were excluded according to the study protocol. No genotypic information was collected, and participants were not stratified by medication use or treatment category.
Recruitment	No authors in this study participated in recruitment of volunteers. Recruitment was based on voluntary participation, introducing the possibility of self-selection bias, whereby individuals willing to participate in research may differ from the general population in health status or other unmeasured characteristics. As the study was designed to investigate fundamental neutrophil biology in healthy donors rather than estimate population-level parameters, this bias is unlikely to affect the mechanistic conclusions, although it may limit the generalisability of the findings to broader populations, including individuals with respiratory disease.
Ethics oversight	Human participants were recruited under a protocol sponsored by King's College London (co-sponsored by Guy's and St Thomas' NHS Foundation Trust) that was approved by the London Bridge Research Ethics Committee (REC reference 14/LO/1699; IRAS Project ID 147574).

Note that full information on the approval of the study protocol must also be provided in the manuscript.

Field-specific reporting

Please select the one below that is the best fit for your research. If you are not sure, read the appropriate sections before making your selection.

[x] Life sciences [ ] Behavioural & social sciences [ ] Ecological, evolutionary & environmental sciences

For a reference copy of the document with all sections, see nature.com/documents/nr-reporting-summary-flat.pdf

Life sciences study design

All studies must disclose on these points even when the disclosure is negative.

Sample size	For both in vivo and ex vivo studies, we designed experiments to use the minimal number of animals and human volunteers necessary to observe the hypothesized effects, adhering to ethical guidelines, and determined sample sizes based on power calculations to ensure statistical validity.
Data exclusions	For in vivo experiments, we confirmed successful infection by verifying a viral burden above zero by qPCR for M1 transcript and excluded any animals that were not infected.
Replication	All experiments were repeated at least twice in independent trials, as indicated in the figure legends.
Randomization	Animals and human participants were randomly allocated to experimental groups. For animal experiments littermate controls were used wherever applicable to minimize genetic variability.
Blinding	A fully informed data analysis was conducted in the study, with the exception of histopathological analysis, which was performed in a blinded manner.

Reporting for specific materials, systems and methods

We require information from authors about some types of materials, experimental systems and methods used in many studies. Here, indicate whether each material, system or method listed is relevant to your study. If you are not sure if a list item applies to your research, read the appropriate section before selecting a response.

Materials & experimental systems

n/a	Involved in the study	
	x	Antibodies
	x	Eukaryotic cell lines
x		Palaeontology and archaeology
	x	Animals and other organisms
x		Clinical data
x		Dual use research of concern
x		Plants

Methods

n/a	Involved in the study	
x		ChIP-seq
	x	Flow cytometry
x		MRI-based neuroimaging

Antibodies

Antibodies used

Anti-goat IgG Alexa Fluor 488 Invitrogen, f.c. 5μg/ml Cat# A11055, RRID: AB2534102
Anti-hamster IgG Alexa Fluor 647 Invitrogen, f.c. 5μg/ml Cat# A-21451, RRID: AB2535868
Anti-mouse IgG Alexa Fluor 568 Invitrogen, f.c. 5μg/ml Cat# A10037, RRID: AB11180865
Anti-rabbit IgG, Alexa 568 Invitrogen, f.c. 5μg/ml Cat# A10042, RRID: AB2534017
Anti-human CD3-PE-Cy5 (clone OKT3) Biolegend, 1:200 Cat# 317355, RRID: AB2904343
Anti-human CD4 BV650 (clone RPA-T4) Biolegend, 1:100 Cat# 300535, RRID: AB2632791
Anti-human CD8a BV785 (clone RPA-T8) Biolegend, 1:400 Cat# 301045, RRID: AB2563264
Anti-human CD11b PE-Cy5 (clone ICRF44) Biolegend, 1:200 Cat# 301308, RRID: AB314160
Anti-human CD14 BV786 (clone M5E2) Biolegend, 1:400 Cat# 301840, RRID: AB2563425
Anti-human CD16 PE (clone 3G8) Biolegend, 1:400 Cat# 302056, RRID: AB314207
Anti-human CD45 APC-Cy7 (clone HI30) Biolegend, 1:200 Cat# 304014, RRID: AB314402
Anti-human CD193/ CCR3 BV605 (clone 5E8) Biolegend, 1:200 Cat# 310715, RRID: AB2562548
Anti-human Siglec-8 PE/Dazzle 594 (clone 7C9) Biolegend, 1:200 Cat# 347109, RRID: AB2629717
Anti-human IL-1β Alexa Fluor 647 (clone H1b-98) Biolegend, 1:50 Cat# 511707, RRID: AB2124352
Anti-human IL-17A BV605 (clone BL168) Biolegend, 1:50 Cat# 512326, RRID: AB2563887
Anti-human IFNγ BV711 (clone B27) BD Bioscience, 1:50 Cat# 564039, RRID: AB2738557
Anti-human TCR Vδ2 APC (B6) Biolegend, 1:50 Cat# 331418, RRID: AB2687323
Anti-Influenza A nucleoprotein (NP) Invitrogen, f.c. 4.8μg/ml Cat# PA5-32242, RRID: AB2549715
Anti-mouse CD1d BV421 (clone 1B1) Biolegend, 1:500 Cat# 123527, RRID: AB2800592
Anti-mouse CD3e PE-Cy5 (clone 145-2C11) Biolegend, 1:200 Cat# 100310, RRID: AB312674
Anti-mouse CD4 BV650 (clone RM4-5) Biolegend, 1:300 Cat# 100546, RRID: AB2562098
Anti-mouse CD8a PE-Cy7 (clone 53-6.7) Biolegend, 1:500 Cat# 100722, RRID: AB312746
Anti-mouse CD11a PerCP-Cy5.5 (clone 2D7) Biolegend, 1:200 Cat# 162908, RRID: AB3097463
Anti-mouse CD11b APC (clone M1/70) Biolegend, 1:1000 Cat# 101212, RRID: AB312795
Anti-mouse CD11c PE-Cy7 (clone N418) Biolegend, 1:500 Cat# 117317, RRID: AB493569
Anti-mouse CD19 BV650 (clone 6D5) Biolegend, 1:500 Cat# 115541, RRID: AB11204087
Anti-mouse CD25 BV605 (clone PC61) Biolegend, 1:100 Cat# 102035, RRID: AB11126977
Anti-mouse/human CD27 PE/Dazzle 594 (clone LG.3A10) Biolegend, 1:500 Cat# 124228, RRID: AB2565794
Anti-mouse CD44 BV605 (clone IM7) Biolegend, 1:200 Cat# 103047, RRID: AB2562451
Anti-mouse CD45 APC-Cy7 (clone 30-F11) Biolegend, 1:1500 Cat# 103116, RRID: AB312981
Anti-mouse CD49d FITC (clone R1-2) Biolegend, 1:500 Cat# 103605, RRID: AB313036
Anti-mouse CD62L PE/Dazzle 594 (clone MEL-14) Biolegend, 1:200 Cat# 104447, RRID: AB2566162
Anti-mouse CD63 PE/Dazzle 594 (clone NVG-2) Biolegend, 1:200 Cat# 143914, RRID: AB2565504
Anti-mouse CD64 BV421 (clone X54-5/7.1) Biolegend, 1:500 Cat# 139309, RRID: AB2562694
Anti-mouse CD71 BV421 (clone RI7217) Biolegend, 1:100 Cat# 113813, RRID: AB10899739
Anti-mouse CD98 PerCP-Cy5.5 (clone RL388) Biolegend, 1:100 Cat# 128217, RRID: AB2783081
Anti-mouse CD103 BV711 (clone 2E7) Biolegend, 1:500 Cat# 121435, RRID: AB2686970
Anti-mouse CD170 BV421 (clone S17007L) Biolegend, 1:500 Cat# 155509, RRID: AB2810421
Anti-mouse CD182/ CXCR2 APC (clone SA044G4) Biolegend, 1:200 Cat# 149311, RRID: AB2728184
Anti-mouse CD183/ CXCR3 PerCP-Cy5.5 (clone CXCR3-173) Biolegend, 1:200 Cat# 126513, RRID: AB1186017
Anti-mouse CD192/ CCR2 BV785 (clone SA203G11) Biolegend, 1:200 Cat# 150621, RRID: AB2721565
Anti-mouse CD193/ CCR3 APC (clone J073E5) Biolegend, 1:200 Cat# 144511, RRID: AB2565737
Anti-mouse CD196/ CCR6 BV785 (clone 29-2L17) Biolegend, 1:200 Cat# 129823, RRID: AB2715923
Anti-mouse CD274/ PD-L1 BV785 (clone 10F.9G2) Biolegend, 1:200 Cat# 124331, RRID: AB2629659
Anti-mouse/human Ki-67 BV421 (clone 11F6) Biolegend, 1:50 Cat# 151208, RRID: AB2629748
Anti-mouse I-A/I-E PE-Cy5 (clone M5/114.15.2) Biolegend, 1:1000 Cat# 107611, RRID: AB313326

Anti-mouse IL-1β DyLight 488 (clone B122) Universal Biologicals Ltd, 1:50 Cat# I-960, RRID: AB28310
Anti-mouse IL-17A BV605 (clone TC11-18H10.1) Biolegend, 1:50 Cat# 506927, RRID: AB11126144
Anti-mouse IFN-γ BV711 (clone XMG1.2) Biolegend, 1:50 Cat# 505836, RRID: AB2650928
Anti-mouse Ly-6C Pacific Blue (clone HK1.4) Biolegend, 1:1500 Cat# 128013, RRID: AB1732090
Anti-mouse Ly-6G PE (clone 1A8) Biolegend, 1:1000 Cat# 127608, RRID: AB1186099
Anti-mouse Ly-6G Alexa Fluor 647 (clone 1A8) Biolegend, f.c. 5µg/ml Cat# 127610, RRID: AB1186099
Anti-mouse NK1.1 Pacific Blue (clone PK136) Biolegend, 1:500 Cat# 108721, RRID: AB2234352
Anti-mouse Rort APC (clone 1181A) R&D, 10µl/106 cells Cat# IC9125A-025
Anti-mouse TCR γ/δ APC (clone GL3) Biolegend, 1:500 Cat# 118116, RRID: AB1731813
Anti-mouse TCR Vγ 1.1 APC (clone 2.11) Biolegend, 1:500 Cat# 141107, RRID: AB1089780
Anti-p47phox (clone D-10) Santa Cruz, 1:200 Cat# sc-17845, RRID: AB627986
Anti-gp91phox Alexa Fluor 488 (clone 54.1) Santa Cruz, 1:200 Cat# sc-130543, RRID: AB2261483
Anti-Cybb FITC Cusabio, 1:120 Cat# PA006325LC01HU
Anti-Nox2/ gp91phox GeneTex, 0.5µg/ml Cat# GTX12024
InVivoPlus Anti-mouse IL-1β (clone B122) BioXcell, 500µg/ml Cat# BE0246, RRID: AB2687727
InVivoPlus Anti-mouse IL-17A (clone 17F3) BioXcell, 500µg/ml Cat# BE0173, RRID: AB10950102
InVivoPlus Anti-mouse Ly6G (clone 1A8) BioXcell, 750µg/ml Cat# BE0075, RRID: AB1107721
InVivoPlus polyclonal Armenian hamster IgG BioXcell, 500µg/ml Cat# BE0091, RRID: AB1107773
InVivoPlus mouse IgG1 isotype control (clone MOPC-21) BioXcell, 500µg/ml Cat# BE0083, RRID: AB1107784
InVivoPlus rat IgG2a isotype control (clone 2A3) BioXcell, 750µg/ml Cat# BE0089, RRID: AB1107769
Human TruStain FcX Biolegend, 5µl/106 cells Cat# 422302, RRID: AB2818986
TruStain FcX (Anti-mouse CD16/32, clone 93) Biolegend, 10µg/ml Cat# 101320, RRID: AB1574975

Validation

All Biolegend antibodies are quality control tested by immunofluorescent staining with flow cytometric analysis. www.biolegend.com

Anti-human IFNγ BV711 (clone B27) BD Bioscience, Cat# 564039 https://www.bdbiosciences.com/en-gb/products/reagents/flow-cytometry-reagents/research-reagents/single-color-antibodies-ruo/bv711-mouse-anti-human-ifn.564039?tab=citations_references

Anti-Influenza A nucleoprotein (NP) Invitrogen, Cat# PA5-32242 https://www.thermofisher.com/antibody/product/Influenza-A-NP-Antibody-Polyclonal/PA5-32242

Anti-mouse IL-1β DyLight 488 (clone B122) Universal Biologicals Ltd, Cat# I-960 https://www.universalbiologicals.com/anti-mouse-il-1b-dylightr-488-i-960-100ug

Anti-mouse Rorgt APC (clone 1181A) R&D, Cat# IC9125A-025 https://www.rndsystems.com/products/human-mouse-rorgammat-rorc2-nr1f3-apc-conjugated-antibody-1181a_ic9125a

Anti-p47phox (clone D-10) Santa Cruz, Cat# sc-17845 https://www.scbt.com/p/p47-phox-antibody-d-10

Anti-gp91phox Alexa Fluor 488 (clone 54.1) Santa Cruz, Cat# sc-130543 https://www.scbt.com/p/gp91-phox-antibody-54-1?srsltid=AfmBOoqp1R_VZBjcmh8sD2vwCSxKsRv8St10rbIGicB1LKzA6dQlw7Bk

Anti-Cybb FITC Cusabio, Cat# PA006325LC01HU https://www.2bscientific.com/products/cusabio/csb-pa006325lc01hu/cybb-antibody-fitc-conjugated

Anti-Nox2/ gp91phox GeneTex, Cat# GTX12024 https://www.2bscientific.com/products/genetex/gtx12024/nox2-gp91phox-antibody

InVivoPlus Anti-mouse IL-1β (clone B122) BioXcell https://bioxcell.com/invivomab-anti-mouse-rat-il-1-beta-be0246

InVivoPlus Anti-mouse IL-17A (clone 17F3) BioXcell https://bioxcell.com/invivomab-anti-mouse-rat-il-17a-be0173

InVivoPlus Anti-mouse Ly6G (clone 1A8) BioXcell https://bioxcell.com/invivomab-anti-mouse-ly6g-be0075-1?queryID=3968f342a7f26adc16442ff0032ee72c&objectID=30551&indexName=bioxcell_live_default_products

InVivoPlus polyclonal Armenian hamster IgG BioXcell https://bioxcell.com/invivomab-polyclonal-armenian-hamster-igg-be0091

InVivoPlus mouse IgG1 isotype control (clone MOPC-21) BioXcell https://bioxcell.com/invivomab-mouse-igg1-isotype-control-unknown-specificity-be0083?queryID=8fadb22befe8733323a2099105da1126&objectID=30555&indexName=bioxcell_live_default_products

InVivoPlus rat IgG2a isotype control (clone 2A3) BioXcell https://bioxcell.com/invivomab-rat-igg2a-isotype-control-anti-trinitrophenol-be0089?queryID=f80552d3d699e9e408784ebdf389785a&objectID=30560&indexName=bioxcell_live_default_products

Eukaryotic cell lines

Policy information about cell lines and Sex and Gender in Research

Cell line source(s)	Madin-Darby canine kidney (MDCK) cell line (NBL-2) from Sigma, Cat# 00062106, RRID: CVCL_0422
Authentication	This cell line is part of the European Collection of Authenticated Cell Cultures (ECACC), an international repository managed by the United Kingdom Health Security Agency (UKHSA) and has been authenticated by the supplier
Mycoplasma contamination	Cell lines tested negative for mycoplasma contamination.
Commonly misidentified lines (See ICLAC register)	n/a

Animals and other research organisms

Policy information about studies involving animals; ARRIVE guidelines recommended for reporting animal research, and Sex and Gender in Research

Laboratory animals	All mouse strains used in this study were derived from the C57BL/6J background, including Nox2-/-, Nox2 flox/flox, and Ly6GCre/tdTomato strains. Ly6G-Cre and Nox2 fl/fl mice were crossed to generate Ly6G-Cre(+/-) Nox2 fl/fl and Ly6G-Cre(-/-) Nox2 fl/fl mice. Mice aged 8 – 14 weeks were used in the experiments.
Wild animals	No wild animals were used in this study.
Reporting on sex	Animals of both sexes were used in the experiments. Based on the observed findings, the authors conclude that the results are applicable, relevant, and reproducible for both males and females.
Field-collected samples	No field collected samples were used in this study.
Ethics oversight	All animal experiments were approved by the King's College London Animal Welfare and Ethical Review Body (AWERB) and conducted under a UK Home Office Project Licence (PP2442888) in accordance with the Animals (Scientific Procedures) Act 1986.

Note that full information on the approval of the study protocol must also be provided in the manuscript.

Plants

Seed stocks	N/A
Novel plant genotypes	N/A
Authentication	N/A

Flow Cytometry

Plots

Confirm that:

- [x] The axis labels state the marker and fluorochrome used (e.g. CD4-FITC).
- [x] The axis scales are clearly visible. Include numbers along axes only for bottom left plot of group (a 'group' is an analysis of identical markers).
- [x] All plots are contour plots with outliers or pseudocolor plots.
- [x] A numerical value for number of cells or percentage (with statistics) is provided.

Methodology

Sample preparation	Primary human cells were isolated from the peripheral blood of healthy donors by gradient centrifugation or magnetic bead separation. Mouse tissues were processed into single-cell suspensions by passing through 70µm cell strainers. Lung tissue was digested in 0.4 mg/ml Liberase TL (Roche) for 45 minutes at 37°C with shaking. After erythrocyte lysis with Ammonium-chloride-potassium (ACK) buffer, cells were washed, filtered and resuspended in Iscove's modified Dulbecco's medium (IMDM) supplemented with 10% fetal calf serum (FCS).<br>For cell surface staining, 1x10E6 cells per sample were incubated with 2.5 - 5 µg/ml antibodies for 15 minutes at room temperature, washed, and fixed in Fixation buffer (Biolegend) for 20 minutes at 4°C.

	For intracellular cytokine staining, 2x10E6 cells were incubated at 37°C for 5 hours with 1x cell activation cocktail (Biolegend). Protein transport blockers 1x Brefeldin and 1x Monensin (both Biolegend) were added for the last 3 hours of incubation. Cells were fixed and permeabilised in BD Cytoperm/Cytofix buffer for 20 minutes at 4°C, blocked for 10 minutes with 10 µg/mL TruStain FcX and stained with 5 - 10 µg/ml antibody for 1 hour at room temperature in the dark.
Instrument	LSRFortessa or Cytoflex cell analyser, Aria cell sorter (all BD Biosciences)
Software	BD FACSDiva and FlowJo v11 (BD Biosciences)
Cell population abundance	Mouse neutrophils isolated from bone marrow by magnetic bead-based negative selection: 85.23 +/- 9.86 % of live CD45+ cells.<br>Mouse gd T cells isolated from spleen/ lymph nodes by magnetic bead-based positive selection: 75.5 +/-3.57 % of live CD45+ cells.<br>Mouse gd T cells isolated from lungs by magnetic bead-based positive selection: 50.3 ± 4.6% of live CD45+ cells.<br>Human neutrophils isolated by gradient centrifugation from peripheral blood: 99.1 +/- 0.4 % of live CD45+ cells.<br>Human gd Tcells isolated from peripheral blood by magnetic bead-based positive selection: 78 +/- 11.9% of live CD45+ cells.
Gating strategy	Mouse cell populations were gated as follows:<br>Neutrophils: Live CD45⁺, CD11b^hi, CD64-, Ly6G^tdTom⁺<br>BM-isolated neutrophils: Live CD45⁺, CD11b+, Ly6G+, Siglec-F-, CCR3-<br>Monocytes: Live CD45⁺, CD11b^hi, Ly6G⁻, Ly6C^hi, MHCII⁺<br>Macrophages: Live CD45⁺, CD11b⁺, CD64⁺, and MHCII⁺<br>Conventional Dendritic Cells: Live CD45⁺, CD11c⁺, CD103⁺, and MHCII⁺<br>Alveolar Macrophages: Live CD45⁺, CD11c⁺, and Siglec-F⁺<br>NK Cells: Live CD45⁺, CD3e⁻, and NK1.1⁺<br>CD8 T Cells: Live CD45⁺, CD3e⁺, γδTCR⁻, CD8⁺<br>CD4 T Cells: Live CD45⁺, CD3e⁺, γδTCR⁻, CD4⁺<br>γδ T Cells: Live CD45⁺, CD3e⁺, γδTCR⁺<br>spleen/ lung-isolated γδ T Cells: Live CD45⁺, CD3e⁺, CD4-, CD8-, γδTCR⁺<br><br>Human cell populations were gated as follows:<br>Neutrophils: Live CD45RA⁻, CD11b⁺, CD14⁻, CD16⁺, CD66b+, CCR3-, Siglec-8-<br>γδ T Cells: Live CD45RA⁺, CD3⁺, CD4⁻, γVδ2TCR⁺<br><br>Debris and doublets were excluded using FSC-A/FSC-W and SSC-A/SSC-W parameters. Fluorescence intensities above 10^3 were defined as positive.

☒ Tick this box to confirm that a figure exemplifying the gating strategy is provided in the Supplementary Information.
