# Supplementary material for: Neutrophil NADPH oxidase breaks the inflammatory IL-1β/IL-17A circuit to enhance pathogen clearance during respiratory virus infections
Source: Nat Commun. 2026 Aug 5;17:9425. doi: 10.1038/s41467-026-76327-4 (PMC13538623; doi:10.1038/s41467-026-76327-4)
Supplement: Supplementary file 3 [file 41467_2026_76327_MOESM3_ESM.pdf]

nature portfolio

Peer Review File

Neutrophil NADPH oxidase breaks the inflammatory IL-1β/IL-17A circuit to enhance pathogen clearance during respiratory virus infections.

Corresponding Author: Dr Annika Warnatsch

This file contains all reviewer reports in order by version, followed by all author rebuttals in order by version.

Version 0:

Reviewer comments:

Reviewer #1

(Remarks to the Author)
The paper on Neutrophil NADPH oxidase breaks the inflammatory IL-1β-IL-17A circuit to enhance pathogen clearance during respiratory virus infections is of interest and overall well presented.
The proposed model in Figure 6-F on neutrophil-gd T17 cell activation in IAV infected control and neutrophil deficient Nox2 KO mice is a great summary, which could serve as a graphical abstract.
A few minor issues should be considered:
1. Structure: The reading of the results is at some instances difficult due to regular switches to the supplementary data.
2. Figure 1A: Y-axis: add label ROS DHE staining.
3. Figure 1F: Add the quantitative analyses next to the immunofluorescent plates of NP and Ly6G;
4. Figure 3: Revisit the outline of the figure allowing an easy read. Fig 3G: Please explain, I am lost.
5. Figure 5: Important data on human cells: would be great to add a scheme of the experimental approach.

Reviewer #2

(Remarks to the Author)
The manuscript by Sofoluwe and colleagues investigates the role of neutrophil-specific NADPH oxidase in regulating innate response during respiratory viral infection. Abrogation of Nox2 in neutrophils dampened viral clearance due to enhanced IL-1b-dependent proliferation of IL-17A-producing gdT cells. In turn, this counteracts antiviral IFN response. Such a pathway seems to be at play using human cells. These data are of great interest and provide important insights into our understanding of the immunopathology of respiratory viral infections. However, some additional data/controls are required to convey the general message. Here are my specific comments:
- Use of Ly6g-cre mice is indeed one of the most used model to specifically investigate neutrophil biology together with Mrp9-Cre. However, a recent study demonstrated that during IAV infection about 50% of CD11b+ Ly6G+ also expressed CD64 and were actually defined as macrophages (PMID: 39093958). This should be evaluated/controlled in this study even if macrophages are not huge producers of ROS.
- It is now well established that neutrophil are functionally heterogeneous especially upon respiratory infections. A further phenotypical characterization of neutrophils would be of interest regarding Nox2 expression and IL-1b secretion.
- The increased frequency of CD3+ T cells in Ly6G-cre+/- Nox2 flox that seems to be independent of CD4+ and CD8+ T cells cannot be solely explained by the increased in gdT cells from a numerical perspective. It is known that DN abT cells can expand upon IAV infection (PMID: 39093958). The influence of neutrophilic Nox2 on this population should be explored (function, phenotype,…).
- The use of splenic and LN gdT cells may not be suitable for the co-culture experiment since most of the purified cells from these organs are CD27+ (IFNg+) (PMID: 39093958). It would be of interest to use lung gdT cells that are mainly IL-17-producing.
- It is unclear to me how Imagestream can be used to evaluate neutrophil-gdT cell interaction since it is a flow-based strategy.
- The association between IL-17 and viral loads is interesting. How is the viral load in Tcrd-/- mice reconstituted with either WT or Il17-/- gdT cells?

Minor points:
- The gating strategy for lung immune cells should be clearly stated. For instance, it is unclear why the number of alveolar macrophages increases during IAV infection since AM depletion is a hallmark of this infection.
- To compare the absolute counts with other studies, it would be of interest to show the data as total number per lung.
- “adaptive type 2 IFN-y”: this sentence should be rephrased as it is uncertain whether this IFN-y production is dependent on innate or adaptive cells.
- “In addition, an increased % of gdT cells … RORgt in co-culture with Nox2-/- neutrophils”. This sentence should be rephrased because it suggest a shift in the expression of this peculiar TF. However, the data presented in Fig.S7b solely suggest that IL-17A producing cells are RORgt. What is the frequency of total RORgt-expressing cells upon coculture.

Reviewer #3

(Remarks to the Author)
Sofoluwe et al. have investigated the role of neutrophil-derived ROS in modulating the immune response during respiratory viral infection. They used as a model influenza A virus infection in mice and found that neutrophil-specific Nox2-deficient mice exhibit heightened lung inflammation and impaired virus clearance. They further found that neutrophil Nox2-derived ROS are involved in this process by reducing IL-1b production and curbing the proliferation of IL-17-producing gamma delta (γδ) T cells. They therefore concluded that neutrophil Nox2 and ROS act as inhibitors of an IL-1b/IL-17 axis that augments pro-inflammatory responses and causes harm during respiratory viral infections.
Although the study is of interest, the mechanism proposed is largely circumstantial and needs to be further substantiated. There are also several of issues that need to be addressed as indicated below.

Specific comments

1. In the beginning of the Results section, the claim that ‘Ly6G-Cre mice provide to-date the most specific approach to study and track neutrophils is not right. First, Mrp8-Cre mice are commonly used and exhibit great specificity for neutrophils. Second, Ly6G+ macrophages in the lung have been recently described. Have the authors checked whether their effects are also coming from Ly6G+ macrophages?

2. The authors need to confirm that Nox2 deletion takes place in neutrophils and no other cells. They should use a more conventional approach such as cell sorting and PCR to show recombination.
The data, as they stand, are problematic. In Suppl. Fig. S1A, Nox2 expression appears to come from Ly6G- cells, i.e. cells other than neutrophils. Also, in Suppl. Fig. S1B, Nox2 deletion appears to take place both in Ly6G-CretdTomato +ve and Ly6G-CretdTomato -ve cells. How do the authors explain that? Is the deletion indeed specific to neutrophils?

3. Flow plots and gating strategies should be shown wherever needed (e.g. Fig.2, Fig. 3, S1 etc).

4. In the Results, it is claimed that ‘neutrophil-specific Nox2-deficient mice shift their lung cytokine profile towards IL-1β/IL-17A production while the expression of anti-viral type I IFNα and type III IFN declined quickly, inversely correlating with virus burden’. This is not exactly right as in Fig. 2 and Suppl. Fig. S2 type III IFN protein levels are not affected.

5. The presence of IL-1b, at least as %, seems to be much higher in monocytes, macrophages, conventional dendritic cells and alveolar macrophages compared to neutrophils. How do the authors know IL-1b is derived from neutrophils? The depletion of neutrophils through repeated injection of anti-Ly6G antibody in Nox2-/- mice of Fig. S4F is not convincing. First, it addresses IL-1b mRNA transcripts while it is known that IL-1b is largely preformed, and IL-1b regulation takes place at the protein level where post-translational processing is required. Second, it is done in global Nox2-/- mice. It needs to be better controlled to ensure neutrophil depletion does not affect the homing or production of other IL-1b producers etc.

6. As a follow up, flow plots for Fig. 3 and Fig. S4, especially for the various intracellular staining profiles, should be shown. Also, in Fig. S4, why have authors used Nox2-/- mice and not Ly6G-Cre+/- Nox2fl/fl mice? Why RNA levels are shown instead of IL-1b protein levels as in the case of the intracellular staining data?

7. In Fig. 3B and 3E, what does the “% frequency (right panel) of IL-1b+ neutrophils or IL-17+ gd T cells represent”? Frequency in which population?

8. What does the percentage of CellROX+ γδ T cells vs CD8 or CD4 T cells and NK cells indicate? Nox2 is expressed in neutrophils. Is the argument that neutrophil ROS can affect oxidative stress in another cell type? How would the authors envisage that to take place? Is there precedent for that?
Couldn’t increased CellROX positivity be due to the higher inflammatory response and/or viral presence affecting the state of γδ T cells?

9. Is cell-cell contact between neutrophils and gd T cells observed at the level of the tissue? Analyzing cells with ImageStream (Fig.3J) and assuming that doublets of neutrophils and gd T cells represent the in vivo situation is not accurate. Claims occur following the processing (digestion) of the tissue, the staining etc as a result of the tissue manipulation.

10. In Fig. 4A, an in vitro assay is shown when in vitro 'infection' of neutrophils takes place. Can neutrophils be infected with the virus? Does the virus replicate in them? Or neutrophils just take up the virus? In relation to that, what is accounting for the effects on neutrophils? Couldn't in be simply proteins/molecules in the virus preparation that trigger neutrophil activation?

11. Why p50 NFkB was studied? It would make more sense to study p65 as this is more relevant in terms of cytokine gene regulation, or assess more NFkB subunits.
Also, a 1.5-1.7 fold increase in p50 NFkB DNA binding activity (Fig. 4J) is really tiny compared to induction levels expected for NFkB to make significant differences in gene expression.

12. Why in the assay of Fig. 5A with human neutrophils polyI:C was used instead of influenza virus? Why this discordance with the mouse system?

13. How confident are the authors that the CD45RA- , CD11b+ , CD14- and CD16+ population are human neutrophils (Fig. S8A)? Couldn't these include eosinophils? Why additional markers were not used?

14. The experiments involving IL-17 or IL-1b neutralization are not comprehensive enough. Studies should include the main analyses and readouts of earlier studies such as weight loss, lung inflammation/damage etc.

15. How do the authors explain the fact that while IFNa is reduced, IFNb is increased? What impact could that have to their results

16. In the Fig 4J legend, it should be mentioned that this is p50 DNA binding activity.

17. The introduction is rather long and should be shortened and streamlined

18, There are no page numbers or line numbers, making it difficult to indicate what specific comments concern.

Reviewer #4

(Remarks to the Author)
The research article entitled "Neutrophil NADPH oxidase breaks the inflammatory IL-1β/IL-17A circuit to enhance pathogen clearance during respiratory virus infections" by Aderonke Sofoluwe, Angelos Petropoulos, Abhilesh Salil Goomanee, Zehra Fatima Ali-Khan and Annika Warnatsch, describes the results of a study aimed at investigating the role of the neutrophil NADPH oxidase NOX2 in Influenza A Virus (IAV) infection-induced inflammation. Using a Ly6G-Cre(+/-)/tdTomato Nox2 flox mouse, the results show that after IAV infection of these mice, ROS production was decreased, IL-1β and IL-17A production was increased, neutrophil-derived ROS affect γδ T cell proliferation and function, In the absence of neutrophil Nox2, increased neutrophil-derived IL-1β drives proliferation of IL-17 producing gamma delta (γδ) T cells (γδ17 T cells). This early enhancement of the IL-β/IL-17 axis counteracts the antiviral interferon response in IAV-infected mice. They then extended their findings to humans. Similar patterns of ROS production and cytokine regulation were observed in human neutrophils when exposed to the virus analogue poly(I:C) and SARS-CoV-2. The authors conclude that their data demonstrate that ROS, which are often associated with damage, play a dual role in regulating cytokine signalling and thus influencing the immune response to respiratory viruses. In general, the results are new and interesting, the presentation of the data is clear, but there are some points that need to be verified.

Specific comments:

1-All mouse results are based on the use of Ly6G-Cre(+/-)/tdTomato Nox2 flox mice, assuming that the Ly6G gene is specific for neutrophils. However, several recent studies have shown that macrophages, monocytes and myeloid-derived suppressor cells (MDSC) also express this protein (e.g. see Ruscitti C et al, Sci Immunol 2024,9,98,eado1227). The authors should purify all these cells from control and Ly6G-Cre(+/-)/tdTomato Nox2 flox mice and check the expression of NOX2/gp91phox by Western blot in uninfected and IAV-infected mice. Good quality commercial antibodies against NOX2/gp91phox are available.

2-Figure 1A: Looking more closely at the Figure 1A-NOX2-/- image, there is more DHE staining than in Ly6G-Cre(+/-) Nox2 flox mice? How can this be explained?

3-Figure 1E: Virus copy number: Looking closer at Figure 1E-day 3, there is more virus in Ly6G-Cre(+/-) Nox2 flox mice (about 1 million/microL) than in Nox2-/- and control mice (10 000/microL (100 times more))? How can this be explained? These data differ from those reported in the literature. Also, in all experiments, Nox2-/- should be compared with WT mice.

4-In Figure 1A,B,C,D: why was Ly6G-/- used as a control and not Ly6G+/- as in Figure 1F and Figure 2?

5-Why was the TNF concentration increased in Figure 2F but not the TNF mRNA in Figure 2?

6-figure 3K: The absence of Nox2 induced an increase in neutrophil T-cell interaction, how can this be explained?

7-figure 3J: In control cells, LT are more stained than neutrophils? This is not normal as neutrophils produce more ROS. In all Figures 3 uninfected versus AIV-infected mice should be shown.

8-Figure 4A and Figure 5C: To detect NADPH oxidase activation, the authors measured p47phox and p67phox colocalisation at the membrane. These cytosolic proteins are known to interact in the cytosol even in resting cells, colocalisation with Nox2 or p22phox is required to confirm activation.

9-Figure 4G: Same point as in "7": In control WT cells, LT are more stained than neutrophils? This is not normal as neutrophils produce more ROS. All Figure 4 should show resting versus activated cells. Same for Figure 5G.

10- The effect of hydrogen peroxide (H2O2) on IL-1 and IL-17 production by LT and neutrophils should be tested.

11-Minors:
a)-Introduction: a detailed paragraph on the NADPH oxidase subunits should be added to the introduction.

b)-Figure 2A-E: The x-axis should be labelled (time (days)??).

c)-Figure 4G: Why was IL-17 increased in WT neutrophils in Transwell?

Version 1:

Reviewer comments:

Reviewer #1

(Remarks to the Author)
The paper on Neutrophil NADPH oxidase breaks the inflammatory IL-1β-IL-17A circuit to enhance pathogen clearance during respiratory virus infections is of interest. The authors responded to my and to the questions of all referees. Neutrophil-gd T17 cell activation in IAV infected control is well documented by the use of Nox2 deficient KO mice is a great

Bernhard Ryffel

(Remarks on code availability)
No further comment;

Reviewer #2

(Remarks to the Author)
Dear,
I thank the authors for prividing this revised version of the manuscript and their efforts in answering all of my questions. These new data largely answered my concerns and in this new form, the paper is to my opinion suitable for publication.
Regards

(Remarks on code availability)

Reviewer #3

(Remarks to the Author)
The manuscript by Petropoulos et al is improved. However, some issues remain. Also, the lack of use of 'track changes' marking in the revised version makes the assessment of the revised version quite difficult.

Specific points
1. This point is not well addressed. The authors reply that 'Mrp8-Cre targets a broader population of myeloid cells, including monocytes, macrophages and dendritic cells' is not right. Where do they base that. It is well established that Mrp8-Cre mice are the most specific Cre tools for deleting something in neutrophils, and would have been more appropriate as they do not hit macrophages.
The fact that Ly6G-Cre target a subpopulation of macrophages, although small early on (but under expansion as it can reach up to 50% of all macrophages according to the original publication) does not mean they are not important in terms of the effects observed. Also, a population of 2-3% of all CD45+CD11b+ cells does not mean they are rare enough that they cannot be sorted and analyzed (for Nox2 deletion).
Anyway, at the minimum the authors should introduce a comment in the discussion indicating these limitations.

2. Unfortunately, again this argument is not easy to understand. It is not clear why in the initially submitted manuscript the fact that the gating strategy was based on Live $CD45^+$ $CD11b^+$ $Gr\text{-}1^+$ blood cells (initial Figure S1B) should give different results than when $CD45^+$ $CD11b^+$ cells were used instead. Why Nox2 expression was previously also coming present n neutrophils from Ly6GCre-/- mice but now that's not the case?
The new data of Suppl. Fig. S1C and D are good but they only come from Ly6G-Cre+/- Nox2fl/fl mice. They should include Ly6G-Cre-/- Nox2fl/fl mice as this is an important control especially in view of the previous submission indicating potential deletion of Nox2 in neutrophils from these mice as well.

3. This is insufficiently addressed as all gating strategies, and wherever needed in both Ly6G-Cre+/- Nox2fl/fl mice and Ly6G-Cre-/- Nox2fl/fl mice -in the various conditions- should be shown. Graphs can be short in size and fit but they are important as they convince the reader there are no other changes, (e.g. population shifts) affecting the results.
In 6E, even if intracellular cytokine staining plots for additional cell subsets, for macrophages the isotype control (for IL-1b and TNF) stained cells should be shown.

4. Although the authors have corrected the text, the main issue with respect to the mechanism proposed, i.e. the claim that 'this early self-amplified augmentation of the IL-1β/IL-17 axis counteracts the antiviral interferon response against IAV infection in mice.' remains This is also in the abstract. If IFN is not affected, IFNb is increased and only IFNa is reduced, how can the authors claim that?
A possibility is to claim that this is due to the IFNa decrease but will not be convincing enough for many. Have the authors examined the expression of ISFs, especially in the lung epithelium?
In any way, this needs to be corrected.

(Remarks on code availability)

Reviewer #4

(Remarks to the Author)
The revised version of the manuscript entitled « Neutrophil NADPH oxidase breaks the inflammatory IL-1β/IL-17A circuit to enhance pathogen clearance during respiratory virus infections », (NCOMMS-24-61358A), by Angelos Petropoulos et al., is well revised and improved, and addresses important points. I have no further comments.

(Remarks on code availability)

Version 3:

Reviewer comments:

Reviewer #3

(Remarks to the Author)

(Remarks on code availability)

Open Access This Peer Review File is licensed under a Creative Commons Attribution 4.0 International License, which permits use, sharing, adaptation, distribution and reproduction in any medium or format, as long as you give appropriate credit to the original author(s) and the source, provide a link to the Creative Commons license, and indicate if changes were

made.
In cases where reviewers are anonymous, credit should be given to 'Anonymous Referee' and the source.
The images or other third party material in this Peer Review File are included in the article's Creative Commons license, unless indicated otherwise in a credit line to the material. If material is not included in the article's Creative Commons license and your intended use is not permitted by statutory regulation or exceeds the permitted use, you will need to obtain permission directly from the copyright holder.
To view a copy of this license, visit https://creativecommons.org/licenses/by/4.0/

Reviewer's Comments:

Reviewer #1 (Remarks to the Author)

The paper on Neutrophil NADPH oxidase breaks the inflammatory IL-1β-IL-17A circuit to enhance pathogen clearance during respiratory virus infections is of interest and overall well presented.
The proposed model in Figure 6-F on neutrophil-gd T17 cell activation in IAV infected control and neutrophil deficient Nox2 KO mice is a great summary, which could serve as a graphical abstract.

We thank Reviewer #1 for their thoughtful and supportive comments. Below we provide detailed responses and indicate changes made in the revised manuscript.

A few minor issues should be considered:
1. Structure: The reading of the results is at some instances difficult due to regular switches to the supplementary data.

Response: We agree and have revised the manuscript to better integrate supplementary data with the main text. Transitions have been clarified, and supplementary figure references are now explained more thoroughly within the Results section.

2. Figure 1A: Y-axis: add label ROS DHE staining.

Response: The Y-axis label in Figure 1A has been updated to "ROS (DHE pixel intensity)".

3. Figure 1F: Add the quantitative analyses next to the immunofluorescent plates of NP and Ly6G;

Response: Quantification of NP and Ly6G staining area has been added next to the representative images in Figures 1G and H. The quantification method using ImageJ has been described in the Methods section page 10, line 162 - 167.

4. Figure 3: Revisit the outline of the figure allowing an easy read. Fig 3G: Please explain, I am lost.

Response: Figure 3 has been restructured for clarity. Panel 3I now includes a clearer legend and explanatory text in the Results section page 8, line 296 - 299. Additional details on the DNP derivatisation method for detecting protein carbonylation are included in the Methods section page 11, line 193 - 201.

5. Figure 5: Important data on human cells: would be great to add a scheme of the experimental approach.

Response: A schematic and description of the human neutrophil–γδ T cell co-culture experiment has been added to Figure 5 (Figure 5D).

Reviewer #2 (Remarks to the Author)

The manuscript by Sofoluwe and colleagues investigates the role of neutrophil-specific NADPH oxidase in regulating innate response during respiratory viral infection. Abrogation of Nox2 in neutrophils dampened viral clearance due to enhanced IL-1b-dependent proliferation of IL-17A-producing gdT cells. In turn, this counteracts antiviral IFN response. Such a pathway seems to be at play using human cells. These data are of great interest and provide important insights into our understanding of the immunopathology of respiratory viral infections. However, some additional data/controls are required to convey the general message. Here are my specific comments:

We thank Reviewer #2 for their constructive and insightful comments. Below we address each point in detail and outline the corresponding changes made to the revised manuscript.

1. Use of Ly6g-cre mice is indeed one of the most used model to specifically investigate neutrophil biology together with Mrp9-Cre. However, a recent study demonstrated that during IAV infection about 50% of CD11b+ Ly6G+ also expressed CD64 and were actually defined as macrophages (PMID: 39093958). This should be evaluated/controlled in this study even if macrophages are not huge producers of ROS.

Response: We thank the reviewer for highlighting this important point. We carefully evaluated the presence and potential contribution of $Ly6G^+$ $CD64^+$ macrophages during the early phase of IAV infection in our model. We note that the study by Ruscitti et al. observed $Ly6G^+$ monocyte-derived macrophages primarily during the recovery phase (day 10 post-infection), while our analyses focus on the early acute phase (days 1 – 5 post-infection).

To directly assess this, we performed flow cytometry at day 3 post-infection. Classical macrophages ($CD11b^+$ $Ly6G^-$ $CD64^+$) versus $Ly6G^+CD64^+$ macrophages are quantified in Supplementary Figure S4G – J. At 3 dpi, $Ly6G^+CD64^+$ macrophages were a minor population, with absolute counts of approximately $1.25 \pm 0.7 \times 10^5$ per 100mg lung tissue in Ly6G-Cre and $1.25 \pm 0.6 \times 10^5$ in Ly6G-Cre Nox2 flox mice – substantially lower than classical macrophages (2.5 ± 0.7 and $3.9 \pm 1 \times 10^6$ respectively). Their frequency within Live $CD45^+$ $CD11b^+$ cells was also low (2.6 ± 2 % and 2.0 ± 0.8 %, respectively), compared to classical macrophages (43.1 ± 8.3 % and 53.3 ± 7.8 %). Importantly, no significant difference in the frequency or number of $Ly6G^+CD64^+$ macrophages was observed between the two genotypes, indicating that their expansion is not affected by Nox2 deficiency.

Finally, we confirmed that Nox2 deletion was restricted to neutrophils (gated as Live $CD45^+$ $CD11b^+$ $Ly6G^+$ $CD64^-$) using qPCR of sorted populations (Figure S1C and D). Due to the low abundance of $Ly6G^+CD64^+$ macrophages, we were unable to sort sufficient

cell numbers for reliable qPCR analysis in this population. However, Nox2 expression was preserved in classical macrophages and Ly6C^{hi} monocytes.

Together, these data demonstrate that Ly6G^{+}CD64^{+} macrophages are present in very low numbers during early IAV infection and are not significantly affected by Ly6G-Cre–mediated Nox2 deletion. We therefore conclude that the phenotypes observed in our model are driven by Nox2-deficient neutrophils rather than Ly6G^{+} macrophages. We added this to the results section page 4 line 120-130.

2. It is now well established that neutrophil are functionally heterogeneous especially upon respiratory infections. A further phenotypical characterization of neutrophils would be of interest regarding Nox2 expression and IL-1b secretion.

Response: We performed additional phenotypical characterisation of lung neutrophils at day 3 post-IAV infection in Ly6G-Cre and Ly6G-Cre Nox2 flox mice, as shown in Supplemental Figure S7A–G. Specifically, we analysed the surface expression of a panel of activation and differentiation markers, including CD10, CD44, CD101, CD63, CXCR2, PDL1, Sca1, CD11a, Siglec-F, CD62L and CD49d. While infection broadly induced upregulation of activation markers across genotypes, most markers showed no significant differences between Ly6G-Cre and Ly6G-Cre Nox2 flox mice. However, our analysis revealed several distinct phenotypic alterations in Nox2-deficient neutrophils, consistent with altered functional states. CD63 and Siglec-F were significantly upregulated in Ly6G-Cre Nox2 flox neutrophils, both in terms of frequency and mean fluorescence intensity (MFI). This suggests increased degranulation activity (CD63) and a skewing toward an aged or tissue-adapted phenotype (Siglec-F). Conversely, we observed reduced MFI of CD11a and CD44 on Nox2-deficient neutrophils indicating changes in adhesion and migration properties, as well as reduced MHC class II. Diminished MHC II expression (though overall low) suggests these neutrophils do not acquire antigen-presenting features. Notably, CXCR2 and CD11b expression did not differ between genotypes, indicating that chemokine responsiveness and overall activation status remain comparable between Nox2-deficient and control neutrophils.

These results support the concept that Nox2 shapes neutrophil phenotype beyond ROS production, contributing to distinct cellular programming during respiratory viral infection. The observed differences in surface marker expression align with the enhanced IL-1β production and inflammatory potential of Nox2-deficient neutrophils documented in Figure 3A and B. We added this to the results section page 6 - 7 line 236-247 and discussion page 13 line 489 - 492.

3. The increased frequency of CD3+ T cells in Ly6G-cre+/- Nox2 flox that seems to be independent of CD4+ and CD8+ T cells cannot be solely explained by the increased in gdT cells from a numerical perspective. It is known that DN abT cells can expand upon IAV infection (PMID: 39093958). The influence of neutrophilic Nox2 on this population should be explored (function, phenotype,...).

Response: We thank the reviewer for this important observation. To investigate the contribution and characteristics of DN αβ T cells in Ly6G-Cre Nox2 flox mice, we performed extended flow cytometric analyses at days 3 and 7 post-IAV infection (Supplemental Figures S8A–K and S6L). At 3 days post-infection, we observed a reduction in $CD4^+$ and $CD8^+$ αβ T cell frequencies (figure S3D and E), accompanied by a significant increase in the frequency and absolute number of DN αβ T cells (gated as Live $CD45^+$ $CD3^+$ $TCR\gamma\delta^-$ $CD4^-CD8^-$) in Ly6G-Cre Nox2 flox mice (Figure S8A). By day 7, DN T cell frequency and numbers returned to levels comparable to control mice, indicating a transient accumulation during early infection.

We further assessed functional potential by measuring intracellular IL-17A and IFNγ, but did not detect cytokine production in DN αβ T cells at 3 dpi in either genotype (Figure S6L). Moreover, DN T cells were not affected by neutrophil Nox2-induced oxidative stress, with a similar low percentage of DN T cells staining $CellROX^+$ in Ly6G-Cre and Ly6G-Cre Nox2 flox mice (Figure S9D left panel).

To better understand their activation state, we analysed multiple surface markers associated with T cell activation, trafficking, and metabolism (Figure S8B-K). Compared to control mice, DN αβ T cells in Ly6G-Cre Nox2 flox mice exhibited reduced $CD62L^-$ frequency, suggesting impaired effector transition or delayed activation. Mean fluorescence intensity of CD25, CD71 and CD98 indicate diminished activation and metabolic readiness. Moreover, downregulation of CCR2 and CXCR3, implying altered migratory potential or tissue recruitment. Further, increased frequency of $CD27^+$ DN T cells, consistent with a less differentiated, possibly central-memory–like phenotype. We have added this to the results section on page 7 - 8 line 271 - 286.

Taken together, these findings suggest that Nox2-deficient neutrophils alter the early T cell landscape, promoting transient expansion of DN αβ T cells with an attenuated activation phenotype. The simultaneous increase in DN αβ T cells and reduction in $CD4^+$/$CD8^+$ subsets, raises the possibility that T cell subset differentiation may be stalled, potentially due to altered inflammatory cues or antigen-presenting signals in the absence of neutrophilic Nox2. While our data cannot confirm whether DN αβ T cells arise from a failure to commit to the CD4 or CD8 lineage, this altered balance and phenotype underscore a broader immunomodulatory role of neutrophil-derived ROS in shaping T cell responses during IAV infection. We have added this to the discussion on pages 14 - 15 lines 564 - 572.

While our data do not directly demonstrate whether these DN αβ T cells arise due to failed lineage commitment to the $CD4^+$ or $CD8^+$ fate, the accumulation of a functionally inert and incompletely activated DN T cell population in the absence of neutrophilic Nox2 suggests that Nox2-expressing neutrophils influence timely T cell subset differentiation and effector programming during early IAV infection.

4. The use of splenic and LN gdT cells may not be suitable for the co-culture experiment since most of the purified cells from these organs are CD27+ (IFNg+) (PMID: 39093958). It would be of interest to use lung gdT cells that are mainly IL-17-producing.

Response: We thank the reviewer for this important point. It is correct that γδ T cells isolated from secondary lymphoid organs are predominantly $CD27^+$ IFNγ-producing, whereas lung-resident γδ T cells tend to be $CD27^-$ IL-17–producing, as supported by Ribot et al. 2009 (https://doi.org /10.1038/ni.1717). We used splenic γδ T cells due to higher purity upon isolation (75.5 ± 3.6% γδ T cells of live CD45 (mean ± SD of 3 independent experiments), figure S10B).

To directly address the reviewer's concern, we repeated the co-culture experiment using lung-isolated γδ T cells, which are inherently enriched in $CD27^-$ IL-17–producing subsets (Supplemental figure S12). As shown in figure S12A, although the isolation was less pure (50.3 ± 4.6% γδ T cells of live CD45 (mean ± SD of 3 independent experiments), figure S12A), we nevertheless observed a significant induction of IL-17A production upon co-culture with neutrophils (S12E and F). Importantly, this induction was markedly enhanced in the presence of Nox2-deficient neutrophils, confirming the generalisability and physiological relevance of our findings to the lung immune environment. We further observed neutrophils and lung-isolated γδ T cells in coculture by immunofluorescence and found significantly more $CellROX^+$ γδ T cells in contact with WT neutrophils compared to Nox2-deficient neutrophils upon IAV infection (S12C and D)

Together, these data demonstrate that neutrophilic Nox2 constrains γδ T cell IL-17 production both in splenic and lung-derived populations. We added these results on page 8 – 9, lines 321 – 328.

5. It is unclear to me how Imagestream can be used to evaluate neutrophil-gdT cell interaction since it is a flow-based strategy.

Response: We appreciate the reviewer's concern and agree that Imagestream is not suited to quantify dynamic or spatially contextualised cell–cell interactions in tissues. In our initial analysis, we observed a population of doublets positive for γδTCR and Ly6G in flow cytometry, which prompted us to further examine these events using Imagestream. However, we acknowledge that this approach cannot assess the true frequency or physiological relevance of neutrophil–γδ T cell interactions in vivo within the lung microenvironment.

In response to this concern, we have removed the Imagestream data and instead focused on immunofluorescence-based imaging of lung tissue, which provides spatial resolution and localisation. As shown in Figure 3K, we used Ly6GtdTomato as a neutrophil marker and γδTCR staining to demonstrate close spatial proximity between neutrophils and γδ T cells at sites of infection within the lung.

In addition, we observed proximity between neutrophils and γδ T cells in ex vivo co-culture systems. These interactions are visualised in Figure 4D between spleen-isolated γδ T cells and neutrophils and Supplemental Figure S12C between lung-isolated γδ T cells and neutrophils, confirming that such interactions occur in a controlled setting and supporting the functional data presented.

Taken together, we provide tissue-based and ex vivo evidence of neutrophil–γδ T cell interaction, which more appropriately addresses the reviewer's concerns and strengthens the mechanistic relevance of our findings.

6. The association between IL-17 and viral loads is interesting. How is the viral load in Tcrd-/- mice reconstituted with either WT or Il17-/- gdT cells?

Response: We appreciate the reviewer's interest in the mechanistic link between γδ T cell–derived IL-17 and viral control. We respectfully consider that the suggested reconstitution experiment in $Tcrd^{-/-}$ mice using WT or $IL17^{-/-}$ γδ T cells goes beyond the scope and focus of the current manuscript, which is centred on neutrophil-derived ROS and its regulatory role on γδ T cell activation during early IAV infection. While we do observe an association between elevated IL-17A and higher viral loads, our primary aim was to dissect how neutrophilic Nox2 influences the immunomodulatory axis between neutrophils and γδ T cells, rather than to explore the functional role of IL-17A in viral clearance in isolation.

Importantly, several studies have highlighted the context-dependent role of IL-17A and γδ T cells during IAV infection. Early IL-17 signalling in the upper airways can support pathogen clearance and promote epithelial repair, as shown in models of mild IAV infection (Guo et al. 2018 https://doi.org/10.1016/j.immuni.2018.07.011 and Palomina-Segura et al. 2020 https://doi.org/10.1002/eji.201948157). In contrast, excessive or prolonged IL-17 responses have been associated with compromised barrier integrity and exacerbated inflammation, particularly in models of severe H1N1 infection and combined exposure to cigarette smoke and IAV (Xue et al. 2017 https://doi.org/10.3389/fimmu.2017.01054 and Hong et al. 2018 https://doi.org/10.1038/mi.2017.93).

We agree that the precise contribution of IL-17A from γδ T cells to viral control is an important and complex topic, and we believe it would be better addressed in a dedicated follow-up study, where adoptive transfer systems and broader cytokine analyses can be systematically investigated. We have added further discussion of this topic on page 14 lines 542 – 546.

Minor points:
7. The gating strategy for lung immune cells should be clearly stated. For instance, it is unclear why the number of alveolar macrophages increases during IAV infection since AM depletion is a hallmark of this infection. To compare the absolute counts with other studies, it would be of interest to show the data as total number per lung.

Response: We have now clearly outlined our gating strategy for lung immune populations in the Supplemental Figures S3F (T cells and NK cells), S3G (neutrophils, monocytes, and macrophages), and S4K (alveolar macrophages and cDCs).

To address the reviewer's concern regarding alveolar macrophage (AM) dynamics during IAV infection, we note that while AM depletion is a hallmark in many infection models, the extent and kinetics of this depletion can vary with strain, dose, and

experimental conditions. In our study, we observed a transient increase in AM numbers at day 3 post-infection, from approximately 8.8 ± 1.5 × 10^4 cells per 100 mg lung tissue in naïve lungs to 6.2 ± 2.8 × 10^5 (mean ± SD of 20 animals), followed by decline at day 7. These data are now shown in Supplemental Figure S4A and B. Our findings are consistent with previous reports. For example, Li et al. (2022) observed similar AM dynamics in their study of monocyte-derived alveolar macrophages during IAV infection (https://doi.org/10.1126/sciimmunol.abj5761). The temporary increase may reflect inflammatory recruitment and local proliferation, as also reported in mild infection models.

Importantly, we did not observe significant differences in alveolar macrophage numbers between Ly6G-Cre and Ly6G-Cre Nox2 flox mice, suggesting that Nox2 deficiency in neutrophils does not substantially impact this compartment.
To improve comparability with the literature, we have now provided the data as total cell numbers per 100 mg lung tissue (Figure S4A).

8. “adaptive type 2 IFN-y”: this sentence should be rephrased as it is uncertain whether this IFN-y production is dependent on innate or adaptive cells.

Response: We have changed the results description accordingly on page 6 line 211.

9. “In addition, an increased % of gdT cells … RORgt in co-culture with Nox2-/- neutrophils”. This sentence should be rephrased because it suggest a shift in the expression of this peculiar TF. However, the data presented in Fig.S7b solely suggest that IL-17A producing cells are RORgt. What is the frequency of total RORgt-expressing cells upon coculture.

Response: We changed the results description accordingly on page 9 lines 338 - 341. The data does not imply that RORγt expression is upregulated by Nox2 deficiency, instead we observe an increased frequency and functional skewing within the γδ T cell population. We observe a significant increase in the frequency of $ROR\gamma t^+$ γδ T cells in coculture with IAV-infected Nox2-deficient neutrophils compared to WT neutrophils among total live $CD45^+$ cells (figure S11D), and of $IL17A^+$ $ROR\gamma t^+$ double-positive cells within the γδ T cell compartment (figure S11C).

Reviewer #3 (Remarks to the Author)

Sofoluwe et al. have investigated the role of neutrophil-derived ROS in modulating the immune response during respiratory viral infection. They used as a model influenza A virus infection in mice and found that neutrophil-specific Nox2-deficient mice exhibit heightened lung inflammation and impaired virus clearance. They further found that neutrophil Nox2-derived ROS are involved in this process by reducing IL-1b production and curbing the proliferation of IL-17-producing gamma delta (γδ) T cells. They therefore concluded that neutrophil Nox2 and ROS act as inhibitors of an IL-1b/IL-17 axis that augments pro-inflammatory responses and causes harm during respiratory viral infections.
Although the study is of interest, the mechanism proposed is largely circumstantial and

needs to be further substantiated. There are also several of issues that need to be addressed as indicated below.

We are grateful to Reviewer #3 for their helpful and thought-provoking feedback. Please find our point-by-point responses below, along with a summary of revisions incorporated into the manuscript.

Specific comments

1. In the beginning of the Results section, the claim that 'Ly6G-Cre mice provide to-date the most specific approach to study and track neutrophils is not right. First, Mrp8-Cre mice are commonly used and exhibit great specificity for neutrophils. Second, Ly6G+ macrophages in the lung have been recently described. Have the authors checked whether their effects are also coming from Ly6G+ macrophages?

Response: We appreciate the reviewer's point and have clarified our model in Figure S1 accordingly. While both Mrp8-Cre and Ly6G-Cre mice are widely used to study neutrophils, Mrp8-Cre targets a broader population of myeloid cells, including monocytes, macrophages and dendritic cells. In contrast, Ly6G-Cre mice offer higher specificity for neutrophils, as shown by Hasenberg et al., 2015 (https://doi.org/10.1038/nmeth.3322), and thus are particularly well suited for studying neutrophil-specific gene deletion in vivo. We have revised our text to acknowledge the use of both models and highlight the respective advantages of Ly6G-Cre for our specific application (see revised page 3, lines 109 - 113).

In response to the reviewer's second point, we carefully evaluated the presence and potential contribution of $Ly6G^+$ $CD64^+$ macrophages in our model during early IAV infection (days 1 – 5 post-infection). Notably, a recent study by Ruscitti et al., 2023 (https://doi.org/10.1126/sciimmunol.ado1227) described $Ly6G^+$ monocyte-derived macrophages predominantly during the recovery phase (day 10 post-infection). Since our study focuses on the acute phase, we specifically assessed this population at day 3 post-infection by flow cytometry.

As shown in Supplementary Figures S4G – J, $Ly6G^+$ $CD64^+$ macrophages were present but represented a minor subset, with absolute counts of approximately $1.25 \pm 0.7 \times 10^5$ and $1.25 \pm 0.6 \times 10^5$ cells per 100 mg lung tissue in Ly6G-Cre and Ly6G-Cre Nox2 flox mice, respectively. In contrast, classical macrophages ($Ly6G^-$ $CD64^+$) were significantly more abundant (2.5 ± 0.7 and $3.9 \pm 1.0 \times 10^6$, respectively). Their frequency among Live $CD45^+$ $CD11b^+$ cells was also low (2.6 ± 2 % and 2.0 ± 0.8 %) compared to classical macrophages (43.1 ± 8.3 % and 53.3 ± 7.8 %). Importantly, there were no significant differences in the frequency or absolute number of $Ly6G^+$ $CD64^+$ macrophages between genotypes, indicating that their expansion is not influenced by Nox2 deficiency.

To assess the specificity of Nox2 deletion, we performed qPCR on sorted lung populations. As shown in Figure S1C and D, Nox2 expression was efficiently ablated in neutrophils ($CD45^+$ $CD11b^+$ $Ly6G^+$ $CD64^-$), while preserved in classical macrophages

($CD64^+$ $Ly6G^-$) and $Ly6C^{hi}$ monocytes. Due to their low abundance, $Ly6G^+CD64^+$ macrophages could not be reliably sorted for qPCR.
We have added these results to Figure S1 and results section pages 3 - 4, lines 116 - 130 in the revised manuscript.

2. The authors need to confirm that Nox2 deletion takes place in neutrophils and no other cells. They should use a more conventional approach such as cell sorting and PCR to show recombination.
The data, as they stand, are problematic. In Suppl. Fig. S1A, Nox2 expression appears to come from Ly6G- cells, i.e. cells other than neutrophils. Also, in Suppl. Fig. S1B, Nox2 deletion appears to take place both in Ly6G-CretdTomato +ve and Ly6G-CretdTomato -ve cells. How do the authors explain that? Is the deletion indeed specific to neutrophils?

Response: We thank the reviewer for this important observation. In our initial intracellular flow cytometry analysis (Figure S1B), the gating strategy was based on Live $CD45^+$ $CD11b^+$ $Gr\text{-}1^+$ blood cells. However, intracellular Nox2 staining exhibited variability and showed a high proportion of Nox2-negative cells even in control animals, highlighting the limitations of this approach for clearly identifying specific deletion.

To address this, we have now refined the gating strategy to focus on Live $CD45^+$ $CD11b^+$ myeloid cells. Within this population, we analysed tdTomato and Nox2-FITC fluorescence in blood from Ly6G-$Cre^{-/-}$ Nox2 flox, Ly6G-$Cre^{+/-}$, and Ly6G-$Cre^{+/-}$ Nox2 flox littermates. This updated analysis demonstrates that $tdTomato^+$ cells in Ly6G-$Cre^{+/-}$ Nox2 flox mice lack Nox2 expression, whereas Nox2 expression is maintained in Ly6G-$Cre^{+/-}$ control mice. Importantly, the percentage of $tdTomato^-$ myeloid cells expressing Nox2 is comparable between Ly6G-$Cre^{+/-}$ and Ly6G-$Cre^{+/-}$ Nox2 flox mice, indicating that Nox2 deletion is confined to the neutrophil ($tdTomato^+$) population and does not occur in other myeloid cells.

To further support this conclusion, we have now included qPCR data from FACS-sorted neutrophils (Live $CD45^+$ $CD11b^+$ $Ly6G^+$ $CD64^-$), confirming robust and specific deletion of Nox2 transcripts in neutrophils of Ly6G-$Cre^{+/-}$ Nox2 flox mice (Figure S1C and D).

3. Flow plots and gating strategies should be shown wherever needed (e.g. Fig.2, Fig. 3, S1 etc).

Response: Gating strategies are described in the respective figure legends, we agree that visual presentation enhances clarity and reproducibility. We have therefore included representative flow cytometry plots to illustrate key gating steps. For figure 2, gating strategies for neutrophils, monocytes, macrophages, NK and T cells are now shown in supplemental figures S3A and B. For figure 3, the gating strategy for γδ T cells is also included in S3A, and representative FACS plots for Ki-67, CellROX and Annexin V staining are provided in supplemental figure S9. Altogether, we have clarified the gating information and added relevant plots where appropriate (also see supplemental figures S4K and S6E).

4. In the Results, it is claimed that 'neutrophil-specific Nox2-deficient mice shift their

lung cytokine profile towards IL-1β/IL-17A production while the expression of anti-viral type I IFNα and type III IFNλ declined quickly, inversely correlating with virus burden'. This is not exactly right as in Fig. 2 and Suppl. Fig. S2 type III IFNλ protein levels are not affected.

Response: We agree that the protein levels of IFNλ (measured by ELISA in Figure 2M) were not significantly different between genotypes. However, we did observe a small but statistically significant reduction in Ifnλ mRNA expression at 3 days post-infection (Figure 2O). Additionally, the Proteome Profiler array in Supplemental Figure S5B shows a subtle decrease in IFNλ signals in Ly6G-Cre+/- Nox2 flox mice compared to controls. We have amended the manuscript text accordingly on page 6 lines 207 – 211 to emphasize the decline in type I IFNα levels as the more consistent observation, and to clarify that IFNλ expression was only modestly reduced.

5. The presence of IL-1b, at least as %, seems to be much higher in monocytes, macrophages, conventional dendritic cells and alveolar macrophages compared to neutrophils. How do the authors know IL-1b is derived from neutrophils? The depletion of neutrophils through repeated injection of anti-Ly6G antibody in Nox2-/- mice of Fig. S4F is not convincing. First, it addresses IL-1b mRNA transcripts while it is known that IL-1b is largely preformed, and IL-1b regulation takes place at the protein level where post-translational processing is required. Second, it is done in global Nox2-/- mice. It needs to be better controlled to ensure neutrophil depletion does not affect the homing or production of other IL-1b producers etc.

Response: We do not conclude that all of the increased IL-1β measured in the BAL of Ly6G-Cre Nox2 flox mice originates exclusively from neutrophils. Rather, our data support the conclusion that the absence of Nox2 in neutrophils alone is sufficient to initiate increased IL-1β production. This likely triggers an inflammatory feedback cascade, in which elevated IL-1β from neutrophils promotes γδ T cell activation and IL-17 release, followed by enhanced recruitment and activation of monocytes and macrophages, which subsequently contribute to the IL-1β pool. We have clarified this in the results (page 6 lines 222 – 236) and discussion section (page 13 line 487 - 489).

Furthermore, we do observe transcriptional upregulation of IL-1β in whole lungs of Ly6G-Cre Nox2 flox mice (Figure 2N) and in isolated neutrophils from Nox2-/- mice (Figure 4J), in which NF-κB activity is increased (Figure 4K), suggesting that neutrophils contribute to IL-1β production not only through processing but via enhanced transcription.

To strengthen our conclusions, we now include data demonstrating that neutrophil depletion in Ly6G-Cre Nox2 flox mice (Figure S6F–H) reduces both IL-1β mRNA expression in lung tissue and IL-1β protein levels in BAL. This supports a model in which neutrophil-derived signals, specifically from Nox2-deficient neutrophils, initiate an inflammatory cascade that amplifies IL-1β production through downstream effects on other immune cell populations.

6. As a follow up, flow plots for Fig. 3 and Fig. S4, especially for the various intracellular staining profiles, should be shown. Also, in Fig. S4, why have authors used Nox2-/- mice and not Ly6G-Cre+/- Nox2fl/fl mice? Why RNA levels are shown instead of IL-1b protein levels as in the case of the intracellular staining data?

Response: As noted above, we do not conclude that neutrophils are the sole source of IL-1β in Ly6G-Cre Nox2 flox mice, but rather that loss of Nox2 in neutrophils triggers a cascade that amplifies IL-1β production involving other cell types. Regarding the use of Nox2-/- mice in the neutrophil depletion experiment shown in Figure S6, we agree that Ly6G-Cre Nox2 flox mice provide a more specific model. We have therefore performed additional experiments using neutrophil-specific Nox2-deficient mice (Figure S6F–H) and now show that neutrophil depletion in this model reduces both IL-1β mRNA and protein levels in BAL, thereby confirming that Nox2-deficient neutrophils contribute to increased IL-1β expression in vivo.

In response to the reviewer's request, we now include additional representative flow cytometry plots where space allows. Specifically, the gating strategy for γδ T cells is shown in Supplemental Figure S3A, and representative intracellular cytokine staining for neutrophils, monocytes and γδ T cells is shown in Figures 3A, D and G. Further representative plots for Ki67, CellROX, and Annexin V staining are included in Figure S9.

We now also include representative flow plots for IL-1β and TNFα staining in alveolar macrophages in Figure S6E. Due to space constraints, we do not display all intracellular cytokine staining plots for additional cell subsets (e.g. dendritic cells, macrophages, $CD4^{+}$, $CD8^{+}$, and DN T cells) but can provide these upon request.

7. In Fig. 3B and 3E, what does the "% frequency (right panel) of IL- 1b+ neutrophils or IL-17+ gd T cells represent"? Frequency in which population?

Response: In Figure 3B, the frequency refers to the percentage of IL-1β+ cells within the neutrophil population (gated as CD45+ CD11b+ Ly6G+). In Figure 3F (former E), the frequency refers to IL-17+ cells within the γδ T cell population (gated as CD45+ CD3+ γδTCR+). We have clarified this in the revised figure legend accordingly.

8. What does the percentage of CellROX+ γδ T cells vs CD8 or CD4 T cells and NK cells indicate? Nox2 is expressed in neutrophils. Is the argument that neutrophil ROS can affect oxidative stress in another cell type? How would the authors envisage that to take place? Is there precedent for that?
Couldn't increased CellROX positivity be due to the higher inflammatory response and/or viral presence affecting the state of γδ T cells?

Response: We thank the reviewer for this important question. Indeed, our study shows that Nox2-derived ROS from neutrophils can induce oxidative stress in other immune cell types, in our model specifically γδ T cells. There is established precedent for this form of intercellular ROS transfer. For example, Mensurado et al. 2018

(https://doi.org/10.1371/journal.pbio.2004990) showed that tumor-associated neutrophils via H_2O_2 transfer suppress IL-17 producing γδ T cells.
Pillay et al., JCI 2012 (https://doi.org/10.1172/JCI57990) demonstrated local release of H_2O_2 from neutrophils into the immunological synapse to suppress T cell proliferation, mediated by Mac-1–dependent contact. Petzold et al., Immunity 2022 (https://doi.org/10.1016/j.immuni.2022.10.001) showed that neutrophils regulate megakaryocyte function via contact-dependent ROS transfer. We have added discussion of this literature in the discussion section page 13 lines 515 - 521.

In our study, we observe this phenomenon both in vivo and ex vivo. In the in vivo setting, we find elevated oxidative stress (CellROX positivity) in γδ T cells from Ly6G-Cre+/- mice compared to Ly6G-Cre+/- Nox2 flox mice (Figure 3J). Interestingly, Ly6G-Cre+/- Nox2 flox mice display higher viral burden (Figure 1E), increased proinflammatory cytokine levels (Figure 2L and N), more severe lung inflammation (see histology Figure S2C-E), but reduced oxidative stress in the immune cell compartment (Figure 1C), demonstrating the suppressive effect of neutrophil-derived ROS.

This interpretation is further supported by ex vivo co-culture experiments, where γδ T cells cultured with wild-type neutrophils show strong CellROX positivity indicative of oxidative stress. This effect is abolished when neutrophils are replaced with Nox2-deficient neutrophils or when the two cell types are separated by a Transwell insert, preventing direct contact. These findings reinforce that ROS transfer is proximity-dependent and neutrophil-Nox2 dependent, and not merely a result of general inflammation or viral exposure.

9. Is cell-cell contact between neutrophils and gd T cells observed at the level of the tissue? Analyzing cells with ImageStream (Fig.3J) and assuming that doublets of neutrophils and gd T cells represent the in vivo situation is not accurate. Claims occur following the processing (digestion) of the tissue, the staining etc as a result of the tissue manipulation.

Response: We agree with the reviewer that ImageStream analysis is not suitable for accurately assessing physiological cell-cell interactions within tissue due to potential artefacts introduced during sample processing, including enzymatic digestion and mechanical dissociation. In response to this concern, we have removed the ImageStream data (previously shown in Figure 3J) from the revised manuscript.

Instead, we now focus on immunofluorescence staining of lung tissue, which directly demonstrates spatial proximity between neutrophils and γδ T cells in situ. As shown in Figure 3K, neutrophils (marked by Ly6G) and γδ T cells (stained for γδ TCR) are found in close proximity particularly near sites of IAV infection. These findings support the presence of in vivo interactions between the two cell types under physiological conditions.

Additionally, ex vivo co-culture experiments (Figures 4D and S12E) confirm direct contact between neutrophils and γδ T cells, reinforcing our conclusion that such interactions are biologically relevant and contribute to the observed effects on oxidative

stress and cytokine production.

10. In Fig. 4A, an in vitro assay is shown when in vitro 'infection' of neutrophils takes place. Can neutrophils be infected with the virus? Does the virus replicate in them? Or neutrophils just take up the virus? In relation to that, what is accounting for the effects on neutrophils? Couldn't in be simply proteins/molecules in the virus preparation that trigger neutrophil activation?

Response: We thank the reviewer for raising this important point. Neutrophils are not susceptible to productive infection by influenza A virus (IAV), and the virus does not replicate in these cells. Instead, neutrophils recognize viral pathogen-associated molecular patterns (PAMPs) via surface and endosomal receptors (e.g. TLR3, TLR7, TLR8, RIG-I), which are sufficient to activate downstream signaling pathways, including the assembly and activation of the Nox2. We have revised the manuscript to describe these experiments as "in vitro IAV stimulation" instead of "infection" and clarified this on page 8 lines 313 – 315.

We also confirm that highly purified virus preparations were used to minimize confounding effects of contaminating host proteins or cytokines (see Method section page 7 lines 57 – 63). This stimulation with purified IAV induces Nox2 activation through innate immune recognition, consistent with established literature (e.g. Malachowa et al., 2018 https://doi.org/10.1128/mSphereDirect.00567-17;Wang et al., Blood 2008, https://doi.org/10.1182/blood-2008-01-132860).

11. Why p50 NFkB was studied? It would make more sense to study p65 as this is more relevant in terms of cytokine gene regulation, or assess more NFkB subunits.
Also, a 1.5-1.7 fold increase in p50 NFkB DNA binding activity (Fig. 4J) is really tiny compared to induction levels expected for NFkB to make significant differences in gene expression.

Response: We initially focused on the p50 subunit because previous studies have highlighted its redox sensitivity, oxidation of p50 disrupts its DNA-binding ability, thus impairing NF-κB-mediated transcription (e.g. Warnatsch et al., Immunity 2017, https://doi.org/doi: 10.1016/j.immuni.2017.02.013). Given the central role of ROS in our study, we considered p50 an appropriate starting point.

However, to more directly assess transcriptionally active NF-κB complexes, we now performed DNA-binding activity assays for the p65 subunit. As shown in the revised Figures 4K and 5K, we observe a 4-fold increase in p65 DNA-binding activity in human neutrophils pre-treated with the Nox2 inhibitor Apocynin upon IAV infection**,** and a 2.5-fold increase in Nox2 -/- neutrophils compared to wildtype controls, consistent with the activity of the positive control (activated Jurkat nuclear extract). These findings better reflect the transcriptional activation potential of NF-κB in the absence of ROS and confirm that Nox2 deficiency enhances p65-mediated inflammatory gene expression.

12. Why in the assay of Fig. 5A with human neutrophils polyI:C was used instead of influenza virus? Why this discordance with the mouse system?

Response: In response, we now include additional experiments using IAV with human neutrophils in Figures 5C and K, S13F, providing direct comparison with our poly(I:C) results.

We initially used poly(I:C) as a synthetic viral double-stranded RNA analogue to model viral recognition. This approach is widely used to mimic viral infections and trigger antiviral pathways, including type I IFN production and ROS induction via pattern recognition receptors (e.g., TLR3, RIG-I and MDA5). We believe that including both poly(I:C) and IAV-based experiments underscores the generalisability of our findings and demonstrates the robustness of the observed neutrophil-mediated responses.

Moreover, human neutrophils are not productively infected by IAV, but they do respond to viral PAMPs through surface and endosomal receptors. This recognition is sufficient to activate NADPH oxidase (NOX2) and induce ROS production, as previously reported (e.g. Malachowa et al., 2018 https://doi.org/10.1128/mSphereDirect.00567-17;Wang et al., Blood 2008, https://doi.org/10.1182/blood-2008-01-132860). Therefore, the use of poly(I:C) does not represent a discordance but rather complements and extends the mouse model findings.

13. How confident are the authors that the CD45RA- , CD11b+ , CD14- and CD16+ population are human neutrophils (Fig. S8A)? Couldn't these include eosinophils? Why additional markers were not used?

Response: The human neutrophil isolation method we employed is based on centrifugation over a discontinuous Percoll gradient, as described by Aga et al., 2002 (https://doi.org/10.4049/jimmunol.169.2.898). This protocol is well established and widely used to obtain highly purified neutrophil populations from human peripheral blood. To further confirm the identity of our neutrophils and exclude eosinophil contamination, we have now included additional surface markers in our analysis. As shown in the revised Figure S13A, neutrophils were gated as Live CD45RA$^-$, CD11b$^+$, CD14$^-$, CD16$^+$, CD66b$^+$, CCR3$^-$, and Siglec-8$^-$. On average, 99.1 ± 0.4 % of Live CD45$^+$ gated cells were neutrophils, confirming minimal contamination by eosinophils. This comprehensive panel strengthens our confidence in the purity of our neutrophil preparations (page 10, lines 393 – 396).

14. The experiments involving IL-17 or IL-1b neutralization are not comprehensive enough. Studies should include the main analyses and readouts of earlier studies such as weight loss, lung inflammation/damage etc.

Response: We have expanded our analysis to include further readouts following IAV infection in mice treated with IL-17 and IL-1β neutralising antibodies.
In Figure 6F, body weight was monitored daily for up to 5 days post-infection. Mice treated with anti-IL-17 and anti-IL-1β antibodies exhibited significantly less weight loss compared to isotype control-treated mice, with marked improvement observed between days 3 and 5 post-infection, indicating a protective effect of cytokine blockade. Moreover, Figure 6G shows that neutralisation of IL-17 and IL-1β significantly reduced lung inflammation and tissue damage, as assessed by histopathological

scoring of lung sections. Treated animals exhibited lower levels of cellular infiltration, alveolar thickening, and structural disruption, supporting the conclusion that both cytokines contribute to the immunopathology seen in this model.
Together, these additional analyses strengthen our findings by demonstrating that targeting IL-17 and IL-1β not only alters cellular and cytokine responses but also confers measurable protection from virus-induced disease (page 12, lines 450 – 452).

15. How do the authors explain the fact that while IFNa is reduced, IFNb is increased? What impact could that have to their results

Response: The divergent expression of type I interferons observed in our study (reduced IFNα but increased IFNβ) is consistent with differences in their regulation and cellular sources. IFNβ is uniquely inducible by NF-κB, which is strongly activated in the context of elevated IL-1β. In contrast, IFNα (and IFNλ) expression is primarily regulated through IRF7 activation. The increase in IFNβ observed in our model likely reflects the elevated NF-κB activity and excessive IL-1β production by Nox2-deficient neutrophils.
Indeed, a positive correlation between IL-1β and IFNβ production has been previously described (Wang et al., 2010 https://doi.org/10.4049/jimmunol.1000114). However, despite increased IFNβ, we observe no reduction in viral load in Ly6G-Cre Nox2 flox mice. This suggests that IFNβ alone is insufficient to mount a protective antiviral response in this model of IAV infection, and that other components of the type I interferon system, particularly IFNα, are likely critical for efficient viral control.
Thus, the observed cytokine imbalance (high IL-1β and IFNβ, low IFNα) may reflect a dysregulated innate immune response that fails to clear virus effectively, while simultaneously promoting immunopathology (page 6, lines 205 – 207 and 217 - 219).

16. In the Fig 4J legend, it should be mentioned that this is p50 DNA binding activity.

Response: Figure legends Figure 4K (former 4J) and 5K have been updated accordingly.

17. The introduction is rather long and should be shortened and streamlined

Response: We have shortened and restructured the introduction to improve clarity and focus. At the same time, we incorporated a new paragraph detailing the function and composition of Nox2 subunits, as requested by Reviewer #4. We believe these changes enhance both the readability and relevance of the introduction adjusted to the manuscript's focus.

18. There are no page numbers or line numbers, making it difficult to indicate what specific comments concern.

Response: We have added page and line numbers in the revised manuscript.

Reviewer #4 (Remarks to the Author)

The research article entitled "Neutrophil NADPH oxidase breaks the inflammatory IL-1β/IL-17A circuit to enhance pathogen clearance during respiratory virus infections" by

Aderonke Sofoluwe, Angelos Petropoulos, Abhilesh Salil Goomanee, Zehra Fatima Ali-Khan and Annika Warnatsch, describes the results of a study aimed at investigating the role of the neutrophil NADPH oxidase NOX2 in Influenza A Virus (IAV) infection-induced inflammation. Using a Ly6G-Cre(+/-)/tdTomato Nox2 flox mouse, the results show that after IAV infection of these mice, ROS production was decreased, IL-1β and IL-17A production was increased, neutrophil-derived ROS affect γδ T cell proliferation and function, In the absence of neutrophil Nox2, increased neutrophil-derived IL-1β drives proliferation of IL-17 producing gamma delta (γδ) T cells (γδ17 T cells). This early enhancement of the IL-β/IL-17 axis counteracts the antiviral interferon response in IAV-infected mice. They then extended their findings to humans. Similar patterns of ROS production and cytokine regulation were observed in human neutrophils when exposed to the virus analogue poly(I:C) and SARS-CoV-2. The authors conclude that their data demonstrate that ROS, which are often associated with damage, play a dual role in regulating cytokine signalling and thus influencing the immune response to respiratory viruses. In general, the results are new and interesting, the presentation of the data is clear, but there are some points that need to be verified.

We appreciate the careful review and valuable suggestions provided by Reviewer #4. Below, we respond to each comment and highlight the specific amendments made in the revised version of the manuscript.

Specific comments:

1-All mouse results are based on the use of Ly6G-Cre(+/-)/tdTomato Nox2 flox mice, assuming that the Ly6G gene is specific for neutrophils. However, several recent studies have shown that macrophages, monocytes and myeloid-derived suppressor cells (MDSC) also express this protein (e.g. see Ruscitti C et al, Sci Immunol 2024,9,98,eado1227). The authors should purify all these cells from control and Ly6G-Cre(+/-)/tdTomato Nox2 flox mice and check the expression of NOX2/gp91phox by Western blot in uninfected and IAV-infected mice. Good quality commercial antibodies against NOX2/gp91phox are available.

Response: We thank the reviewer for this important point. Indeed, we are aware of recent studies suggesting Ly6G expression can occur in a subset of monocyte-derived cells, particularly in later stages of IAV infection. However, our study focuses on the early acute phase of IAV infection (day 1 – 5 post infection), whereas Ruscitti et al. (https://doi.org/10.1126/sciimmunol.ado1227) primarily observed Ly6G$^+$ macrophages in the resolution phase (day 10).

To address the reviewer's concern, we conducted detailed flow cytometry analyses at day 3 post-infection, assessing the frequency and total numbers of Ly6G$^+$ CD64$^+$ double-positive macrophages, classical CD64$^+$ macrophages, monocytes and Ly6G$^+$ neutrophils (Figures 2B and C, S4G – J). Our results show that:

- Ly6G$^+$ CD64$^+$ macrophages represent only 2.6 ± 2 % and 2.0 ± 0.8 % of CD11b$^+$ leukocytes in Ly6G-Cre and Ly6G-Cre Nox2 flox mice, respectively. While

classical macrophages make up 43.1 ± 8.3 % and 53.3 ± 7.8 % of Live $CD45^+$ $CD11b^+$ cells.

- Total counts of $Ly6G^+CD64^+$ cells are more than 20-fold lower than classical macrophages and neutrophils ($1.25 \pm 0.7 \times 10^5$ and $1.25 \pm 0.6 \times 10^5$ $Ly6G^+CD64^+$ cells per 100 mg lung tissue, versus $2.5 \pm 0.7 \times 10^6$ and $3.9 \pm 1.0 \times 10^6$ classical macrophages ($Ly6G^-$ $CD64^+$) in Ly6G-Cre and Ly6G-Cre Nox2 flox mice, respectively.)
- Importantly, there is no difference in the frequency or number of $Ly6G^+$ macrophages between Ly6G-Cre and Ly6G-Cre Nox2 flox mice.

Due to their low abundance, we were unable to sort $Ly6G^+$ $CD64^+$ cells for reliable downstream qPCR or Western blot analysis. However, to confirm specificity of Nox2 deletion, we performed qPCR on FACS-sorted populations, showing that Nox2 is efficiently deleted in neutrophils ($CD11b^+$ $Ly6G^+$ $CD64^-$) but retained in classical macrophages ($CD11b^+$ $CD64^+$) and monocytes ($CD11b^+$ $Ly6C^{hi}$ $Ly6G^-$) (Figure S1C and D). We believe that our data strongly support that Nox2 deletion is confined to neutrophils in our model during the time frame studied. Results were added on page 4, lines 120 - 130 of the revised manuscript.

2-Figure 1A: Looking more closely at the Figure 1A-NOX2-/- image, there is more DHE staining than in Ly6G-Cre(+/-) Nox2 flox mice? How can this be explained?

Response: The slightly elevated DHE signal in the Nox2-/- panel of Figure 1A is likely due to background staining and compensatory activity of other ROS-generating enzymes, such as Nox1 and Nox4, xanthine oxidase or mitochondria, which are not affected by Nox2 deletion. These systems may maintain baseline ROS levels independent of NADPH oxidase 2.

Crucially, quantitative flow cytometry data (Figures 1B and 1C) show a significant reduction in ROS production in leukocytes of Ly6G-Cre Nox2 flox and Nox2-/- mice, confirming the expected functional impact of Nox2 deletion.

However, in Figure 1D, we observed that baseline CellROX intensity in the $CD45^-$ compartment of Nox2-/- mice was elevated, suggesting that compensatory ROS production via Nox2-independent pathways may occur in these mice, particularly in non-immune or structural lung cells. These pathways could lead directly to hydrogen peroxide production, bypassing the Nox2-dependent superoxide generation. Importantly, IAV infection did not further increase CellROX staining in Nox2-/- mice. We have clarified this point in the manuscript page 4 line 145.

3-Figure 1E: Virus copy number: Looking closer at Figure 1E-day 3, there is more virus in Ly6G-Cre(+/-) Nox2 flox mice (about 1 million/microL) than in Nox2-/- and control mice (10 000/microL (100 times more))? How can this be explained? These data differ from those reported in the literature. Also, in all experiments, Nox2-/- should be compared with WT mice.

Response: Previous studies comparing IAV infection in global Nox2-/- mice with WT controls (Vlahos et al., 2011; https://doi.org/10.1371/journal.ppat.1001271; Snelgrove et al., 2006; https://doi.org/10.1002/eji.200635977) reported a marginal, non-significant reduction in viral loads. A potential explanation for this observation is that Nox2-derived ROS support certain stages of viral entry and replication. Indeed, it has been shown that IAV exploits ROS for efficient infection: Nox2 deficiency in epithelial cells has been linked to reduced viral entry (Sgarbanti et al., 2011; https://doi.org/10.1089/ars.2010.3512) and replication has been shown to depend on redox-sensitive pathways (Amatore et al., 2015; https://doi.org/10.1111/cmi.12343). Thus, in global Nox2-/- mice, a lack of Nox2 in epithelial and other non-immune cells could impair virus propagation directly.

In contrast, our study focuses on neutrophil-specific Nox2 deletion using Ly6G-Cre Nox2 flox mice, which allows us to isolate the immunoregulatory role of neutrophil-derived ROS without interfering with ROS-dependent viral entry or replication. In this context, we observe a higher viral load at day 3 post-infection, which correlates with increased production of IL-1β and IL-17 (Figures 2L and N, 3A - F). This cytokine signature promotes excessive recruitment of neutrophils and monocytes, contributing to lung tissue damage (Figures 2A - C and S2C - E) and compromised epithelial barrier function, which in turn facilitates deeper viral spread into lung tissue. Moreover, IL-1β and IL-17 inhibit the upregulation of type I interferon alpha, undermining the antiviral response (Figures 2M and O, 6A - G). We now highlight these distinctions in the revised Discussion section page 15 line 574 – 585 and 592 - 599.

Importantly, all experiments used littermate controls to ensure appropriate comparison within the same genetic background. While global Nox2-/- and WT comparisons are valuable, our data focus on the neutrophil-specific mechanism by which Nox2 restricts excessive inflammation and supports early antiviral defense. We have added the comparison between WT and Nox2 -/- mice to figures 1E and S2A.

4-In Figure 1A,B,C,D: why was Ly6G-/- used as a control and not Ly6G+/- as in Figure 1F and Figure 2?

Response: We compare Ly6G-Cre(+/-) Nox2 flox mice to both Ly6G-Cre(-/-) Nox2 flox and Ly6G-Cre(+/-) littermate controls. The Ly6G-Cre(+/-) mice additionally express tdTomato in neutrophils, which facilitates flow cytometry and immunofluorescence-based analyses, as used in figures 1F and 2. Thus, their use provides a technical advantage in identifying and tracking neutrophils in specific applications, while maintaining appropriate background controls. By using all available littermates in our analyses, we adhere to the principles of the 3Rs (Replacement, Reduction, and Refinement), specifically reducing animal use by maximising the data obtained from each cohort. Key experiments were carried out in both controls (Figure S2A).

5-Why was the TNF concentration increased in Figure 2F but not the TNF mRNA in Figure 2?

Response: In our study, TNF-α protein levels were measured in bronchoalveolar lavage (BAL) fluid (Figure 2L, former F), whereas TNF-α mRNA was measured in whole lung tissue (Figure 2N, former E). The observed discrepancy likely reflects differences in post-transcriptional and post-translational regulation of TNF-α. There is well-established evidence that TNF-α protein release is tightly controlled at the post-translational level, particularly through the proteolytic cleavage of membrane-bound TNF-α by ADAM17. This cleavage produces the soluble, biologically active form of TNF-α, which is rapidly released into the extracellular space (Black et al. 1997; https://doi.org/10.1038/385729a0). IAV infection activates ADAM17 promoting TNF-α shedding independently of increased transcription (DeBerge et al. 2013; https://doi.org/10.1371/journal.pone.0079340). Thus, elevated TNF-α protein levels in BAL fluid may result from enhanced processing and release, rather than increased gene expression, and this could explain the observed divergence between mRNA and protein levels. We have added this information to the results description page 6 lines 202 – 204.

6-figure 3K: The absence of Nox2 induced an increase in neutrophil T-cell interaction, how can this be explained?

Response: We have removed the Imagestream analysis previously used to assess neutrophil–γδ T cell interactions in lung tissue, following another reviewer's suggestion. The technique is limited in its ability to accurately reflect physiological cell–cell interactions due to artefacts introduced by lung tissue processing, such as enzymatic digestion and mechanical disruption.

Instead, we now focus on immunofluorescence staining of lung sections (Figure 3K), which demonstrates spatial proximity of neutrophils and γδ T cells near sites of IAV infection. In parallel, classical flow cytometry revealed that in the absence of neutrophil-derived Nox2, γδ T cells exhibit reduced oxidative stress (Figures 3I and J), which correlates with increased γδ T cell numbers (Figure 3E), enhanced proliferation, and reduced apoptosis (Figure 3H).

Furthermore, in ex vivo co-culture systems, we directly observed physical contact between neutrophils and γδ T cells. When co-cultured with Nox2-competent neutrophils, γδ T cells undergo oxidative stress (as shown by CellROX staining in Figures 4D and 4E). This likely contributes to γδ T cell apoptosis and a reduction in their numbers, thereby limiting the duration of neutrophil–γδ T cell interactions. In contrast, Nox2-deficient neutrophils fail to induce ROS-mediated stress but instead promote γδ T cell proliferation via IL-1β release (Figure 4H - J).

Altogether, these findings support the conclusion that in the absence of neutrophil-derived ROS, γδ T cells survive longer, retain IL-17 production capacity, proliferate and remain in prolonged contact with neutrophils.

7-figure 3J: In control cells, LT are more stained than neutrophils? This is not normal as neutrophils produce more ROS. In all Figures 3 uninfected versus AIV-infected mice should be shown.

Response: The CellROX dye used in our experiments measures oxidative stress, not ROS production per se. It is a cell-permeant probe that becomes fluorescent upon oxidation by reactive oxygen species, particularly H_2O_2, and subsequent binding to DNA. Therefore, the intensity of CellROX signal reflects the net oxidative stress experienced by a cell, which depends on both the level of ROS exposure and the cell's antioxidant capacity.

While neutrophils are potent producers of ROS, they also express a robust antioxidant machinery that tightly regulates intracellular oxidative stress. In contrast, γδ T cells - especially IL-17-producing subsets - are known to have limited antioxidant defences and are more susceptible to oxidative damage. This has been demonstrated by Mensurado et al. (https://doi.org/10.1371/journal.pbio.2004990), who showed that IL-17–producing γδ T cells lack sufficient antioxidant response and are particularly sensitive to neutrophil-derived ROS such as H_2O_2. Thus, the higher CellROX signal in γδ T cells compared to neutrophils likely reflects greater oxidative stress due to limited detoxification capacity, rather than ROS production.

In response to another reviewer's comments, we have removed the Imagestream analysis as it is not suitable to analyse cell interactions in tissue. Instead, we have now included images of mock-infected γδ T cells and neutrophils in Figure 4D, allowing direct comparison of CellROX staining between mock and IAV infection.

8-Figure 4A and Figure 5C: To detect NADPH oxidase activation, the authors measured p47phox and p67phox colocalisation at the membrane. These cytosolic proteins are known to interact in the cytosol even in resting cells, colocalisation with Nox2 or p22phox is required to confirm activation.

Response: We have revised our approach to better reflect NADPH oxidase activation. Specifically, in Figure 4A and B, we now show colocalisation of gp91phox (Nox2) with p47phox in murine neutrophils, a more definitive marker for active complex assembly at the membrane. We observed a peak in colocalisation (Pearson's R coefficient) at 1-hour post-IAV infection. We have changed the results section accordingly page 8 lines 317 - 321. Similarly, for human neutrophils, we now present updated images and quantification in figures 5A-C and S13B, page 10 lines 396 - 401. Peak colocalization post poly(I:C) stimulation is observed after 30 minutes (figure 5A and B), and 1 hour post IAV infection (figure 5C and S13B). These data provide stronger support for NADPH oxidase activation following viral stimulation in both mouse and human neutrophils.

9-Figure 4G: Same point as in "7": In control WT cells, LT are more stained than neutrophils? This is not normal as neutrophils produce more ROS. All Figure 4 should show resting versus activated cells. Same for Figure 5G.

Response: Micrographs of mock-infected γδ T cells and neutrophils are now included in Figure 4D, enabling direct comparison of CellROX staining between mock and IAV infection. In response to another reviewer's request, the Imagestream analysis previously shown in figure 5G was removed.

10- The effect of hydrogen peroxide (H2O2) on IL-1 and IL-17 production by LT and neutrophils should be tested.

Response: We thank the reviewer for this valuable suggestion. To assess the impact of H_2O_2 on cytokine production, we treated human γδ T cells with H_2O_2 and measured IL-17 production. As shown in Supplemental Figure S13E, treatment with H_2O_2 completely abolished IL-17 expression, which was already low in PBS-treated control cells. This suggests that elevated oxidative stress impairs cytokine production by γδ T cells. We further treated mock- and IAV-infected γδ T cells with superoxide dismutase (SOD) and catalase to scavenge H_2O_2. This intervention significantly increased IL-17 release, supporting the notion that oxidative stress suppresses IL-17 production (Figure S13F). These findings corroborate that H_2O_2 negatively regulates IL-17 expression in γδ T cells, further supporting our central hypothesis that Nox2-derived ROS modulates γδ T cell responses during IAV infection. Added to results section page 11 lines 425 – 428.

11-Minors:
a)-Introduction: a detailed paragraph on the NADPH oxidase subunits should be added to the introduction.

Response: We have restructured the introduction and added a paragraph detailing the function and composition of Nox2 subunits on page 2 lines 65 - 69.

b)-Figure 2A-E: The x-axis should be labelled (time (days)??).

Response: We have added x-axis labels in Figure2A – E.

c)-Figure 4G: Why was IL-17 increased in WT neutrophils in Transwell?

Response: Our data demonstrate that two distinct mechanisms contribute to neutrophil Nox2-mediated γδ T cell activation. Firstly, both WT and Nox2-deficient (KO) neutrophils can stimulate γδ T cells via IL-1beta release, which in turn supports IL-17 production from γδ T cells. However, Nox2-deficient neutrophils produce significantly more IL-1b, as shown by increased transcription driven by NF-kB activation (Figures 4I – K). This represents the first mechanism of γδ T cell activation.

A second mechanism involves proximity-dependent ROS transfer from neutrophils to γδ T cells. In direct co-cultures with WT neutrophils, hydrogen peroxide is locally transferred to γδ T cells, inducing oxidative stress in γδ T cells and suppressing their function. When neutrophils are physically separated from γδ T cells by a Transwell membrane, this contact-dependent ROS transfer is prevented, resulting in diminished oxidative stress and IL-17 production increases. This finding is supported by various previous reports, for example Petzold et al. (https://doi.org/10.1016/j.immuni.2022.10.001) showed that neutrophils regulate megakaryocyte function via contact-dependent ROS transfer. Pillay et al. (https://doi.org/10.1172/JCI57990) demonstrated that H_2O_2 suppresses T cell proliferation via Mac-1-dependent cell contact.

In contrast, KO neutrophils do not generate sufficient amounts of ROS and thus do not induce oxidative stress in γδ T cells, regardless of proximity. Therefore, in the absence of ROS-mediated suppression the IL-1b-driven stimulatory affects are unmasked leading to enhanced IL-17 production.

In summary, both WT and KO neutrophils can activate γδ T cells through IL-1b. However, Nox2-deficient neutrophils show augmented IL-1b production and lack ROS-mediated suppressive effect, resulting in a net increase in γδ T cell IL-17 production. We have added additional clarification on page 9 lines 351 – 358 and page 10 lines 373 – 379.

REVIEWER COMMENTS

Reviewer #3 (Remarks to the Author):

The manuscript by Petropoulos et al is improved. However, some issues remain. Also, the lack of use of 'track changes' marking in the revised version makes the assessment of the revised version quite difficult.

Specific points

1. This point is not well addressed. The authors reply that 'Mrp8-Cre targets a broader population of myeloid cells, including monocytes, macrophages and dendritic cells' is not right. Where do they base that. It is well established that Mrp8-Cre mice are the most specific Cre tools for deleting something in neutrophils, and would have been more appropriate as they do not hit macrophages.

The fact that Ly6G-Cre target a subpopulation of macrophages, although small early on (but under expansion as it can reach up to 50% of all macrophages according to the original publication) does not mean they are not important in terms of the effects observed.

Also, a population of 2-3% of all CD45+CD11b+ cells does not mean they are rare enough that they cannot be sorted and analyzed (for Nox2 deletion). Anyway, at the minimum the authors should introduce a comment in the discussion indicating these limitations.

The statement regarding broader targeting by Mrp8-Cre is based on published analyses of Cre activity in MRP8-Cre lines. While these mice show strong activity in neutrophils, promoter activity outside mature neutrophils has been reported: Abram et al. (2014) demonstrated Cre activity in 10 – 20% of granulocyte–macrophage progenitors (GMPs). As GMPs give rise to monocytes and macrophages, this indicates recombination is not confined to terminally differentiated neutrophils. Further, earlier work by Passegué et al. (2004) using the Mrp8-Cre line has shown activity extending across granulocyte and macrophage compartments. Similar findings are reported for LysM-Cre mice (Clausen et al., 1999; Faust et al., 2000).
In addition, targeted locus amplification analysis of the MRP8-Cre line demonstrated random integration into chromosome 5 resulting in potential confounding effects, particularly under inflammatory conditions (Wang et al., 2022). We have now clarified this in the revised manuscript by adding the corresponding references on page 3, line 113.

We agree that Ly6G^{+} macrophages represent a potential caveat. This population comprised ~2 – 3% of CD45^{+}CD11b^{+} cells during days 1 – 3 post-infection and functional contributions cannot be excluded. A statement acknowledging this limitation has been added to the Discussion on page 13, lines 494 – 500.

1) Abram CL, Roberge GL, Hu Y, Lowell CA. Comparative analysis of the efficiency and specificity of myeloid-Cre deleting strains using ROSA-EYFP reporter mice. J Immunol Methods. 2014 Jun;408:89-100. doi: 10.1016/j.jim.2014.05.009. Epub 2014 May 22. PMID: 24857755; PMCID: PMC4105345.
2) Passegué E, Wagner EF, Weissman IL. JunB deficiency leads to a myeloproliferative disorder arising from hematopoietic stem cells. Cell. 2004 Oct 29;119(3):431-43. doi: 10.1016/j.cell.2004.10.010. PMID: 15507213.

3) Clausen BE, Burkhardt C, Reith W, Renkawitz R, Förster I. Conditional gene targeting in macrophages and granulocytes using LysMcre mice. Transgenic Res. 1999 Aug;8(4):265-77. doi: 10.1023/a:1008942828960. PMID: 10621974.
4) Faust N, Varas F, Kelly LM, Heck S, Graf T. Insertion of enhanced green fluorescent protein into the lysozyme gene creates mice with green fluorescent granulocytes and macrophages. Blood. 2000 Jul 15;96(2):719-26. PMID: 10887140.
5) Wang G, Zhang C, Kambara H, Dambrot C, Xie X, Zhao L, Xu R, Oneglia A, Liu F, Luo HR. Identification of the Transgene Integration Site and Host Genome Changes in MRP8-Cre/ires-EGFP Transgenic Mice by Targeted Locus Amplification. Front Immunol. 2022 Apr 6;13:875991. doi: 10.3389/fimmu.2022.875991. PMID: 35464448; PMCID: PMC9020256.

2. Unfortunately, again this argument is not easy to understand. It is not clear why in the initially submitted manuscript the fact that the gating strategy was based on Live CD45$^+$ CD11b$^+$ Gr-1$^+$ blood cells (initial Figure S1B) should give different results than when CD45$^+$ CD11b$^+$ cells were used instead. Why Nox2 expression was previously also coming present n neutrophils from Ly6GCre-/- mice but now that's not the case?

The new data of Suppl. Fig. S1C and D are good but they only come from Ly6G-Cre+/- Nox2fl/fl mice. They should include Ly6G-Cre-/- Nox2fl/fl mice as this is an important control especially in view of the previous submission indicating potential deletion of Nox2 in neutrophils from these mice as well.

We thank the reviewer for carefully examining this point. Upon re-evaluation of the intracellular Nox2-FITC staining data, we concluded that this approach was not reliable to assess Nox2 deletion in our system.

Specifically, when we repeated the analysis, we observed substantial variability in Nox2-FITC signal intensity between experiments and between individual mice of the same genotype.

The attached figure shows our full gating strategy for Ly6G-Cre(+/-) Nox2 flox/flox, Ly6G-Cre(+/-) Nox2 flox/-, Ly6G-Cre(-/-) Nox2 flox/flox and Ly6G-Cre(+/-) mice, plotting tdTomato versus intracellular Nox2-FITC staining within the Live CD45$^+$CD11b$^+$, Live CD45$^+$CD11b$^+$Gr1$^+$ and Live CD45$^+$CD11b$^+$Gr1$^-$ populations:

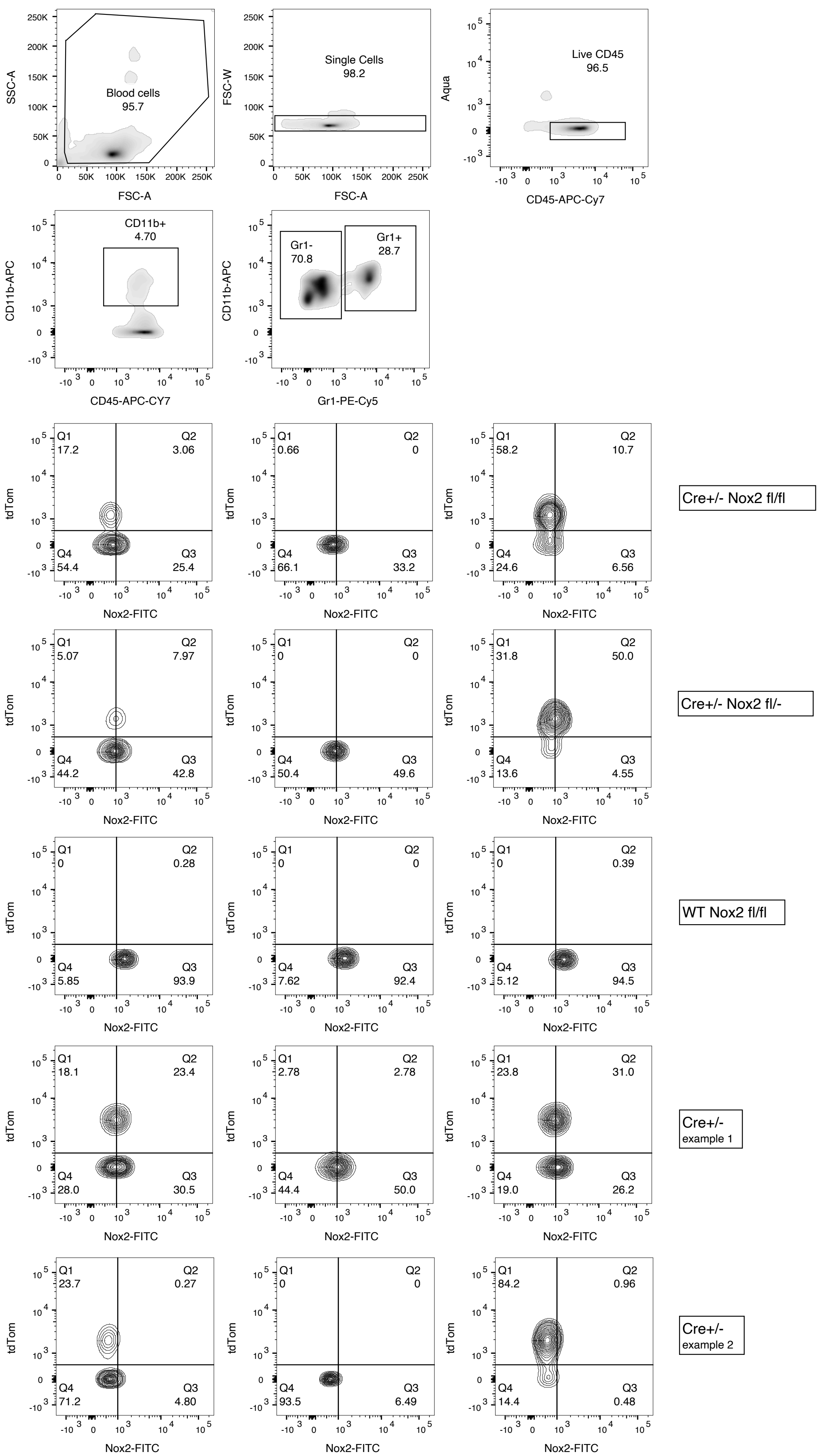

Blood cells
95.7
SSC-A
FSC-A
Single Cells
98.2
FSC-W
FSC-A
Live CD45
96.5
Aqua
CD45-APC-Cy7
CD11b+
4.70
CD11b-APC
CD45-APC-CY7
Gr1-
70.8
Gr1+
28.7
CD11b-APC
Gr1-PE-Cy5
Q1 17.2
Q2 3.06
Q4 54.4
Q3 25.4
Q1 0.66
Q2 0
Q4 66.1
Q3 33.2
Q1 58.2
Q2 10.7
Q4 24.6
Q3 6.56
Cre+/- Nox2 fl/fl
Q1 5.07
Q2 7.97
Q4 44.2
Q3 42.8
Q1 0
Q2 0
Q4 50.4
Q3 49.6
Q1 31.8
Q2 50.0
Q4 13.6
Q3 4.55
Cre+/- Nox2 fl/-
Q1 0
Q2 0.28
Q4 5.85
Q3 93.9
Q1 0
Q2 0
Q4 7.62
Q3 92.4
Q1 0
Q2 0.39
Q4 5.12
Q3 94.5
WT Nox2 fl/fl
Q1 18.1
Q2 23.4
Q4 28.0
Q3 30.5
Q1 2.78
Q2 2.78
Q4 44.4
Q3 50.0
Q1 23.8
Q2 31.0
Q4 19.0
Q3 26.2
Cre+/-
example 1
Q1 23.7
Q2 0.27
Q4 71.2
Q3 4.80
Q1 0
Q2 0
Q4 93.5
Q3 6.49
Q1 84.2
Q2 0.96
Q4 14.4
Q3 0.48
Cre+/-
example 2
tdTom
Nox2-FITC

This variability likely reflects several technical limitations of intracellular Nox2 detection by flow cytometry such as epitope accessibility (fixation and permeabilization can influence antibody access and vary between preparations), activation-dependent localisation of Nox2 (subcellular distribution of gp91phox can alter antibody binding and fluorescence intensity), changes in granule composition (with neutrophil maturation and activation state detectable protein levels can vary independent of gene deletion) as well as signal-to-noise limitations. Importantly, two independent Ly6G-Cre(+/-) mice displayed differing Nox2-FITC staining patterns despite identical genotypes, further indicating that the assay lacks reproducibility for quantitative interpretation.

Given these limitations, we concluded that intracellular Nox2 protein staining assessed by flow cytometry is not an appropriate method to determine deletion efficiency in this context. We therefore removed this dataset to avoid misinterpretation and thank the reviewer for highlighting this issue.

To conclusively validate deletion specificity in our system, we instead extended our sorting strategy to include Ly6G-Cre(-/-) Nox2 flox as well as Ly6G-Cre(+/-) controls and performed qPCR analysis of *Cybb* gene expression. As shown in revised Figure S1B (including gating strategy for flow sorting and *Cybb* qPCR), this approach demonstrates clear and specific Nox2 ablation in neutrophils from Ly6G-Cre(+/-) Nox2 flox mice, whereas Cre-negative controls as well as unfloxed Ly6G-Cre(+/-) retain normal *Cybb* expression. We conclude that the mRNA-based analysis provides a more reliable and quantitative assessment of gene deletion in this context. The manuscript has been updated accordingly on page 4 lines 123-129.

3. This is insufficiently addressed as all gating strategies, and wherever needed in both Ly6G-Cre+/- Nox2fl/fl mice and Ly6G-Cre-/- Nox2fl/fl mice -in the various conditions- should be shown. Graphs can be short in size and fit but they are important as they convince the reader there are no other changes, (e.g. population shifts) affecting the results.

In 6E, even if intracellular cytokine staining plots for additional cell subsets, for macrophages the isotype control (for IL-1b and TNF) stained cells should be shown.

We have now added FACS plots for the sorting of myeloid cell populations for qPCR analysis (figure S1), CellROX staining of CD45 positive and negative cell populations (figure S2), for all lung cell populations analysed in Ly6G-Cre(+/-) and Ly6G-Cre(+/-) Nox2 flox mice (figure S3 and S4), for all intracellular cytokine stainings for Ly6G-Cre(+/-) and Ly6G-Cre(+/-) Nox2 flox mice (Figure S6), as well as isotype staining controls for IL-1b, IL-17A and TNFa with corresponding control IgGs (figure S6).

4. Although the authors have corrected the text, the main issue with respect to the mechanism proposed, i.e. the claim that 'this early self-amplified augmentation of the IL-1β/IL-17 axis counteracts the antiviral interferon response against IAV infection in mice.' remains This is also in the abstract. If IFNλ is not affected, IFNb is increased and only IFNa is reduced, how can the authors claim that?

A possibility is to claim that this is due to the IFNa decrease but will not be convincing enough for many. Have the authors examined the expression of ISFs, especially in the lung epithelium?

In any way, this needs to be corrected.

We agree that the original wording overstated the conclusion regarding the antiviral interferon response. To directly address this concern, we performed additional qPCR analyses of interferon-stimulated genes (ISGs) in whole lung tissue at day 2 post-infection. As shown in newly added Figure S5C - E, we observed significantly reduced expression of canonical ISGs, including *Isg15*, *Ifit3*, and *Oas1a*, in Ly6G-Cre(+/-) Nox2 flox mice compared to Ly6G-Cre(-/-) Nox2 flox controls. These data indicate that despite differential regulation of individual interferon subtypes, downstream antiviral interferon signalling is functionally impaired.
These new results are now included in the manuscript (page 6, lines 215 – 216).
In addition, we have revised the wording in the Highlights (page 1, lines 18 – 19) and in the Abstract (page 1, lines 33 – 34) to avoid overgeneralisation and to more precisely reflect that the IL-1β/IL-17 axis is associated with impaired IFNa signalling, rather than suppressing all interferon species.

REVIEWER COMMENTS

Reviewer #3 (Remarks to the Author):

The manuscript by Petropoulos et al is improved. However, some issues remain. Also, the lack of use of 'track changes' marking in the revised version makes the assessment of the revised version quite difficult.

Specific points

1. This point is not well addressed. The authors reply that 'Mrp8-Cre targets a broader population of myeloid cells, including monocytes, macrophages and dendritic cells' is not right. Where do they base that. It is well established that Mrp8-Cre mice are the most specific Cre tools for deleting something in neutrophils, and would have been more appropriate as they do not hit macrophages.

The fact that Ly6G-Cre target a subpopulation of macrophages, although small early on (but under expansion as it can reach up to 50% of all macrophages according to the original publication) does not mean they are not important in terms of the effects observed.

Also, a population of 2-3% of all CD45+CD11b+ cells does not mean they are rare enough that they cannot be sorted and analyzed (for Nox2 deletion). Anyway, at the minimum the authors should introduce a comment in the discussion indicating these limitations.

The statement regarding broader targeting by Mrp8-Cre is based on published analyses of Cre activity in Mrp8-Cre lines. While these mice show strong activity in neutrophils, promoter activity outside mature neutrophils has been reported: Abram et al. (2014) demonstrated Cre activity in 10 – 20% of granulocyte–macrophage progenitors (GMPs). As GMPs also give rise to monocytes and macrophages, this indicates recombination is not confined to terminally differentiated neutrophils. Further, earlier work by Passegué et al. (2004) using the Mrp8-Cre line has shown activity extending across granulocyte and macrophage compartments. Similar findings are reported for LysM-Cre mice (Clausen et al., 1999; Faust et al., 2000).
In addition, targeted locus amplification analysis of the Mrp8-Cre line revealed that the transgene had randomly integrated into chromosome 5, resulting in unintended genomic alterations that could cause confounding effects particularly under inflammatory conditions (Wang et al., 2022). We have reworded and clarified this in the revised manuscript by adding the corresponding references on page 3, lines 109 - 112.

We agree that Ly6G$^+$ macrophages represent a potential caveat. This population comprised ~2 – 3% of CD45$^+$CD11b$^+$ cells during days 1 – 3 post-infection and functional contributions cannot be excluded. A statement acknowledging this limitation has been added to the Results section page 4, lines 142 – 145 and Discussion on page 13, lines 511 – 517.

1) Abram CL, Roberge GL, Hu Y, Lowell CA. Comparative analysis of the efficiency and specificity of myeloid-Cre deleting strains using ROSA-EYFP reporter mice. J Immunol Methods. 2014 Jun;408:89-100. doi: 10.1016/j.jim.2014.05.009. Epub 2014 May 22. PMID: 24857755; PMCID: PMC4105345.
2) Passegué E, Wagner EF, Weissman IL. JunB deficiency leads to a myeloproliferative disorder arising from hematopoietic stem cells. Cell. 2004 Oct 29;119(3):431-43. doi: 10.1016/j.cell.2004.10.010. PMID: 15507213.

3) Clausen BE, Burkhardt C, Reith W, Renkawitz R, Förster I. Conditional gene targeting in macrophages and granulocytes using LysMcre mice. Transgenic Res. 1999 Aug;8(4):265-77. doi: 10.1023/a:1008942828960. PMID: 10621974.
4) Faust N, Varas F, Kelly LM, Heck S, Graf T. Insertion of enhanced green fluorescent protein into the lysozyme gene creates mice with green fluorescent granulocytes and macrophages. Blood. 2000 Jul 15;96(2):719-26. PMID: 10887140.
5) Wang G, Zhang C, Kambara H, Dambrot C, Xie X, Zhao L, Xu R, Oneglia A, Liu F, Luo HR. Identification of the Transgene Integration Site and Host Genome Changes in MRP8-Cre/ires-EGFP Transgenic Mice by Targeted Locus Amplification. Front Immunol. 2022 Apr 6;13:875991. doi: 10.3389/fimmu.2022.875991. PMID: 35464448; PMCID: PMC9020256.

2. Unfortunately, again this argument is not easy to understand. It is not clear why in the initially submitted manuscript the fact that the gating strategy was based on Live CD45⁺ CD11b⁺ Gr-1⁺ blood cells (initial Figure S1B) should give different results than when CD45⁺ CD11b⁺ cells were used instead. Why Nox2 expression was previously also coming present n neutrophils from Ly6GCre-/- mice but now that's not the case?

The new data of Suppl. Fig. S1C and D are good but they only come from Ly6G-Cre+/- Nox2fl/fl mice. They should include Ly6G-Cre-/- Nox2fl/fl mice as this is an important control especially in view of the previous submission indicating potential deletion of Nox2 in neutrophils from these mice as well.

We thank the reviewer for carefully examining this point and agree that the previous presentation lacked sufficient clarity. In the revised manuscript, we now include the complete gating strategy together with additional histogram plots in Supplemental Figure S1A and B.

The discrepancy between the original and revised analyses reflect limitations of the original Gr-1-based gating approach. Gr-1 detects both Ly6G and Ly6C epitopes and therefore does not exclusively identify neutrophils. In addition, Cre expression in Ly6G-Cre mice reduces surface Ly6G expression, further complicating interpretation when using Gr-1/Ly6G-based gating alone. For this reason, in the revised analysis we instead assessed Nox2 expression within the broader Live CD45+ CD11b+ population together with tdTomato reporter expression.

Importantly, our revised data further demonstrate that tdTomato expression incompletely overlaps with Ly6G staining. By immunofluorescence analysis of lung sections, we observed tdTomato+ Ly6G+ cells, but also tdTomato− Ly6G+ and tdTomato+ Ly6G− cells within the same Ly6G-Cre(+/-) as well as Ly6G-Cre(+/-) Nox2 flox animals (Supplemental Figure S1C and D). These findings indicate that tdTomato reporter expression does not fully capture all Ly6G-lineage cells and therefore complicates interpretation of flow cytometry analyses based solely on tdTomato or Ly6G/Gr-1 expression.

Furthermore, the revised flow cytometric analyses demonstrated altered Nox2-FITC signal distributions not only in Ly6G-Cre(+/−) Nox2 flox mice, but also in Ly6G-Cre(+/−) controls lacking the floxed Nox2 allele, indicating that reporter/Cre status itself influences interpretation of Nox2 expression by flow cytometry. We therefore conclude that flow cytometric assessment of Nox2 protein expression alone is insufficient to reliably determine deletion specificity in this model.

To more conclusively validate deletion specificity in our model, we therefore extended our sorting strategy to include Ly6G-Cre(-/-) Nox2 flox as well as Ly6G-Cre(+/-) controls and performed qPCR analysis of *Cybb* gene expression. As shown in revised Figure S2A and B (including gating strategy for flow sorting and *Cybb* qPCR), this approach demonstrates clear and specific Nox2 ablation in neutrophils from Ly6G-Cre(+/-) Nox2 flox mice, whereas Cre-negative Ly6G-Cre(-/-) Nox2 flox controls and unfloxed Ly6G-Cre(+/-) controls retained normal *Cybb* expression. Monocytes and macrophages from Ly6G-Cre(+/-) Nox2 flox mice similarly retained *Cybb* expression comparable to controls. We therefore conclude that the revised mRNA-based analysis provides a more reliable and quantitative assessment of gene deletion in this context. The manuscript has been updated accordingly from page 3 line 115 to page 4 line 153.

3. This is insufficiently addressed as all gating strategies, and wherever needed in both Ly6G-Cre+/- Nox2fl/fl mice and Ly6G-Cre-/- Nox2fl/fl mice -in the various conditions- should be shown. Graphs can be short in size and fit but they are important as they convince the reader there are no other changes, (e.g. population shifts) affecting the results.
In 6E, even if intracellular cytokine staining plots for additional cell subsets, for macrophages the isotype control (for IL-1b and TNF) stained cells should be shown.

We thank the reviewer for this important suggestion and agree that inclusion of representative gating strategies and controls throughout the manuscript substantially improves transparency and interpretation of the flow cytometry analyses.

In the revised manuscript, we have therefore expanded the supplemental data to include representative gating strategies and flow cytometry plots performed in both Ly6G-Cre(+/-) and Ly6G-Cre(+/-) Nox2 flox mice. Specifically, we now include:

- Gating strategy for lung myeloid cells and representative flow cytometry and histogram plots for tdTomato versus Nox2 expression (Figure S1A and B),
- Representative sorting gates for lung myeloid cell populations used for downstream qPCR analysis (Figure S2A),
- Representative flow cytometry and histogram plots for CellROX staining of CD45 positive and negative lung cell populations (Figure S3A),
- Representative flow cytometry plots for all lung cell populations analysed in Ly6G-Cre(+/-) and Ly6G-Cre(+/-) Nox2 flox mice (Figure S4 and S5K),
- Fluorescence minus one (FMO) controls for intracellular cytokine staining in neutrophils, monocytes and gd T cells (Figure 3A, D, G),
- Isotype controls for intracellular cytokine staining including IL-1beta, IL-17A and TNFalpha staining in the various cell populations analysed (Figure S7C).
- Representative flow cytometry plots of cytokine staining in macrophages, conventional dendritic cells and alveolar macrophages in Ly6G-Cre(+/-) and Ly6G-Cre(+/-) Nox2 flox mice (Figure S7D – G),
- Representative flow cytometry and histogram plots for Ki67, CellROX and Apotracker staining (Figure S10).

- For the in vitro co-culture experiments, we revised the presentation to focus on histogram-based analyses (Figures 4F and 5H), and additionally included representative flow cytometry plots together with mock (PBS)-based gating controls (Figures S12A, S13E and S14G).

We believe these additions provide a substantially more comprehensive overview of the gating strategies, population distributions and staining controls used throughout the study, and help address the reviewer's concern regarding potential population shifts or gating-related artefacts.

4. Although the authors have corrected the text, the main issue with respect to the mechanism proposed, i.e. the claim that 'this early self-amplified augmentation of the IL-1β/IL-17 axis counteracts the antiviral interferon response against IAV infection in mice.' remains This is also in the abstract. If IFNλ is not affected, IFNb is increased and only IFNa is reduced, how can the authors claim that?
A possibility is to claim that this is due to the IFNa decrease but will not be convincing enough for many. Have the authors examined the expression of ISFs, especially in the lung epithelium?
In any way, this needs to be corrected.

We agree that the original wording overstated the conclusion regarding the antiviral interferon response. To directly address this concern, we performed additional qPCR analyses of interferon-stimulated genes (ISGs) in whole lung tissue at day 2 post-infection. As shown in newly added panels in figure S6 C - E, we observed significantly reduced expression of canonical ISGs, including *Isg15*, *Ifit3*, and *Oas1a*, in Ly6G-Cre(+/-) Nox2 flox mice compared to Ly6G-Cre(-/-) Nox2 flox controls. These data indicate that despite differential regulation of interferon subtypes, downstream antiviral interferon signalling is functionally impaired.
These new results are now included in the manuscript (page 6, lines 232 – 233).
In addition, we have revised the wording in the Highlights (page 1, lines 18 – 19) and in the Abstract (page 1, lines 33 – 34) to avoid overgeneralisation and to more precisely reflect that the IL-1β/IL-17 axis is associated with impaired IFNalpha signalling, rather than suppressing all interferon species.
